# Natural killer cells in clinical development as non-engineered, engineered, and combination therapies

**DOI:** 10.1186/s13045-022-01382-5

**Published:** 2022-11-08

**Authors:** Nina Lamers-Kok, Denise Panella, Anna-Maria Georgoudaki, Haiping Liu, Didem Özkazanc, Lucia Kučerová, Adil Doganay Duru, Jan Spanholtz, Monica Raimo

**Affiliations:** Glycostem Therapeutics, Kloosterstraat 9, 5349 AB Oss, The Netherlands

**Keywords:** Natural killer cells, NK cell therapies, Adoptive cell therapy, Immunotherapy, Cancer, Off-the-shelf, Allogeneic, GMP manufacturing, CAR-NK cells, Genetic engineering, Combination therapy

## Abstract

Natural killer (NK) cells are unique immune effectors able to kill cancer cells by direct recognition of surface ligands, without prior sensitization. Allogeneic NK transfer is a highly valuable treatment option for cancer and has recently emerged with hundreds of clinical trials paving the way to finally achieve market authorization. Advantages of NK cell therapies include the use of allogenic cell sources, off-the-shelf availability, and no risk of graft-versus-host disease (GvHD). Allogeneic NK cell therapies have reached the clinical stage as ex vivo expanded and differentiated non-engineered cells, as chimeric antigen receptor (CAR)-engineered or CD16-engineered products, or as combination therapies with antibodies, priming agents, and other drugs. This review summarizes the recent clinical status of allogeneic NK cell-based therapies for the treatment of hematological and solid tumors, discussing the main characteristics of the different cell sources used for NK product development, their use in cell manufacturing processes, the engineering methods and strategies adopted for genetically modified products, and the chosen approaches for combination therapies. A comparative analysis between NK-based non-engineered, engineered, and combination therapies is presented, examining the choices made by product developers regarding the NK cell source and the targeted tumor indications, for both solid and hematological cancers. Clinical trial outcomes are discussed and, when available, assessed in comparison with preclinical data. Regulatory challenges for product approval are reviewed, highlighting the lack of specificity of requirements and standardization between products. Additionally, the competitive landscape and business field is presented. This review offers a comprehensive overview of the effort driven by biotech and pharmaceutical companies and by academic centers to bring NK cell therapies to pivotal clinical trial stages and to market authorization.

**Supplementary Information:**

The online version contains supplementary material available at 10.1186/s13045-022-01382-5.

## Introduction

Natural reactivity of peripheral blood lymphocytes against tumor- or virus-infected cells was first observed in mice and humans in the 1970s [1–3]. Natural killer (NK) cells are an essential part of tumor immunosurveillance thanks to their unique ability to recognize and kill aberrant cells without prior sensitization, using a sophisticated array of germline-encoded receptors. The “missing self” hypothesis, proposed by Kärre and Ljunggren in 1981, conceived the capacity of NK cells to recognize and eliminate cells that do not express self-major histocompatibility complex (MHC) class I molecules [4]. The molecular mechanisms driving this function were unraveled by the identification and characterization of key surface receptors in the following years. NK cell stimulation and effector activity depend on the integration of signals derived from activating and inhibitory receptors, thus protecting the host against aberrant cells, while preventing deleterious autoimmune responses. Loss of surface MHC, and simultaneous upregulation of stress ligands in tumor cells, shift the balance toward NK cell activation [5–7]. Target cell elimination is mediated directly by cytotoxic pathways, or indirectly through cytokine secretion. Killing occurs via secretion of lytic granules containing perforin and granzymes or by inducing death receptor-mediated apoptosis via the engagement of Fas ligand or tumor necrosis factor-related apoptosis-inducing ligand (TRAIL) [8].

Thanks to their highly cytotoxic, non-MHC-restricted effector function, NK cells have a high potential to be developed as immunotherapies against cancer. Adoptive transfer of ex vivo expanded autologous NK cells has been tested in early clinical trials to treat patients with renal cell carcinoma (RCC) [9], lymphoma [10, 11], breast [10–12], digestive [13], colon [14] and lung cancer [14]. Although treatment was well received and toxicity was moderate, very limited anti-tumor effect was observed. The major reason was “self” recognition by inhibitory Killer immunoglobulin-like receptors (KIR) on infused NK cells, matching the MHC class I on tumor cells and therefore blocking activation [15]. Additionally, patients were heavily pretreated prior to NK cell collection and therapy, which impaired cell expansion and function after infusion [16]. To overcome these limitations, the use of ex vivo activated allogeneic human leukocyte antigen (HLA)-mismatched NK cells was investigated. The first evidence of safety and efficacy of alloreactive NK cells was observed in 2002, when Ruggeri and colleagues showed that donor NK cells could prevent relapse and graft rejection in acute myeloid leukemia (AML) patients receiving HLA-mismatched donor hematopoietic transplantation without causing graft-versus-host disease (GvHD), mainly due to KIR incompatibility [17]. Later, Miller and colleagues showed that infusion of haploidentical, related donor NK cells in AML patients induced complete hematologic remission in 5 of 9 poor prognosis patients, after pretreatment with high-dose cyclophosphamide and fludarabine (Hi-Cy/Flu) and in the presence of interleukin (IL)-2 [18]. Since then, allogeneic NK cells have been widely investigated in clinical trials for the treatment of hematological malignancies [19, 20] and of solid tumors, including melanoma, breast cancer, ovarian cancer, neuroblastoma, RCC, colorectal cancer (CRC), and hepatocellular cancer (HCC) [21].

The therapeutic potential of allogeneic NK cell transfer encourages the development of “off-the-shelf” NK cell-based immunotherapies as effective, safe, and universal products. In this review, we summarize the major achievements and obstacles in developing successful allogeneic NK cell therapeutics for cancer as non-engineered, engineered, or combination therapies. With a focus on products that have reached the clinical stage as registered on ClinicalTrials.gov, the different cell sources for NK generation, the cell expansion methods and manufacturing platforms, and the clinical use of NK cells are discussed. Picking up the baton from Veluchamy et al., who exhaustively reviewed allogeneic NK-based clinical trials until March 2017 [22], this study focuses on the most recent studies with non-engineered NK cells (2017–2021) and on all trials registered until December 2021 where NK cells are engineered or used as combination therapies. Clinical trial outcomes are discussed in comparison with preclinical studies when possible. Additionally, an outlook on the manufacturing, quality, and regulatory challenges that must be overcome to succeed in product approval is given.

## Current clinical development of NK cell therapies

**Fig. 1:**
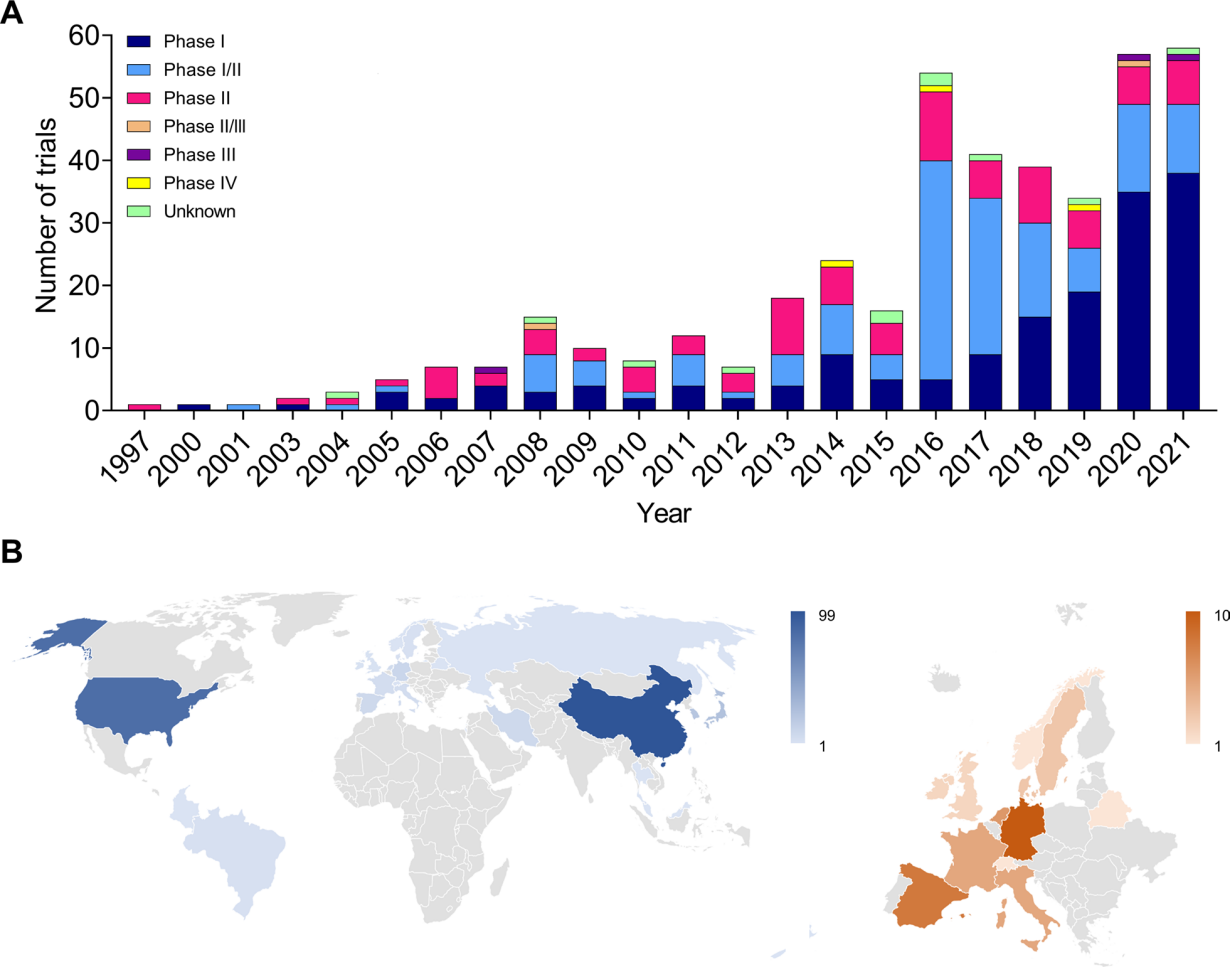
Overview of the NK cell therapy field. **A** Total number of NK cell-based clinical trials per year of initiation. Since 1997, a total of *N* = 420 clinical trials with NK cells have been initiated, the majority of which between 2016 and 2021. Most are Phase I and Phase I/II studies, *N* = 165 and *N* = 144, respectively. *N* = 92 studies are Phase II. As the field has just recently emerged, Phase II/III, III and IV trials are few (*N* = 2, *N* = 3, *N* = 3, respectively). For *N* = 11 trials, the clinical phase is unknown. **B** Geographical location of companies and institutions developing NK cell therapies. Color scales are representative of the number per country. Data sources: ClinicalTrials.gov, search terms: “NK cell,” “NK cell therapy,” “Natural killer cells” (text); GlobalData.com, drug descriptor: “Natural Killer (NK) Cell Immunotherapy” (drop-down menu), or filter: “Natural Killer cells” (text). The search is limited to December 2021. Clinical studies not based on administration of NK cells to patients or evaluating the infusion of mixed immune cell populations were excluded

The opportunity presented by NK cell immunotherapies has raised great interest in clinical product development, as shown by the increasing number of clinical trials registered globally in the last 25 years (Fig. 1A). As of December 2021, a total of 420 NK cell-based clinical trials have been reported (source: ClinicalTrials.gov and GlobalData.com). From 1997 to 2015, between 1 and 24 trials have been initiated per year (average: 8); only since 2016, numbers have increased enormously, reaching, on average, 47 per year. Since the field is relatively young, most trials are Phase I and Phase I/II studies. Several Phase II and few more advanced studies (Phase II/III, III, or IV, as registered in the Chinese clinical trial register) have been pursued. It is likely that in the next years several products will reach pivotal clinical stages and foreseeable market authorization. Accordingly, the promising safety and efficacy profile of NK cells makes them a booming business opportunity for investors. As of December 2021, the GlobalData analytics database reports a total of 295 institutions actively involved in the development of NK cell-based therapies worldwide. Of those, 117 are public or privately owned companies, while 178 are universities, hospitals, and research institutes. Geographical distribution (Fig. 1B) is most enriched in China (99 companies and institutions), followed by the USA (82) and Europe (47). In Europe, Germany hosts 10 organizations, Spain 8, the Netherlands 6, Italy and France 5, Denmark and Sweden 3, Ireland and the UK 2, Belarus, Norway, and Switzerland 1. The remaining 67 locations are in Japan (24), South Korea (21), Iran (8), Brazil (3), Taiwan and Singapore (2), and Hong Kong, Colombia, Israel, Malaysia, Thailand, New Zealand, and Russia (1).

**Fig. 2:**
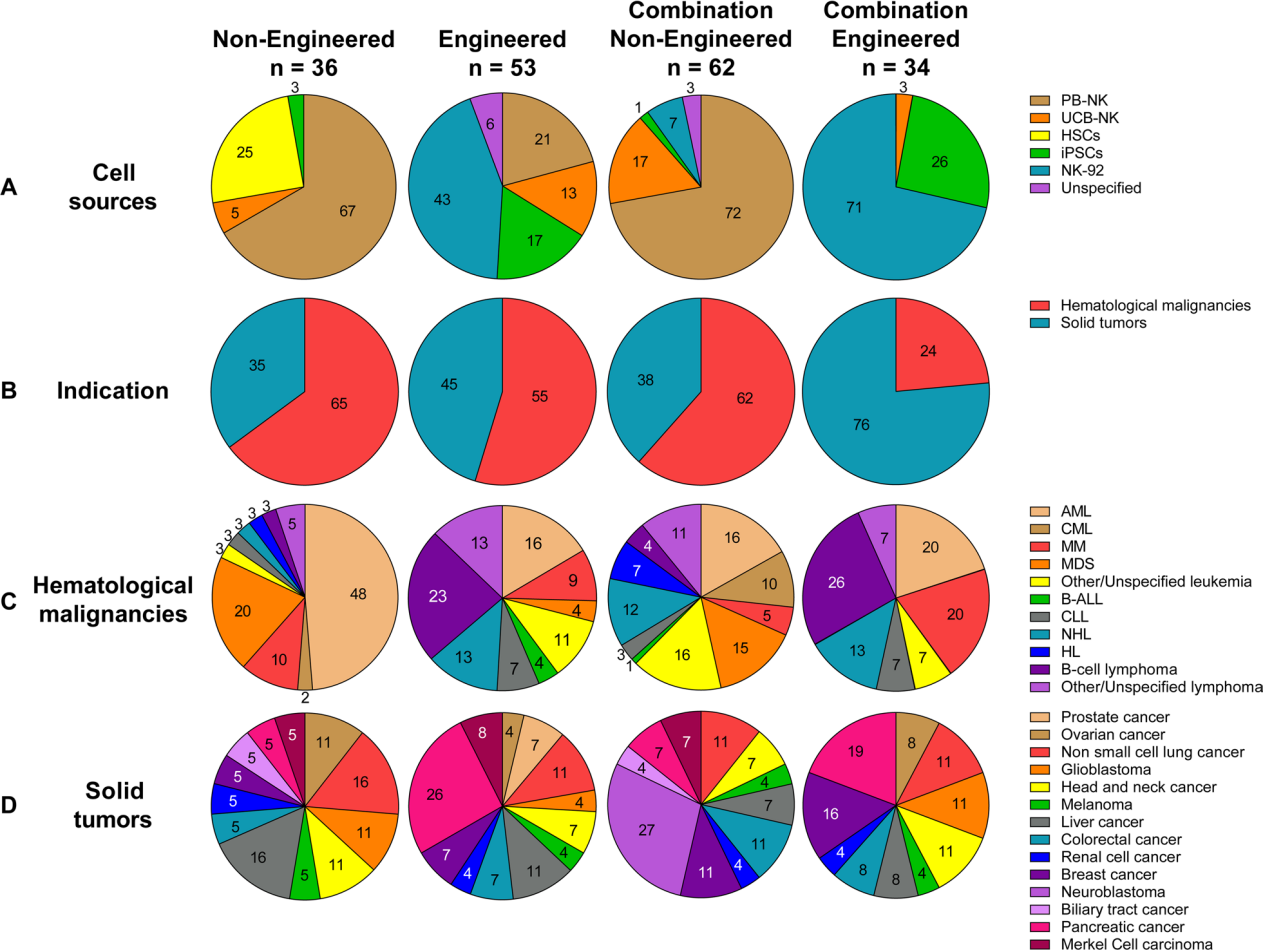
Statistics of NK cell cancer therapy with non-engineered and engineered NK cells used as monotherapy or in combination therapies. The pie charts present the major features of NK cell products reaching the clinical stage until December 2021, as reported on ClinicalTrials.gov. Data are presented for non-engineered NK cell products (based on *N* = 36 clinical trials, initiated after March 2017), engineered NK cell products (*N* = 53 trials), non-engineered (*N* = 62 trials) and engineered (*N* = 34 trials) NK cell combination therapies. For every pie, numbers indicate the percentage of the whole taken by each slice. **A** Cell sources used as starting material for the manufacturing of NK cell products, divided by category: peripheral blood-derived NK cells (PB-NK), umbilical cord blood-derived NK cells (UCB-NK), hematopoietic stem cells (HSCs), induced pluripotent stem cells (iPSCs) and NK cell lines (NK-92). Undisclosed sources are labeled as “Unspecified.” **B** Classification of the cancer indication targeted in clinical trials, divided between hematological malignancies and solid tumors. **C** and **D** Breakdown of the types of hematological malignancies (**C**) and solid tumors (**D**) indications most frequently targeted by NK cell therapeutics. Undisclosed indications are labeled as “Unspecified.” In **B**, **C** and **D**, one trial can include more than one indication. AML: acute myeloid leukemia; CML: chronic myeloid leukemia; MM: multiple myeloma; MDS: myelodysplastic syndromes; B-ALL: B cell acute lymphoblastic leukemia; CLL: chronic lymphocytic leukemia; NHL: non-Hodgkin lymphoma; HL: Hodgkin lymphoma

Clinical product development of NK cell immunotherapies is based on 3 main pillars: non-engineered NK cells, engineered NK cells, and combination therapy of non-engineered or engineered NK cells with several types of agents. A comprehensive overview of all clinical trials registered on ClinicalTrials.gov, divided by category, is presented in Additional file 1: Table S1 for non-engineered NK cells (for this category, only including trials from March 2017 onwards), in Additional file 2: Table S2 for engineered NK cells, in Additional file 3: Table S3 for non-engineered NK cell combination therapies and in Additional file 4: Table S4 for engineered NK cell combination therapies. A condensed summary is shown in the Table 1. Figure 2 presents a summary of the field, showing the main characteristics of the NK cell products that have reached the clinical stage, divided by category. The pie charts in Fig. 2A describe the different cell sources used as starting material for NK cell manufacturing, with their relative abundance. In Fig. 2B, the therapeutic focus is classified into hematological malignancies or solid tumors, to provide a high-level impression on the targeted indications per category. A breakdown of the most frequently targeted hematological malignancies and solid tumors is shown in Fig. 2C and D, respectively. The next sections will provide a detailed description of the non-engineered, engineered, and combination NK cell therapies that have reached the clinical stage, using the information collected from clinical trial protocols on ClinicalTrials.gov and from peer-reviewed clinical and preclinical studies, company reports, patents, and press releases. State-of-the-art strategies for cell sourcing, product manufacturing and formulation, engineering (if applicable) and administration to patients during clinical studies will be discussed. Due to the lack of comprehensive reports on ongoing clinical trials in such a rapidly evolving field, this may not be a fully representative overview of the final product manufacturing process. However, this review provides a map to navigate through the winding, yet the promising road to NK cell immunotherapy commercialization.

**Table 1:** Summary of the current clinical development of NK cell therapies

Sponsor	Product name	NK source	Malignancy	Culture process	Clinical product development			
					Non-engineered	Engineered	Combination Non-engineered	Combination Engineered
Acepodia	oNK cells	NK-92	HER2 + Solid Tumors	Culture with X-VIVO 10 medium, platelet lysates and IL-2 for 6 passages			NCT04319757	
Allife Medical Science and Technology	–	PB-NK	B-cell Lymphoma, Prostatic Cancer, Ovarian Cancer	No information found		NCT03690310 NCT03692767 NCT03824964 NCT03824951 NCT03692663 NCT03692637		
Altor BioScience	–	PB-NK	AML	Activation with ALT-801			NCT01478074	
Artiva Biotherapeutics	–	UCB-NK	NHL	CD3- with the eFeeder technology GC Pharma) to generate a Master Cell Bank. Expanded and cryopreserved before use			NCT04673617	
Asan Medical Center	–	PB-NK	NSCLC, Brain and Central Nervous System Tumors, AML, ALL, MM, MDS, Leukemias, Lymphomas, others	CD3-/CD56 + with α-MEM medium, IL-15, IL-21 and hydrocortisone for 13–20 days	NCT03366064		NCT00823524 NCT01795378 NCT02477787	
Asclepius Technology Company Group (Suzhou)	–	NK-92	MM, Solid Tumors, Pancreatic Cancer	No information found		NCT03940833 NCT03940820 NCT03941457 NCT03931720		
Beijing 302 Hospital	–	PB-NK	HCC	Culture for 14 days. No additional information found	NCT04162158			
Case Comprehensive Cancer Center	–	PB-NK	AML, MDS, CML, CLL, NHL, HL, CRC, Soft Tissue Sarcoma, Ewing sarcoma, Rhabdomyosarcoma	Culture with irradiated feeder cells OCI-AML3 expressing mbIL-21 (NKF cells) and IL-2 for 3 weeks			NCT02890758	
Cedars-Sinai Medical Center	–	PB-NK	MM, CLL, HL, Lymphoma	CD56 + cells. No additional information found	NCT03524235			
Celularity Incorporated	CYNK-001	UCB-CD34	MM, AML, Leukemias, GBM, Astrocytoma, Giant Cell Glioblastoma and others	Culture with TPO, SCF, Flt3-L, IL-7, IL-15 and IL-2 for 35 days	NCT04309084 NCT04310592 NCT04489420			
	PNK-007	UCB-CD34	AML, MM		NCT02781467 NCT02955550		NCT02955550	
Centre Hospitalier Universitaire Régional de Besançon	–	PB-NK	Gastrointestinal Cancers	CD3- cultured overnight in X-VIVO 15 medium with 10% autologous serum and IL-2			NCT02845999	
Children's Hospital Los Angeles	–	PB-NK	AML	Culture with IL-21 and irradiated feeder cells	NCT04836390			
Dana-Farber Cancer Institute	–	PB-NK	AML, MDS, HNSCC	Culture with RPMI-1640 medium, 10% HS, IL-12, IL-15 and IL-18	NCT04024761 NCT04290546		NCT04290546	
Deverra Therapeutics	DVX201	UCB-CD34	AML, MDS	CD34 + cultured in vessel precoated with Delta1ext-IgG (Notch ligand), serum-free medium StemSpan SFEM, IL-3, TPO, IL-6, Flt-3L, SCF for 14–16 days to generate progenitor cells. Further differentiation process not disclosed	NCT04901416			
Duke University	NK-DLI	PB-NK	Myeloid and Lymphoid Malignancies	Enriched CD56 +			NCT02452697	
Fate Therapeutics	FATE-NK100	PB-NK	EGFR1 + Solid Tumors, HER2 + Gastric and Breast Cancer, Solid Tumors	No information found			NCT03319459	
	FT500	iPSCs	Solid Tumors, Lymphomas	iPSCs transduced and differentiated in CD34 + hnCD16 + (18–21 days), CD34 differentiation in B0 medium with 20% HS, IL-3, IL-7, IL-15, SCF, Flt3L on EL08-1D2 stroma (culture days 28–35). NK-cells expanded using irradiated K562-mbIL21-41BBL cells and IL-2	NCT03841110		NCT03841110	
	FT596	iPSCs	B-cell Lymphoma, CLL			NCT04245722		NCT04245722
	FT516	iPSCs	B-cell Lymphoma, AML, Solid Tumors			NCT04023071 NCT04551885		NCT04023071 NCT04551885
	FT538	iPSCs	AML, MM, Solid Tumors			NCT04614636 NCT05069935		NCT04614636 NCT05069935
	FT576	iPSCs	MM			NCT05182073		NCT05182073
Fondazione Policlinico Universitario Agostino Gemelli IRCCS	–	PB-NK	AML, MDS	Unstimulated and not expanded	NCT04166929		NCT04166929	
Fred Hutchinson Cancer Research Center	–	PB-NK	Leukemia, MDS, other Hematological Malignancies	No information found			NCT00789776 NCT00450983	
Fuda Cancer Hospital	HANK	PB-NK	Liver Carcinoma, B-cell Lymphoma, Breast Cancer, NSCLC, Solid Tumors	*Culture with Human HANK Cell In Vitro Preparation and Culture Kit, serum free medium, additives (like IL-2) and irradiated K562-mbIL15-41BBL feeder layer for 12 days ^Cultured with RPMI-1640 medium, 10% FCS, IL-2 and K562-mbIL15-41BBL irradiated feeder cells for 2–3 weeks	*NCT03008343		*NCT02843061 *NCT02843126 ^NCT02845856 *NCT02843204 *NCT02857920	
GC Pharma	MG4101	PB-NK	NHL	CD3-with CellGro SCGM serum free medium, 1% auto-plasma, anti-CD3 (OKT3), IL-2 and irradiated autologous PBMC for 14 days			NCT03778619	
Glycostem Therapeutics	GTA002	UCB-CD34	AML	CD34 + with GBGM medium with 2–10% serum, GM-CSF, G-SCF, IL-6, SCF, Flt3L, TPO, IL-7, IL-2 and IL-15 for 42 days	NCT04632316			
ImmunityBio	CD19 t-haNK	NK-92	DLBCL	Culture with phenol-red free and gentamycin-free X-VIVO 10 medium, 5% HS and irradiated prior to infusion		NCT04052061		
	PD-L1 t-haNK	NK-92	Solid Tumors, TNBC, Pancreatic Cancer, NSCLC, SCLC, HNSCC, RCC, CRC, Urothelial Carcinoma, MCC, Melanoma, Gastric Cancer, Cervical Cancer, HCC, MSI-H and dMMR Solid Tumors			NCT04050709 NCT04927884 NCT04390399 NCT03228667		NCT04927884 NCT04390399 NCT03228667
	haNK	NK-92	MCC, HCC, Pancreatic Cancer, SCC, CRC, TNBC, Ovarian Cancer, NSCLC, Chordoma, Malignant Neoplasm, Urothelial Carcinoma, HNSCC, NHL, MCC			NCT03853317 NCT03563170 NCT03329248 NCT03387098 NCT03586869 NCT03563144 NCT03387111		NCT03853317 NCT03563170 NCT03329248 NCT03387098 NCT03586869 NCT03563144 NCT03387111 NCT03563157 NCT03554109 NCT03387085 NCT03197584 NCT03169777 NCT03175666 NCT03169738 NCT03574649 NCT03647423 NCT03197571 NCT03169764 NCT03169790 NCT03167164
	aNK	NK-92	MCC, Pancreatic Cancer				NCT02465957 NCT03136406	
IRCCS Azienda Ospedaliero-Universitaria di Bologna	–	PB-NK	AML	Culture with IL-2 and feeder cells derived from 5–9 donors	NCT03955848			
Johann Wolfgang Goethe University Hospital	NK-92/5.28.z or HER2.taNK	NK-92	GBM	NK-92/5.28.z with X-VIVO 10 medium, 5% heat inactivated human plasma and IL-2. Y-irradiation with 10 Gy prior to infusion		NCT03383978		
Kiadis Pharma	K-NK002	PB-NK	AML, MDS	CD3- with feeder-free with PM21 particles for 13 days	NCT04395092		NCT04395092	
	KDS-1001	PB-NK	CML				NCT04808115	
Masonic Cancer Center, University of Minnesota	GDA-201	PB-NK	MM, NHL, Lymphomas	CD3- with NAM and IL-15 (additional cytokines not disclosed), HS, feeder free for 14–16 days	NCT03019666		NCT03019666	
	FATE-NK100	PB-NK	Epithelial Ovarian Cancer, Fallopian Tube Cancer and Primary Peritoneal Cancer, AML	CD3-/CD19- or CD3-/CD56 + in B0 mediawith 20% HS, IL-15, CHIR99021 GSK3 inhibitor and no feeder cells for 7 days	NCT03213964 NCT03081780			
	FT596	iPSCs	NHL, DLBCL, High-grade B-cell Lymphoma	iPSCs transduced and differentiated in CD34 + hnCD16 + (18–21 days), CD34 differentiation in B0 medium with 20% HS, IL-3, IL-7, IL-15, SCF, Flt3L on EL08-1D2 stroma (culture days 28–35). NK-cells expanded using irradiated K562-mbIL21-41BBL cells and IL-2		NCT04555811		NCT04555811
	FT516	iPSCs	Ovarian Cancer, Fallopian Tube Adenocarcinoma, Primary Peritoneal Cavity Cancer			NCT04630769		NCT04630769
	FT538	iPSCs	AML, Myeloid Leukemia and Monocytic Leukemia			NCT04714372		NCT04714372
	–	PB-NK	AML	CD3-/CD19-/CD56 + cultured overnight with IL-2			NCT01106950	
	–	PB-NK	NHL or CLL	CD3- cultured with IL-2 overnight			NCT00625729	
	–	PB-NK	NHL, CLL	CD3-/CD19-cultured in X-VIVO 15 with 10% HS and IL-2			NCT01181258	
	–	PB-NK	AML	CD3-/CD19- cultured overnight with ALT-803			NCT03050216	
	–	UCB-NK	Leukemia, MDS	No information found			NCT00354172	
MD Anderson Cancer Center	K-NK003	PB-NK	AML, MDS, CML	CD3- with feeder-free with PM21 particles for 13 days	NCT05115630		NCT05115630	
	–	PB-NK or UCB-NK	Myeloid Malignancies	Ex vivo expansion. No additional information found			NCT01823198	
	–	PB-NK	AML, MDS, CML	CD3- with FC21 in RPMI-1640 medium with IL-2 for 14 days			NCT01904136	
	–	PB-NK	Pediatric Leukemia, Lymphoma, Leukemia	No information found			NCT00941928 NCT00383994	
	–	UCB-NK	Solid Tumors, B-cell Lymphoma, MDS, AML, CRC, MM, B-cell NHL, Hematological Malignances	*Culture with 45% RPMI-1640 and 45% Click’s media with 10% HS, IL-2 and IL-21 expressing-K562 (aAPC) feeder cells (clone9.mbIL21) for 14 days. CD3 depletion on day 7 before plating the cells again ^Transduced on day 6 and harvested on day 15	*NCT03420963	^NCT03056339 NCT03579927 NCT05110742 NCT05092451	NCT05040568 NCT02280525 NCT01619761 NCT03019640 NCT01729091	NCT03579927
	AFM13-NK	UCB-NK	HL, NHL	CD56 + with IL-2 and irradiated K562 (clone 9.mbIL21) feeder cells			NCT04074746	
	–	NK-92	MDS, Leukemia, Lymphoma, MM	No information found			NCT02727803	
Medical College of Wisconsin	–	PB-NK	Sarcomas	No information found			NCT02100891	
Memorial Sloan Kettering Cancer Center	–	PB-NK	Neuroblastoma	CD3-/CD56 + cultured with IL-2 overnight			NCT00877110 NCT02650648	
Miltenyi Biotech	–	PB-NK	AML	CD3-/CD19- or CD3-/CD56 + . No additional information found	NCT03152526			
Nanfang Hospital of Southern Medical University	–	UCB-NK	HCC	No information found			NCT05171309	
National Cancer Institute	PD-L1 t-haNK	NK-92	GEJ Cancers, HNSCC	Culture with phenol-red free and gentamycin-free X-VIVO 10 medium, 5% HS and co-irradiated prior to infusion		NCT04847466		NCT04847466
	–	PB-NK	Leukemia, Lymphoma	Culture with K562-mbIL15-41BBL and IL-15			NCT01287104	
National University Health System Singapore	–	PB-NK	B-ALL, Neuroblastoma	Cultured with K562-mb15-4-1BBL *(with IL-2 and then transduced)		*NCT01974479	NCT03242603	
Nkarta Therapeutics	NKX101-101	PB-NK	AML, MDS	Culture with stimulatory cells, transduced and expanded with mbIL-15		NCT04623944		
	NKX019	PB-NK	NHL, CLL, B-ALL			NCT05020678		
Ohio State University Comprehensive Cancer Center	K-NK003	PB-NK	AML, MDS	CD3- with FC21 mbIL21 feeder cells for 13 days	NCT04220684			
	–	PB-NK	T cell Leukemia/Lymphoma	Culture with NK cell expansion medium, IL-2 from day 3, irradiated K562 Cl9 mIL21 (CSTX002) feeder cells for 21 days			NCT04848064	
PersonGen BioTherapeutics	–	NK-92	Solid Tumors, AML, Leukemias, Lymphomas,	No information found		NCT02839954 NCT02944162 NCT02742727		
	PCAR-119	NK-92	B-cell Lymphoma, Leukemia	No information found		NCT02892695		
Precision Biotech Taiwan Corp	PB103	PB-NK	NSCLC	Culture with DMEM-F12, ultraGRO, RPMI-1640 or SCGM media, without serum, with IL-2, heparin, anti-CD3 mAb for 10–16 days	NCT04616209			
Radboud University	–	UCB-CD34	AML, Ovarian Cancer, Fallopian tube or Primary Peritoneal Carcinoma	CD34 + culture process includes StemRegenin 1, GM-CSF, G-SCF, IL-6, SCF, Flt3L, TPO, IL-7, IL-2 and IL-15, 10% HS for 42 days	NCT04347616 NCT03539406			
Royan Institute	–	PB-NK	GBM	Culture with RPMI-1640 medium, IL-2 and HSP70 for 16 days	NCT05108012			
Samsung Medical center	–	PB-NK	Neuroblastoma, Soft Tissue Sarcomas and Osteosarcomas	No information found			NCT01807468	
Seoul National University Hospital	MG4101	PB-NK	AML	CD3- with CellGro SCGM serum free medium, 1% auto-plasma, anti-CD3 (OKT3), IL-2 and irradiated autologous PBMCs for 14 days	NCT03349502			
Shandong Golden Brick Biotechnology	–	UCB-NK	Advanced Gastric Cancer	No information found	NCT04385641			
Shanghai East Hospital	–	Unknown	Solid Tumors	No information found		NCT05137275		
Shanghai iCELL Biotechnology		PB-NK	AML	No information found	NCT04209712			
SMT bio	SMT-NK	PB-NK	BTC	No information found			NCT03937895	
St. Jude Children's Research Hospital	–	PB-NK	B-ALL, Leukemia, Lymphoma	*CD3- hematopoietic progenitor cells CD34 + CD45RA-		NCT00995137	*NCT01807611 NCT01621477 NCT01576692	
	–	PB-NK	Pediatric AML, ALL, CML, MDS, Histiocytosis, Neuroblastoma, Lymphoma, High-risk Tumors	CD3-/CD56 + infused fresh *(less than 12 h process)			*NCT00145626 *NCT02130869	
	–	PB-NK	Pediatric Neuroblastoma				NCT01857934	
Takeda	TAK-007	UCB-NK	B-cell NHL, NHL	No information found		NCT05020015		
The Third Affiliated Hospital of Guangzhou Medical University	–	PB-NK	Metastatic Solid Tumors	Culture with NK cell growth medium with 1% HS and IL-2		NCT03415100		
University Hospital, Basel, Switzerland	–	PB-NK	AML, MDS	CD3-/CD56 + with SCGM medium, 5% HS, IL-2, IL-15, fresh anti-CD3 every day and irradiated autologous feeder cells for 19–20 days	NCT03300492			
University of Arkansas	–	PB-NK	MM	Culture with K562 mIL15/4-1BBL			NCT01313897	
University of Wisconsin	EANK	PB-NK	Neuroblastoma, Osteosarcoma	Culture with K562-mbIL15-41BBL			NCT03209869	
Washington University School of Medicine	WU-NK-101	PB-NK	AML, MDS	Culture with RPMI-1640 medium, 10% HS, no feeder cells for 14 days. Activation with heteromeric fusion protein complex IL-12, IL-15, IL-18 (WU-PRIME) for 12-16 h	NCT04893915			
	–	PB-NK	AML, MDS	*CD3-/CD56 + with IL-2, IL-15 and IL-18 for 12 h	NCT04354025		*NCT01898793	
Wuhan Union Hospital	–	UCB-NK	NHL, CLL	No information found		NCT04796675		
Xinqiao Hospital of Chongqing		UCB-NK	MM	No information found		NCT05008536		
	–	Unknown	NHL, AML	No information found		NCT04639739 NCT05008575		
Xinxiang Medical University	–	NK-92	NSCLC	No information found		NCT03656705		
Yonsei University	–	PB-NK	BTC	ALyS505NK-IL2 for 21 days	NCT03358849			

Allogeneic NK cell therapies currently under clinical development are presented, arranged by sponsor of the clinical study. Data is collected from *N* = 36 clinical trials with non-engineered, *N* = 53 with engineered, *N* = 62 with non-engineered combination and *N* = 34 with engineered combination allogeneic NK cell therapies, registered on ClinicalTrials.gov between March 2017 and December 2021 (for non-engineered monotherapies), or until 31–12-2021 (for the other categories). Product name, NK cell source, treated malignancy, cell culture process (when available) and type of clinical product development (Non-engineered, Engineered, Combination Non-engineered, or Combination Engineered) are described. Trial status is updated to August 2022

4-1BBL: 4-1BB ligand; aAPC: Artificial antigen presenting cells; AML: Acute Myeloid Leukemia; B-ALL: B cell—Acute Lymphoblastic Leukemia; BTC: Biliary Tract Cancer; CLL: Chronic Lymphocytic Leukemia; CML: Chronic Myeloid Leukemia; CRC: Colorectal Cancer; DLBCL: Diffuse Large B-cell Lymphoma; DMEM: Dulbecco's Modified Eagle's Medium; dMMR: Mismatch Repair Deficiency; EGFR: Epidermal Growth Factor Receptor; FC21: Feeder Cells 21; FCS: Fetal Calf Serum; FL: Follicular Lymphoma; Flt-3L: FMS-like tyrosine kinase 3 ligand; GBM: Glioblastoma Multiforme; G-CSF: granulocyte colony stimulating factor; GEJ: Gastroesophageal Junction; GM-CSF: Granulocyte–Macrophage Colony-Stimulating Factor; GSK3: Glycogen synthase kinase-3; Gy: Gray; HCC: Hepatocellular Carcinoma; HER2: Human Epidermal Growth Factor Receptor 2; HL: Hodgkin Lymphoma; HNC: Head and Neck Cancer; hnCD16: high affinity non-cleavable CD16; HNSCC: Head and Neck Squamous Cell Carcinoma; HS: Human Serum; HSP70: Heat Shock Protein 70; IL-12: Interleukin-12; IL-15: Interleukin-15; IL-15Rα: Interleukin-15 receptor α; IL-18: Interleukin-18; IL-2: Interleukin-2; IL-21: Interleukin-21; IL-3: Interleukin-3; IL-6: Interleukin-6; IL-7: Interleukin-7; iPSCs: induced Pluripotent Stem Cells; mbIL-15: Membrane bound Interleukin-15; mbIL-21: Membrane bound Interleukin-21; MCC: Merkel Cell Carcinoma; MDS: Myelodysplastic Syndrome; MM: Multiple Myeloma; MSI-H: Microsatellite Instability; NHL: Non-Hodgkin Lymphoma; NK cells: Natural Killer cells; NSCLC: Non-small Cell Lung Cancer; PBMC: Peripheral Blood Mononuclear Cells; PB-NK: Peripheral blood NK cells; PM21: Plasma Membrane 21; RCC: Renal Cell Cancer; RPMI-1640: Roswell Park Memorial Institute Medium; SCC: Squamous Cell Carcinoma; SCF: Stem Cell Factor; SCGM: Stem Cell Growth Medium; SCLC: Small Cell Lung Cancer; TNBC: Triple Negative Breast Cancer; TPO: Thrombopoietin; UCB-CD34: Umbilical cord blood CD34 + cells; UCB-NK: Umbilical cord blood NK cells; α-MEM: α-Minimum Essential Medium

## Clinical manufacturing of NK cell therapies

**Fig. 3:**
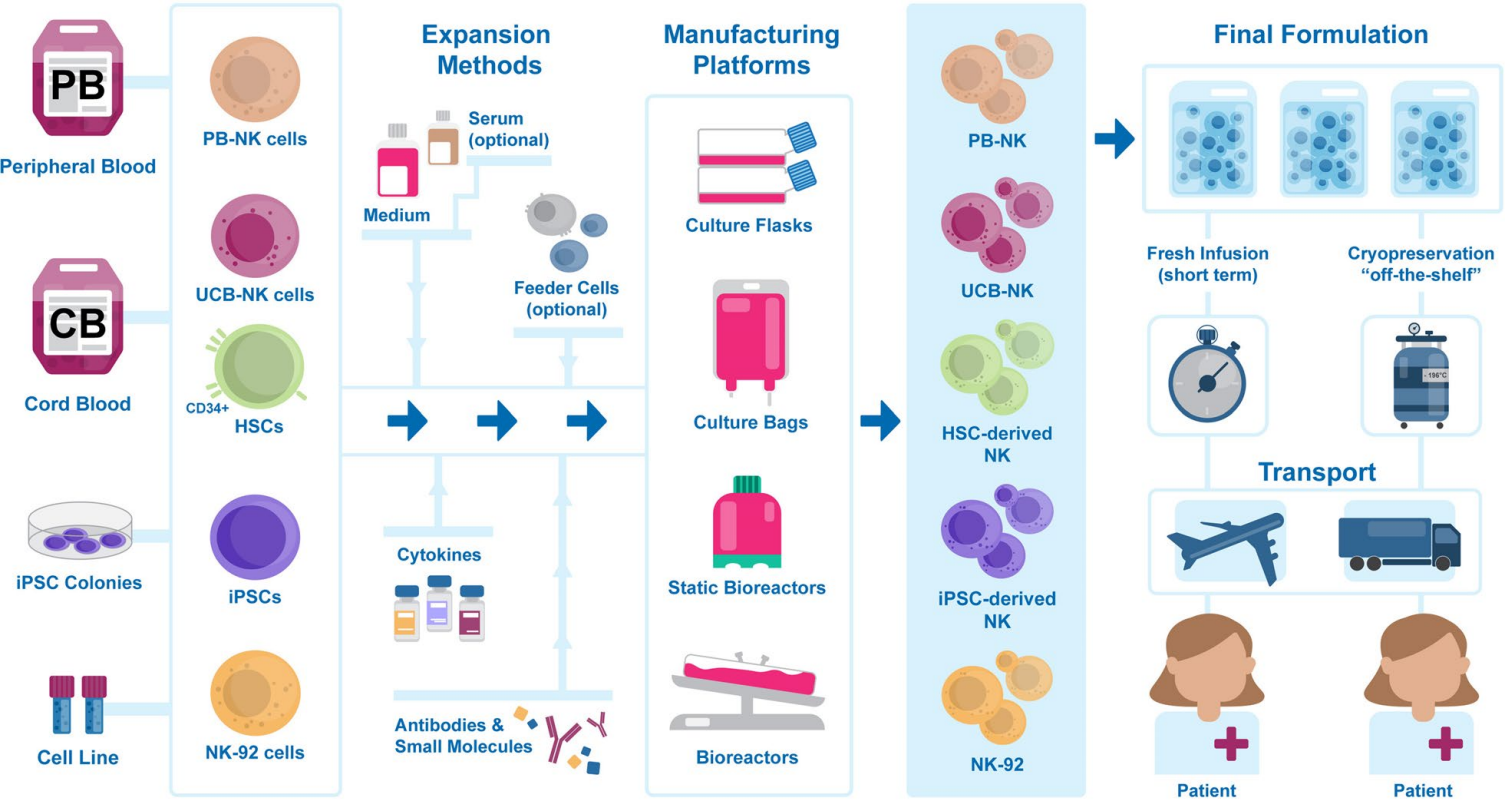
Natural killer cell therapy manufacturing process pipeline. From left to right: NK cells are isolated from peripheral blood (PB-NK), from umbilical cord blood (UCB-NK), derived from cord blood CD34-positive hematopoietic stem cells (HSCs), from induced hematopoietic stem cells (iPSCs), or from in vitro propagated cell lines (NK-92). Cell expansion (and differentiation) is achieved ex vivo by culture in suitable medium, optionally supplemented with serum, or in presence of feeder cells. Cytokines, antibodies, and other small molecules are added to support cell expansion and maturation. Manufacturing platforms include cell culture flasks, bags, and static and dynamic bioreactors. Once the appropriate number of cells with the desired phenotype is obtained, NK cells are harvested and collected in the final formulation before infusion into patients. Fresh products must be transported to the patient and administered shortly after collection. Cryopreserved products can be stored in appropriate conditions and be delivered to the patient as needed, for on-site thawing and true “off-the-shelf” administration

Successful development of allogeneic off-the-shelf NK cell therapies requires the establishment of an adequate manufacturing workflow. Such a multi-step process, as described in Fig. 3, starts with cell isolation from the starting material of choice, followed by ex vivo expansion (and differentiation) into large quantities of functional NK cells. After harvest, the final product is formulated in the appropriate medium and either immediately distributed to treatment locations to be directly administered to patients or cryopreserved before shipment for use after on-site thawing. A description of the different solutions currently adopted for each step by clinical-grade NK manufacturers is provided in the following sections.

### NK cell sources

A critical challenge faced by NK cell manufacturers is the need to generate high numbers of cells, as therapeutic doses range between 5 × 10^6^ and 1 × 10^8^ NK cells per kilogram of body weight [23]. The choice of starting material is crucial for process and product development, as source-specific manufacturing strategies must be designed according to the cells’ phenotype and expansion potential. The current clinical-stage cell sources, shown in Fig. 3, are peripheral blood NK cells (PB-NK), umbilical cord blood NK cells (UCB-NK) and hematopoietic stem cells (HSCs), induced pluripotent stem cells (iPSCs) and NK cell lines such as NK-92. The origin and characteristics of each source and the challenges they present are described below.

#### PB-NK and UCB-NK

Mature NK cells can be isolated from apheresis of peripheral blood (PB-NK cells) or from umbilical cord blood (UCB-NK cells) using cell sorting techniques or immunomagnetic cell separation platforms. Enrichment of abundant and pure NK cells is necessary to improve subsequent expansion and to minimize impurity content in the final product, especially relevant to ensure patient safety by avoiding residual allogeneic T cells which could cause GvHD. STEMCELL™ Technologies’ RosetteSep™ Immunodensity Cell Separation products are based on the incubation of raw material with antibody cocktails, followed by separation on a density gradient medium. This platform, although not good manufacturing practice (GMP)-compliant, is used to isolate cells for clinical use (Additional file 1: Table S1, row 9 [24]; Additional file 3: Table S3, rows 16, 17, 21 [25]). GMP-suitable, more complex technologies involve the use of automated cell separation systems, such as the CliniMACS® Plus (used in Additional file 1: Table S1, row 25 [26]; Additional file 3: Table S3, rows 1, 3, 40, 41 [27]) and CliniMACS Prodigy® (Additional file 1: Table S1, rows 4–6; Additional file 3: Table S3, rows 22, 23, 60 [28]) from Miltenyi Biotech. Immunomagnetic selection from blood cell pools requires cell labeling with magnetic beads coated with antibodies against the target molecules, and is used in two manners by NK cell manufacturers: for the enrichment of CD56-expressing NK cells (positive selection, e.g., Additional file 1: Table S1, row 11 and Additional file 3: Table S3, row 47 [29]), or to deplete CD3/CD19-expressing T and B cells (negative selection, e.g., Additional file 1: Table S1, row 12 [30]). A combination of CD3^+^ cell depletion followed by CD56^+^ cell enrichment is also possible (Additional file 1: Table S1, row 15 [31]). Interestingly, Gamida Cell described the advantage of CD3/CD19 depletion (reaching on average 65-fold expansion) over CD56 enrichment (limited to tenfold). Higher expansion after negative selection is supported by myeloid cells, which disappear after the first days of culture [30].

Peripheral blood is a valuable and accessible source of mature NK cells; although they represent a small percentage of circulating lymphocytes (5–15%) [32], the majority of the non-genetically modified NK cell products that are currently under clinical investigation are manufactured using peripheral blood as starting material, both in monotherapy and in combination with other therapeutic agents (Fig. 2A). Products generated from PB-NK cells are reported in Additional file 1: Table S1, Additional file 2: Table S2, Additional file 3: Table S3 and Additional file 4: Table S4; examples include K-NK002 and K-NK003 from Kiadis Pharma (Additional file 1: Table S1, rows 4–6; Additional file 3: Table S3, row 22), GDA-201 from Gamida Cell (Additional file 1: Table S1, row 12), MG4101 of GC Pharma (Additional file 1: Table S1, row 14; Additional file 3: Table S3, row 29), FATE-NK100 from Fate Therapeutics (Additional file 1: Table S1, rows 21–22; Additional file 3: Table S3, row 36) and HANK cells from Fuda Cancer Hospital (Additional file 1: Table S1, row 24 and Additional file 3: Table S3, rows 31–35).

As an alternative to peripheral blood, umbilical cord blood is available off-the-shelf through banks and contains a high percentage of NK cells (15–30% of total lymphocytes), though the total number per unit is lower due to the smaller sampling volume [33, 34]. Notably, UCB-NK cells differ from PB-NK cells, showing a more immature phenotype represented by a subset of CD56^−^/CD16^+^ NK cells [35], have overall high expression of NKG2A and low levels of KIR molecules [36] and lower expression of adhesion molecules such as CD2, CD11a, CD18 and CD226 [37]. The use of UCB-NK cells is reported for the development of non-engineered and engineered NK cell therapies, with the widest application as non-engineered combinations (Fig. 2A). UCB-NK cells are chosen by several manufacturers (see Additional file 1: Table S1, Additional file 2: Table S2, Additional file 3: Table S3 and Additional file 4: Table S4), for example the MD Anderson Cancer Center for their non-engineered (Additional file 1: Table S1, row 34), engineered (Additional file 2: Table S2, rows 12–15), and combination therapy (Additional file 3: Table S3, rows 24, 48) products, and Artiva Biotherapeutics for the development of combination therapies (Additional file 3: Table S3, row 43).

#### *UCB- and placenta-derived CD34*^+^*HSCs*

Umbilical cord and placenta blood CD34-expressing HSCs can be expanded and differentiated ex vivo into mature NK cells. Stem cell isolation is performed by immunomagnetic positive selection, in a similar manner as described for leukapheresis products by targeting the CD34 surface antigen, e.g., using the CliniMACS (Deverra Therapeutics, Additional file 1: Table S1, row 25 [26] and Radboud University, Additional file 2: Table S2, rows 26 and 33 [38]) or the CliniMACS Prodigy® (Glycostem Therapeutics, Additional file 1: Table S1, row 27 [39]) platforms.

As explained by Luevano et al., NK cell generation from CD34^+^ HSCs could overcome the risk of exhaustion of more mature PB-NK and UCB-NK cells during prolonged exposure to cytokines in expansion protocols [40]. Although bone marrow and granulocyte colony-stimulating factor (G-CSF)-mobilized peripheral blood are suitable sources for HSCs, cord blood-derived CD34^+^ stem cells might be preferable, as a less stringent antigen matching resulted in lower immunogenicity and therefore reduced risk of GvHD and relapse after UCB-hematopoietic stem cell transplant (HSCT) [41–43]. Currently, umbilical cord blood and placenta blood are the only HSC sources that have reached clinical-stage applications as starting material for NK cell manufacturing (Fig. 2A). NK cell generation from HSCs is achieved with specific protocols, which include expansion and differentiation of NK cell progenitors into mature NK cells. The first successful clinical results were reported by Dolstra et al. [44], from a Phase I trial using NK cells derived from UCB-CD34^+^ progenitor cells, initiated and manufactured by Glycostem Therapeutics, facilitated and performed at the Radboud University Medical Centre in Nijmegen. Glycostem Therapeutics has developed a large-scale GMP-compliant expansion and differentiation process of UCB-CD34^+^ cells into GTA002 NK cells (Additional file 1: Table S1, row 27) [39] based on a three-step culture scheme [45]. Radboud University is using a similar technology in their most recently announced trials (Additional file 1: Table S1, row 26 and 33 [38]). In addition to Glycostem's pioneering work, Celularity has developed Taniraleucel, which is the approved international nonproprietary name (INN) for their cryopreserved CD34^+^ umbilical cord blood-derived NK cell product [46]. Recently, they disclosed that their manufacturing system is based on the Glycostem process published in 2010 by Spanholtz et al. [47, 48] and that Taniraleucel (CYNK-001) is the cryopreserved successor of the previous fresh UCB-CD34 product, PNK-007 (Additional file 1: Table S1, rows 30–31) [49]. In 2020, two follow-up studies with CYNK-001 in leukemia and myeloma were registered on ClinicalTrials.gov (Additional file 1: Table S1, rows 28 and 29), in addition to a trial in glioblastoma (Additional file 1: Table S1, row 32). The use of UCB-CD34^+^ cells as a source for NK cell therapies is also explored by Deverra Therapeutics, with their non-engineered product DVX201 (Additional file 1: Table S1, row 25).

#### NK cell lines

Due to the challenges associated with primary allogeneic NK cells (cell purification, ex vivo expansion and differentiation, donor-related variability, resistance to genetic modifications), investigators started exploring the use of stable NK cell lines. As exhaustively reviewed by Fabian et al., these immortalized cell lines are homogenous NK cell populations, which can be easily maintained and expanded in culture to serve as a steady supply of off-the-shelf NK cell products, thanks to their clonal expansion capacity [50]. Currently, 10 different NK cell lines have been established: NK-92 [51], YT or YT-S [52], KHYG-1 [53], NK3.3 [54], NK-YS [55], NKL [56], NKG [57], SNK-6 [58], and IMC-1 [59]; however, only NK-92 has entered clinical product development [60]. Established in 1992 from a 50-year-old male patient with malignant non-Hodgkin’s lymphoma (NHL), NK-92 cells rely on the presence of recombinant human IL-2 for growth [51] and express several cell surface antigens such as CD56, CD2, CD45, CD28, activating receptors such as NKp30, NKp44, NKp46, NKG2D, and 2B4. They are devoid of CD3, CD4, CD8, CD19, CD34, HLA-DR and of most inhibitory receptors such as TIGIT and PD-1, except for CD94-NKG2A and low levels of KIR2DL4 [61]. Despite their inability to exert antibody-dependent cellular cytotoxicity (ADCC) due to a lack of CD16 expression, NK-92 cells have prominent levels of granzyme B and perforin [51]. Although promising, NK cell lines raise concerns regarding their tumorigenicity and must be irradiated before infusion into patients to avoid undesired clonal expansion, but such treatment can affect their in vivo persistence and efficacy [62]. The pioneer Phase I study from Arai et al. first showed the feasibility and the safety of NK-92 cell administration in cancer patients [63]. Today, NK-92 cells are mostly investigated as engineered NK therapies, alone or in combination with other agents (Fig. 2A). The major NK-92 product manufacturers are Immunity Bio (Additional file 2: Table S2, rows 38–42, 44–50; Additional file 3: Table S3, rows 5 and 52; Additional file 4: Table S4, rows 12–34), PersonGen BioTherapeutics (Additional file 2: Table S2, rows 28, 30, 32, 33), Asclepius Technology Company Group (Additional file 2: Table S2, rows 31, 34, 36, 37) and Acepodia (Additional file 3: Table S3, row 27).

#### iPSCs

Induced pluripotent stem cell-derived NK cells (iPSC-NK cells) are one of the most recent additions to the landscape of NK cell-based therapies, providing a new perspective for the generation of potentially unlimited and single-donor-derived NK cell products [64] through master cell banks. Originating from reprogrammed somatic cells such as fibroblasts and blood cells, iPSCs are easily accessible and their pluripotent phenotype offers high expansion and differentiation capacity to NK cells. iPSC master cell banks provide a continuous supply of genetically identical donor material [65] and offer opportunities for product standardization and process robustness [66]. iPSC-NK cell therapies are explored in all categories, but most prominently as engineered NK therapies, especially in combination (Fig. 2A). Fate Therapeutics leads the field, with five products currently the clinic: one non-engineered, FT500 (Additional file 1: Table S1, row 36; Additional file 3: Table S3, row 37), and 4 engineered iPSC-NK, FT516, FT538, FT596 and FT576 (Additional file 2: Table S2, rows 19–27; Additional file 4: Table S4, rows 1–9), currently evaluated in 10 Phase I trials against various hematological and solid malignancies.

### NK cell expansion and activation methods

With the exception of a few clinical trials (Additional file 1: Table S1, row 8; Additional file 3: Table S3, rows 49 and 50 [67]), NK cell therapies undergo ex vivo expansion and activation processes before administration. Expansion protocols generally last 2–3 weeks if starting with NK cells but can be longer for HSCs-NK and iPSC-NK, as they require expansion and differentiation. The NK expansion approaches currently used to provide products for clinical trials, including ex vivo culture formulations, tools, and platforms, are summarized in Fig. 3 and described below.

#### Cell culture medium

Cell culture media are complex mixtures of many substances able to support cells’ fitness and growth, such as nutrients, salts, proteins, vitamins, buffers, hormones, and trace elements. Several GMP-compliant media for ex vivo NK cell cultures are commercially available and are used to generate cells for clinical trials. Examples include RPMI-1640 (e.g., Additional file 1: Table S1, rows 2 [68], 9, 17 [24]), CellGro Stem cell growth medium, SCGM from CellGenix (e.g., Additional file 1: Table S1, rows 14–15; Additional file 3: Table S3, row 29 [69]), NK MACS® from Miltenyi [70], α-MEM (Additional file 3: Table S3, rows 16, 17, 21 [25]) and X-VIVO™ 10 (e.g., NK-92 cell products in Additional file 2: Table S2, rows 38–50; Additional file 4: Table S4, rows 10, 12–34 [71]). According to GMP requirements for raw materials, the cell culture medium formulation used in the manufacturing of cell therapeutics must be defined, approved, and regularly verified [72]. As the formulation of commercially available media is often not (completely) disclosed, fulfillment of such requirements might be at risk. As a solution, some NK manufacturers have developed customized media formulations which grant them the advantage of a defined medium composition, as well as more control over medium supply. One such example is Glycostem Therapeutics, which has formulated the GMP-grade Glycostem basal growth medium (GBGM) for the culture of HSC-derived NK cells (Additional file 1: Table S1, row 27). In a previous study from 2010, this medium has been shown to facilitate ex vivo generation of NK cells from UCB-CD34^+^ cells with more than 4-log expansion [73].

#### Serum

While the culture medium formulation varies between different manufacturing processes, the addition of serum, providing hormones, vitamins, transport proteins, and growth factors for optimal NK cell maintenance, is a necessity for most products [74]. The use of fetal bovine serum (FBS) [75] is associated with safety concerns. Conversely, human-derived AB serum (HS) isolated from healthy individuals in certified blood donation centers represents a well-recognized, safe, clinically tested, and high-quality option [76]. Human serum is generally added as 1–10% of the total culture volume, with some exceptions (Additional file 1: Table S1, rows 21–22 [77]; Additional file 1: Table S1, row 14 and Additional file 3: Table S3, row 29 [69]). Despite the benefits, the limited amount of serum available from banks is hardly compatible with the rapidly growing cell therapy industry where the demand might rapidly exceed the supply. Serum batch-to-batch variability, especially if combined with donor-derived starting material, affects product reproducibility and process standardization thus challenging critical aspects such as raw material quality assurance. Additionally, the potential risk of viral transmission is a major concern from regulatory authorities [78]. Although strict measures, such as donor selection criteria, virus screening, and inactivation, are taken to minimize contamination, regulatory agencies promote the use of serum substitutes or of serum-free media for the manufacturing of cell-based medicinal products [79, 80].

Currently, only a few serum replacement strategies have successfully reached clinical NK cell manufacturing. Platelet lysates are used to support NK-92-derived oNK cells from Acepodia (Additional file 3: Table S3, row 27), which are cultured in X-VIVO™ 10 medium with IL-2 for 6 passages. Precision Biotechnology's PB103 cells (Additional file 1: Table S1, row 18) are expanded between 3000 and 7000-fold when PB-NKs are cultured for 18 days in serum-free cell culture medium with the addition of cytokines and supplements (IL-2, anti-CD3, and heparin) [81]; additional serum replacements have not been disclosed. Interestingly, the HANK Cell In Vitro Preparation and Culture Kit, developed by HANK Bioengineering, supports the generation of Highly Activated NK cells (HANK cells) (Additional file 1: Table S1, row 24; Additional file 3: Table S3, rows 31–32, 34–35) from PB-NKs cultured in serum-free medium for 12 days [82]. Unfortunately, the kit components are not disclosed. This process can generate approximately 8–10 billion HANK cells from a starting material of 80 mL of peripheral blood [83]. Moreover, the use of StemSpan™ SFEM serum-free expansion medium for the manufacturing of DVX201 NK cells from umbilical cord blood HSCs is reported by Deverra Therapeutics (Additional file 1: Table S1, row 25 [84]).

#### Cytokines and other supplements

Cytokines have pivotal roles in the maturation, activation, survival, and functionality of NK cells [21] and therefore are fundamental components of ex vivo NK cell maintenance systems. Cytokines are employed for the expansion and activation of isolated NK cells (PB-NK and UCB-NK), for the maintenance of NK-92 cells, and for the expansion and differentiation of CD34^+^ HSCs or iPSCs into NK cells.

Different forms of cytokines are used to support the ex vivo expansion and activation of NK cells. These include soluble (often in cocktails), fusion, and membrane-bound versions. Soluble cytokine supplements generally contain IL-2, alone or in combination with other cytokines like IL-15 or IL-21; numerous examples are reported in Additional file 1: Table S1, Additional file 2: Table S2, Additional file 3: Table S3 and Additional file 4: Table S4. IL-2 is necessary for the expansion of NK-92-derived products [71]. Notably, the Dana Farber Institute has initiated two Phase I trials with PB-NK cells cultured in RPMI-1640 with 10% HS and pre-activated with a cytokine cocktail containing IL-12, IL-15, and IL-18 [24], defined cytokine-induced memory-like NK cells (CIML) for their responsiveness to cytokine restimulation (Additional file 1: Table S1, rows 9 and 17) [85]. More advanced solutions for cytokine administration are fusion proteins, such as the WU-PRIME GMP-grade heteromeric fusion protein complex developed by Wugen, combining IL-12, IL-15, and IL-18 receptor engagement [86] which have been shown to induce a 250-fold expansion of NK cells over 14 days in the expansion system WU-EXPAND, improved in vitro cytotoxicity and in vivo persistence compared to NK cells which had only been expanded [87]. WU-PRIME is used to pre-activate WU-NK-101 cells in RPMI-1640 medium for 12–16 h in the presence of 10% HS (Additional file 1: Table S1, row 2) [68]. Membrane-bound cytokines are often used with feeder cell systems, as summarized in the “Feeder cells” section.

Specific cytokine cocktails are needed for the differentiation of CD34^+^ cells into NK cells. In the three-step process developed by Glycostem Therapeutics, different cytokines are supplemented at each stage: stem cell factor (SCF), IL-7, FMS-like tyrosine kinase 3 ligand (Flt3L), and thrombopoietin (TPO) for the first stage, replacement of TPO with IL-15 for the second stage, then the addition of IL-2 for the third final stage. Low-dose cytokines (G-CSF, granulocyte–macrophage (GM)-CSF and IL-6) are supplemented throughout the entire process (Additional file 1: Table S1, row 27) [45]. The Radboud University uses a similar approach, but with the introduction of the ryl hydrocarbon receptor (AHR) inhibitor StemRegenin 1 (SR1) to promote maintenance and expansion of HSCs (Additional file 1: Table S1, rows 26 and 33) [38]. NK progenitor expansion is achieved by Deverra Therapeutics (Additional file 1: Table S1, row 25) using SFEM medium on vessels pre-coated with the Delta1ext-IgG ligand of the Notch receptor, and in presence of cytokines [84]. Strategies for further progenitor differentiation into mature DVX201 NK cells have not been disclosed.

iPSC-NK cells are also differentiated with specific cytokine protocols. With Fate Therapeutics’ technology, hematopoietic progenitor iCD34^+^ cells are first differentiated over 18–21 days, then enriched [88, 89] and differentiated into iNK cells by culturing them on EL08-1D2 murine stroma cells in B0 medium supplemented with 20% HS and cytokines such as IL-3, IL-7, IL-15, SCF, and FLT3L, for 21 days. Next, the iNK cells are harvested and co-cultured with irradiated K562 expressing IL-21 and 4-1BB in B0 media for 2–3 weeks (Additional file 1: Table S1, row 36: Additional file 2: Table S2, rows 19–27; Additional file 3: Table S3, row 37; Additional file 4: Table S4, rows 1–9) [66, 88].

Besides cytokines, other supplements such as vitamins, fusion proteins, and small molecules can be used to expand and activate NK cells ex vivo; a few examples from different categories are discussed below. Gamida Cell uses nicotinamide (NAM), a form of vitamin B3, to expand PB-derived GDA-201 NK cells (Additional file 1: Table S1, row 12) [90]. Preclinical studies showed how NAM stimulation, in combination with IL-15, produced highly functional NK cells both in vitro and in vivo [91], and how the addition of NAM to a two-week feeder-free culture with IL-2 and IL-15 enhanced the expansion of NK cells by 60–80-fold [92]. FATE-NK100 cells from Fate Therapeutics (Additional file 1: Table S1, row 21–22), are grown in presence of the Glycogen synthase kinase 3β (GSK-3B) inhibitor CHIR-99021, hence generating a subset of CD57-expressing NK cells that exhibit increased cytotoxicity against a variety of solid tumors. Preclinical data from 2017 showed an average expansion of tenfold relative to day 0 [77]. The TKD peptide, a 14-amino acid sequence from the Heat shock protein 70 (HSP70), has been demonstrated to stimulate the cytolytic, proliferative and migratory ability of NK cells against target cells, expressing the membrane form of HSP70 on their surface, through the engagement of CD94 [93, 94]. TKD-expanded and activated NK cells by the Royan Institute and Tehran University of Medical Sciences are tested against patient-derived glioblastoma cells with membrane-bound HSP70 (Additional file 1: Table S1, row 16) [95]. Moreover, priming agents, such as the IL-15 superagonist ALT-803 and the IL-2 recombinant fusion protein ALT-801 from Altor Bioscience, have been tested in combination with NK cells in a few clinical studies (Additional file 3: Table S3, rows 3 [96] and 6 [97]). Further details on these compounds and their mechanism of action will be discussed in the “Combination NK Cell Therapies” section.

#### Feeder cells

Feeder cells are lethally irradiated cells that become unable to divide but provide cell-to-cell contact and extracellular secretions, such as growth factors, cytokines, and ligands, that support proliferation and stimulation of co-cultured NK cells [98]. Feeder cells used to facilitate NK cell expansion are mostly autologous peripheral blood mononuclear cells (PBMCs) or tumor cell lines. Several properties of PBMC feeder cells are important for NK proliferation. Direct contact with CD3^+^ T cells and cytokine secretion result in NK expansion [99]; stimulation with anti-CD3 and IL-2, or DNA damage by irradiation, can increase expression of NKG2D ligands on T cells and therefore induce NK cell activation and proliferation [100]. The MG4101 PB-NK product from GC Pharma (Additional file 1: Table S1, row 14) is expanded with a combination of irradiated autologous PBMC with the anti-CD3 monoclonal antibody (mAb) OKT3 in SCGM medium with 1% auto-plasma and IL-2 stimulation [69]. The antibody stimulation activates the T cells present in the feeder layer to secrete cytokines, which consequently support NK cell expansion [69]. The K562 myelogenous leukemia cell line, however, is the most widely used feeder system. Genetic modification of K562 cells to express specific cytokines and ligands can further enhance expansion and cytotoxicity of co-cultured NK cells. Dario Campana’s team pioneered the field in 2009, engineering K562 to express 4-1BB ligands, NK-stimulatory molecules, and a membrane-bound form of IL-15 (K562-mbIL15-41BBL) [101]. This system is adopted in several NK manufacturing processes (Additional file 2: Table S2, row 11; Additional file 3: Table S3, row 7, 11, 44), also in combination with other technologies, such as the aforementioned HANK kit (Additional file 1: Table S1 row 24; Additional file 3: Table S3 rows 31–35) [82]. Additional modifications have been introduced by the MD Anderson Cancer Center to overcome the limitations associated with low numbers of NK cells available from cord blood units (Additional file 1: Table S1, row 34). In their process, UCB-NKs are co-cultured with K562 artificial antigen-presenting cells (aAPCs) expressing membrane-bound IL-21 (mbIL21), together with 4-1BB ligand, CD64 and CD86 surface co-expression (102). This GMP-grade cell line has been previously shown to promote cord blood-derived NK cell expansion up to 2000-fold in two weeks. Despite some differences in NK cell receptor expression compared to standard IL-2 stimulation, the cells maintained functional cytotoxicity and showed a lower exhaustion phenotype [102]. Fate Therapeutics employs irradiated K562-mbIL21-41BBL feeder cells together with IL-2 for the expansion of iPSCs-derived NK cells (Additional file 1: Table S1, row 36; Additional file 2: Table S2 rows 19–27; Additional file 3: Table S3, row 37; Additional file 4: Table S4, rows 1–9) [103]. In a similar manner, CytoSen has developed a K562-based feeder layer expressing mbIL21 and 4-1BBL and other co-stimulatory tumor ligands, known as Kiadis FC21 (Feeder cell 21) after acquisition from Kiadis (now Sanofi) [66]. FC21 is used in a 13-day co-culture with K-NK003 cells (Additional file 1: Table S1, row 6) [28], and with PB-NK cells for combination therapy (Additional file 3: Table S3, row 14) [104]. Interestingly, this approach was further evolved by CytoSen/Kiadis as the Plasma membrane 21 (PM21) technology, a cell membrane-derived preparation containing membrane fractions of FC21, preserving the native stimulation mechanism by presenting mbIL21 to the NK cells [104] in a feeder-free system. Preclinical studies showed an average 825-fold expansion after 14 days of ex vivo culture with PM21 [105]. Kiadis is currently employing this platform for the manufacturing of their NK cell products K-NK002 (Additional file 1: Table S1, row 4) [106] and K-NK003 (Additional file 1: Table S1, row 6) [107]. The GMP-grade Epstein–Barr virus-transformed lymphoblastoid cell line (EBV-LCL), from Fred Hutchinson Cancer Research Center, has been shown to expand PB-NK cell ex vivo between 815 and 3267-fold [108]; this system was first applied to NK clinical manufacturing by Dr. Richard Childs at the National Institutes of Health (NIH) in a trial evaluating the anti-tumor activity of infused autologous NK cells in combination with the proteasome inhibitor Bortezomib (Phase I trial, NCT00720785, not included in Additional file 1: Table S1, Additional file 2: Table S2, Additional file 3: Table S3 and Additional file 4: Table S4) and is currently licensed by ONK Therapeutics [109].

Despite the widespread use, feeder cells are confined to the product development phase and to early clinical stages, and their use should be overcome in advanced manufacturing processes. Although growth-arrested by γ-irradiation, residual contamination of feeder-derived impurities could be present in the final product. As reviewed by Liu et al., specific release criteria for feeder-cultured products should be developed. Methods can include labeling of feeder cells with a fluorescent tag or expression of a suicide gene. For these reasons, a feeder-free approach is a more desirable method to preserve safety and to improve control and robustness of the manufacturing process [110]. Feeder-free manufacturing of NK cells has been adopted by several NK cell manufacturers. Such systems rely on optimal, product-specific, combinations of cell culture medium, serum, small molecules, and cytokines supporting the generation of large numbers of cells without the need for co-culture systems.

### NK expansion and differentiation systems

The choice of platform for NK cell production relies on the design of an appropriate process and on the use of adequate hardware to ensure large-scale production, GMP compliance, and cost-effectiveness. The solutions currently used by NK cell manufacturers are highly heterogeneous and range from open-handling cell culture flasks to closed-system bioreactors. Although perhaps sufficient to supply early clinical phases (mostly Phase I/II studies), open systems are not compatible with large cohorts of patients and scale-up GMP processes necessary to sustain advanced clinical development and marketization. The present-day expansion and differentiation systems are described in the next sections. NK cell expansion rates, obtained as a combination of the product-specific culture process and of the platform of choice, are reported below, when available.

#### Flasks

Cell culture flasks require relatively low numbers of cells to initiate culture and guarantee low investment in equipment and materials [111]. However, flask-based systems are mostly open systems and carry the inherent risk of exposure to contaminants even in a high-class GMP-cleanroom environment, are labor-intensive, and, although compatible with scaling-out, do not allow process scaling-up, thus limiting the final number of cells that can be produced. Nevertheless, their use is reported in a few clinical trials. PB-NKs from Yonsei University and SMT Bio are expanded for 21 days in T75 and T175 flasks (Additional file 1: Table S1, row 20) [112]. HANK cells (Additional file 1: Table S1, row 24; Additional file 3: Table S3, rows 31–35) are expanded in T175 flasks for the first 7 days, then transferred to 2 L-cell culture bags for an additional 4 days of expansion and differentiation [82].

#### Static culture bags

Cell culture bags offer the possibility of a closed system, decreasing the risk of contamination while maintaining a high permeability to oxygen and carbon dioxide for optimal gas exchange. With reduced handling and reasonable investment in new equipment required, bags have been shown to support good expansion of NK cells [111]. Several products are available for NK cell manufacturers, who make use of bags for part of or for the entire ex vivo expansion process. Examples are VueLife bags from Saint-Gobain (Additional file 1: Table S1, rows 26–27 and 33) [45], NIPRO’s cell culture bags (Additional file 1: Table S1, row 14) [69], Perma-Life bags from OriGen (Additional file 1: Table S1, row 15) [31], and LifeCell X-Fold culture bags (Additional file 1: Table S1, row 25) [84].

#### Static and dynamic bioreactors

Adoptive cell therapy-suitable bioreactors provide controlled culture conditions favoring continuous nutrient supply and waste product removal, as well as physiological cues to guide cell growth [113, 114]. Static bioreactors facilitate three-dimensional cell culture, although medium renewal cycles cause frequent nutrient and metabolite fluctuations that may trigger high phenotypical variability [114] and limit cell growth. The most used static platform is the Wilson Wolf Gas Permeable Rapid Expansion Platform, known as G-Rex®, consisting of a gas permeable membrane surface of different sizes which allows optimal gas exchange. GDA-201 cells from Gamida Cell (Additional file 1: Table S1, row 12) are produced in a G-Rex® 100 M-CS device, with a GMP-compliant manufacturing process allowing to perform only one feeding in the 14–16 days of culture of CD3-depleted PB-NK cells without impacting the cell expansion, which reaches 50-fold [30]. Similarly, UCB-NK cells generated from 20 million cord blood-derived mononuclear cells (CB-MNC) are cultivated in 400 mL inside a GP500 gas permeable reactor (the former market name for the G-Rex®100 M platform) by the MD Anderson Cancer Center (Additional file 1: Table S1, row 34). Previous results from the group showed around 2000-fold expansion for both frozen and fresh CB-MNC cells with this manufacturing system [103].

Dynamic culture systems such as rocking and spinning bioreactors [115] are scale-up-compatible platforms that offer the ability to precisely control process parameters, thus allowing mimicking of in vivo conditions better than in static cultures, which could be beneficial for product quality [114]. The use of dynamic systems allows for continuous mixing, hence generating homogeneous culture conditions and ensuring optimal gas transfer, while requiring minimum hands-on time. Automatic culture and sampling are also possible. Despite the many advantages, the initial investment can be significantly higher than with other platforms and the amount of cells required to initiate the culture could be limiting for certain NK cell sources. Nevertheless, multiple studies have shown that immune cells, such as NK cells and T cells, seem to achieve a higher proliferation rate in bioreactors compared to static bag cultures [115]. Glycostem Therapeutics has developed an innovative platform to expand and differentiate clinical-grade NK cells from progenitor cells using the Xuri rocking bioreactor system after a progenitor expansion phase in static cultures, resulting in a 3–4-log expansion from a single cord blood unit (Additional file 1: Table S1, row 27) [39]. The Xuri system has also been implemented in the expansion process of the K-NK cell products from Kiadis Pharma (Additional file 1: Table S1, rows 4, 5): after automated CD3^+^ cells depletion in the CliniMACS Prodigy®, cells are first expanded in the CentriCult chamber of the Prodigy [116] for 7 days, then in 10 L Xuri Cell Expansion System W25 for another week at which point PB-NK cells reach 1000-fold expansion [28]. The AlloNK™ platform from Artiva Biotherapeutics (Additional file 3: Table S3, row 43) makes use of 50 L bioreactors to expand the master cell bank originating from static cultures of UCB-NK cells [117].

### Final formulation and preservation

After expansion and maturation, therapeutic NK cells are harvested, concentrated, and formulated in the final product which is usually filled in bags at the defined therapeutic dose. Formulation and preservation strategies for cell immunotherapies were reviewed by Li and colleagues [118]; approaches used for fresh and cryopreserved NK products currently in the clinic are presented below.

Product concentration after harvest increases the number of cells per volume in the final formulation. This allows the infusion of smaller volumes into patients, which is particularly beneficial for the local treatment of solid tumors where the cavity space is limited. Additionally, concentration introduces one or more washing rounds, contributing to the removal of cell culture contaminants, serum, and cytokines. Several cell washing and concentration platforms are available, as reviewed by Li et al. [119], including the automated cell processing system Lovo from Fresenius Kabi, used for the formulation of the K-NK002 and K-NK003 products from Kiadis Pharma (Additional file 1: Table S1, rows 4, 5) [28, 39].

The final product formulation often contains excipients to preserve the cell metabolism and to stabilize them through handling and change of environmental conditions, especially for cryopreserved products. Human serum albumin (HSA) is commonly used in the formulation of clinical-grade NK cells, normally in combination with a saline solution. Cord blood-derived UCB-NK from MD Anderson (Additional file 1: Table S1, row 34) are prepared with lower HSA (0.5%) in a commercial isotonic solution containing electrolytes called Plasma-Lyte [102]. As part of their HANK kit, HANK Bioengineering has developed a solution that can be added to the final product to support the NK cells. HANK cells are formulated in saline solution with 1% HSA and 6 mL of HK-003, in a total volume of 400 mL, before infusion into patients (Additional file 1: Table S1, row 24) [82]. The Asan Medical Center (Additional file 1: Table S1, row 23 and Additional file 3: Table S3 rows 16, 17, 21) uses a preparation with 5% HSA in 100 mL of undisclosed dilutant for their PB-NK derived cellular product [25].

#### Cryopreservation

Cryopreservation allows true off-the-shelf product delivery, providing cost and time efficiency for patients and for cell manufacturers and allowing to treat patients with multiple doses of the same batch. Despite the advantages, there are technical limitations to its successful application. Cells can be damaged during the freezing and thawing processes, mainly because of cell dehydration at a slow rate of freezing and/or due to the formation of intracellular ice crystals when freezing is too rapid. Controlled rate freezing using commercial devices provides a solution by maintaining a stable cooling rate (generally – 1 °C/min) to minimize both effects [120]. Different studies have proven the influence of the cooling rate on the recovery of a variety of cell types after thawing, as reviewed by Li and colleagues [118]; with this in mind, the freezing/thawing process must be optimized for each NK cell product. Additionally, there are concerns regarding the effect of cryopreservation on the phenotype and activity of NK cells after thawing. The addition of cryoprotectant agents (CPAs) to the product formulation is required to guarantee cell survival during freeze–thaw processes [121]. The most used CPA is dimethyl sulfoxide (DMSO). DMSO is toxic to cells; therefore, the pre-freeze and post-thaw time window should be optimized to minimize biochemical toxicity to NK cells [118]. Furthermore, its use has been associated with toxicity in patients [122, 123]. Post-thaw washing and CPA removal are complex, although possible [122]. DMSO-free cryopreservation of T and NK cells is still challenging, as reviewed by Yao and Matosevic [124]. The University Hospital of Basel infuses cryopreserved PB-NK cells (Additional file 1: Table S1, row 15) in a formulation containing 2% HSA and 7.5% DMSO in phosphate saline buffer (PBS) [31]. Similarly, GC Pharma’s MG4101 (Additional file 1: Table S1, row 14; Additional file 3: Table S3, row 29) is cryopreserved in RPMI-1640 freezing medium with 20% HSA, 25% dextran-40, and 5% DMSO [125]. Celularity has patented different formulations for freezing the UCB-CD34^+^-derived product CYNK-001 (Additional file 1: Table S1, rows 28, 29, 32) including dextran-40, HSA, and DMSO [126]. It is not disclosed if these products are directly infused after thawing or washed to remove DMSO.

## Non-engineered NK cell therapies

### NK cell sources and clinical applications

Thanks to their potential to naturally kill cancer cells, NK cells can be directly infused into patients without the need for genetic modification, alone or in combination with agents that can enhance their cytotoxic capacity. The 36 clinical trials employing non-engineered NK cell therapies as monotherapy that were registered on ClinicalTrials.gov after March 2017 are grouped per NK cell source and described in Additional file 1: Table S1. Additionally, the statistics of non-engineered NK cell clinical development are reported in Fig. 2. As shown in Fig. 2A, the most frequently used NK cell source is PB-NKs (67% of the trials), followed by hematopoietic stem cells (HSCs) (25%); UCB-NK and iPSCs only represent a small fraction (5% and 3%, respectively). No trials with non-engineered NK-92 as monotherapy have been registered on ClinicalTrials.gov after March 2017. The majority of the 36 trials, 65%, are investigating the therapeutical potential of NK cells against hematological malignancies, and only 35% aim to target solid tumors (Fig. 2B). Among hematological malignancies (Fig. 2C), AML is reported as the main indication for almost half of the trials (48%), followed by myelodysplastic syndromes (MDS) (20%) and multiple myeloma (MM) (10%). Other types are poorly represented (up to 3%). From the overview of the solid tumors (Fig. 2D), non-small-cell lung cancer (NSCLC) and liver cancer are the most targeted (16% each), followed by ovarian cancer, glioblastoma, and head and neck cancer (HNC) (11%). Other solid malignancies, namely melanoma, renal cell, colorectal, breast, biliary tract (BTC) and pancreatic cancers, and Merkel cell carcinoma (MCC), are less frequent (5%).

## Engineered NK cell therapies

### CAR-T versus CAR-NK cell therapies

A chimeric antigen receptor (CAR) is a fusion protein engineered to be expressed on the surface of immune cells. A CAR contains an extracellular domain capable of targeting a specific antigen using the variable region of a monoclonal antibody, a transmembrane domain, and one or more intracellular signaling domain(s) that are activated upon antigen binding [127]. Depending on the number of intracellular co-stimulatory and signaling domains, different generations of CARs have been developed. The core component of most CARs is the intracellular domain of the T cell co-receptor CD3ζ, which contains three immunoreceptor tyrosine-based activation motifs (ITAMs) that are important for signal transduction. Second- and third-generation CARs have one or two co-stimulatory domains in addition to CD3ζ. Fourth- and fifth-generation CARs are additionally paired with chemokines or cytokine receptors [128]. Triggering of the signaling domain(s) initiates effector functions such as activation, cytokine release, survival and proliferation of the CAR-expressing cell, and most importantly antigen-specific cytolysis of tumor cells [127]. Historically, the introduction of CARs in autologous T cells (CAR-T cells) pioneered the CAR-based cellular immunotherapy field [129, 130] and as of August 2022, 5 CAR-T products have been approved by the US Food and Drug Administration (FDA): ABECMA® [131] to treat adult patients with MM, BREYANZI® [132] for adult relapsed or refractory large B cell lymphoma, KYMRIAH™ [133, 134] for adult refractory diffuse large B cell Lymphoma (DLBCL) and young adult acute lymphoblastic leukemia (ALL), TECARTUS™ [135] for adult relapse/refractory mantle cell lymphoma and YESCARTA™ [136, 137] for B cell lymphoma. All these CAR-T cell therapies are genetically modified autologous T cells with 2^nd^-generation antigen-specific synthetic CAR receptors targeting CD19. For example, KYMRIAH™ contains the CD3ζ domain to initiate T cell activation and the 4-1BB co-stimulatory domain to enhance expansion and persistency [133], whereas YESCARTA™ carries the CD28 co-stimulatory signal instead of 4-1BB [137]. Long-term safety data for all FDA-approved CAR-T cell products have shown durable and high response rates. 5-year safety data of children and young adults who would have had limited treatment options, showed an overall survival rate of 55% after treatment with KYMRIAH™, demonstrating the promising curative potential of CAR-T cells [138].

Despite the clinical benefit, CAR-T cell therapies have shown major limitations that were not fully anticipated by preclinical studies. Autologous T cell sourcing is associated with high production costs, long manufacturing time, and reduced fitness of the cells due to heavy pretreatment of patients before attempting adoptive cell transfer [139]. Safety risks are associated with the development of cytokine release syndrome (CRS) and neurologic toxicity [139, 140]. Moreover, allogeneic CAR-T cells could potentially cause GvHD in the post-hematopoietic cell transplant (HCT) setting using recipient or donor-derived CAR-T cells, as well as in non-HCT setting using allogeneic T cell therapies [141]. NK cell-based CAR therapies represent a promising alternative to the limitations of CAR-T, since clinical-grade off-the-shelf products can be generated from multiple allogeneic sources with a favorable safety profile and low risk for GvHD, neurotoxicity, and CRS. NK cells produce a different set of cytokines not related to CRS [142] and can combine the specificity of tumor targeting with inherent innate anti-tumor effector potential [143–145]. However, efficient treatment of solid tumors remains a challenge. Tumors can limit lymphocyte effector functions by generating immunosuppressive cytokines impairing T and NK cell migration and by altering the expression of tumor-associated antigens (TAAs) or MHC class I molecules [146]. Nevertheless, advancements in CAR design have shown improved anti-tumor activity in preclinical studies for various indications including neuroblastoma [147], glioblastoma [148–150], breast [151], ovarian [152], CRC [153], lung [154] and liver cancer [155], and MM [155]. Several strategies to overcome the immunosuppressive tumor microenvironment (TME) have been developed for engineered products. Chimeric NKG2D receptor-expressing NK cells target and eliminate myeloid-derived suppressor cells (MDSCs), which overexpress NKG2D ligands within the TME, improving the anti-tumor function of CAR-T cells [147]. Co-expression of CAR with chemokine receptors [149], or with cytokines and chemokines (IL-7 and CCL19) [156] aims to improve survival and migration to the TME. Dual CARs/scFv’s recognize multiple antigens to avoid tumor escape [148, 150], while chimeric co-stimulatory converting receptors (CCCRs) aim to convert the cancer immune escape by PD-1/PD-L1 binding into an activating stimulatory signal [153]. Additionally, armored CAR-T cells secrete an immune checkpoint (PD-1) blockade scFv to overcome the cancer immune escape [157]. Such strategies offer promising solutions to advance future solid tumor treatment with CAR-NK therapies.

**Fig. 4:**
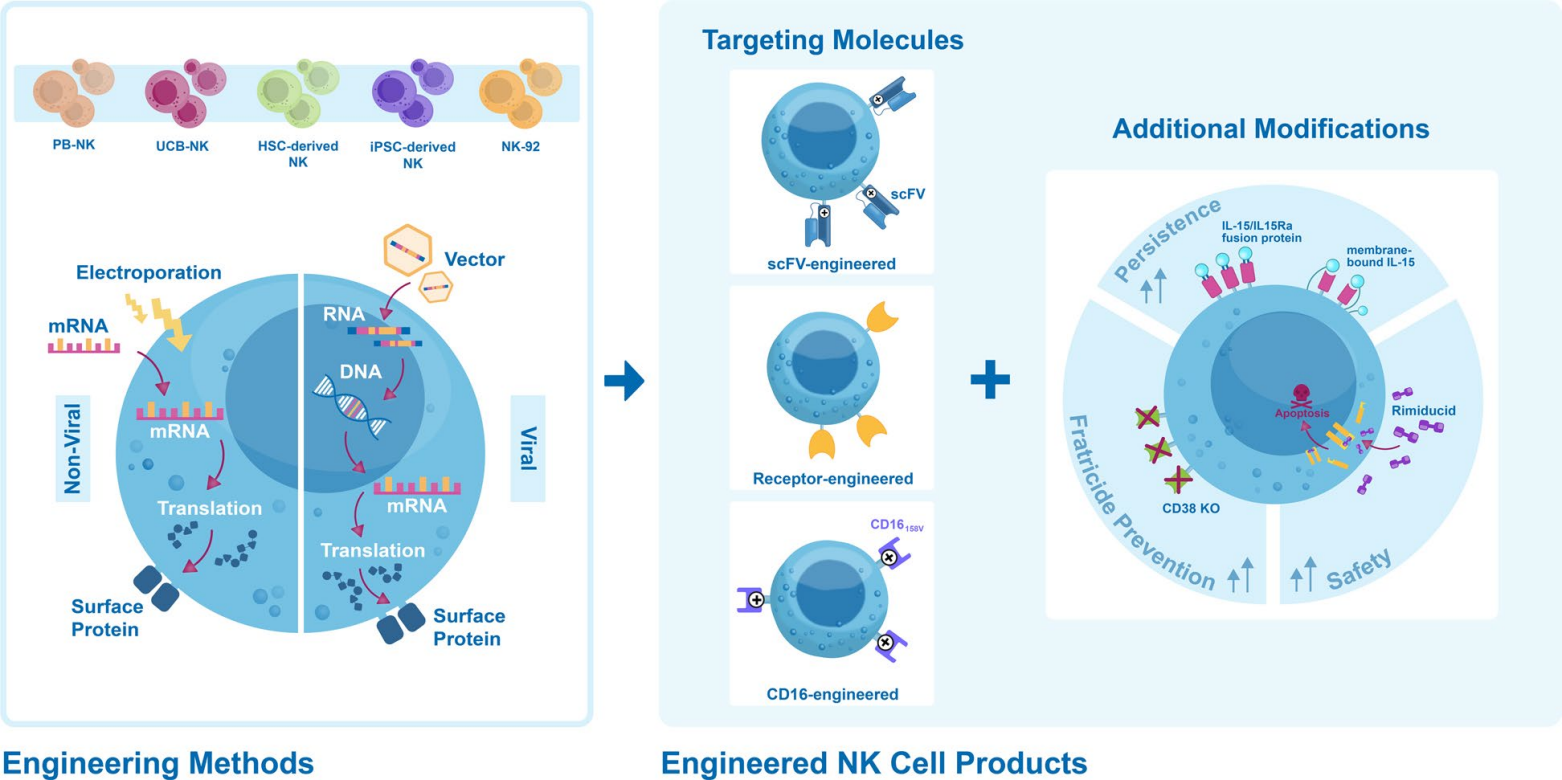
Overview of engineered NK cell therapies. From left to right: different NK cell sources (PB-NK, UCB-NK, HSC-derived NK, iPSC-derived NK, NK-92) are engineered to express the desired surface protein(s) by means of non-viral electroporation with mRNA (left) or of transduction with γ-retro or lentiviral vectors and stable integration in the host genome (right). Engineered NK cell therapies can be divided in categories, according to the targeting molecule: (1) antibody-derived single-chain variable fragment (scFv-engineered) for tumor antigen binding, (2) NK cell receptor (receptor-engineered) for ligand binding, and (3) Fc receptor CD16 (158 V) high-affinity non-cleavable hnCD16 variant (CD16-engineered) to enhance antibody-dependent cell cytotoxicity (ADCC). Co-expression of scFv and hnCD16 is also reported. Additional modifications of engineered NK cell products under clinical evaluation enhance NK cell persistence in vivo by expression of membrane-bound IL-15 or of IL-15/IL-15 receptor fusion protein IL-15/IL-15Ra, prevent fratricide by CD38 KO, or increase safety through induction of apoptosis by administration of Rimiducid

The technological and biological characteristics of engineered NK therapies that have reached the clinical stage, including the cell sources, the engineering methods, the types of engineered NK cells and their targets as well as additional modifications, have been gathered from clinical trial reports, publications, or press releases, collected in Additional file 2: Table S2 and summarized in Fig. 4, and described in the next sections.

### Engineered NK cell sources and clinical applications

**Fig. 5:**
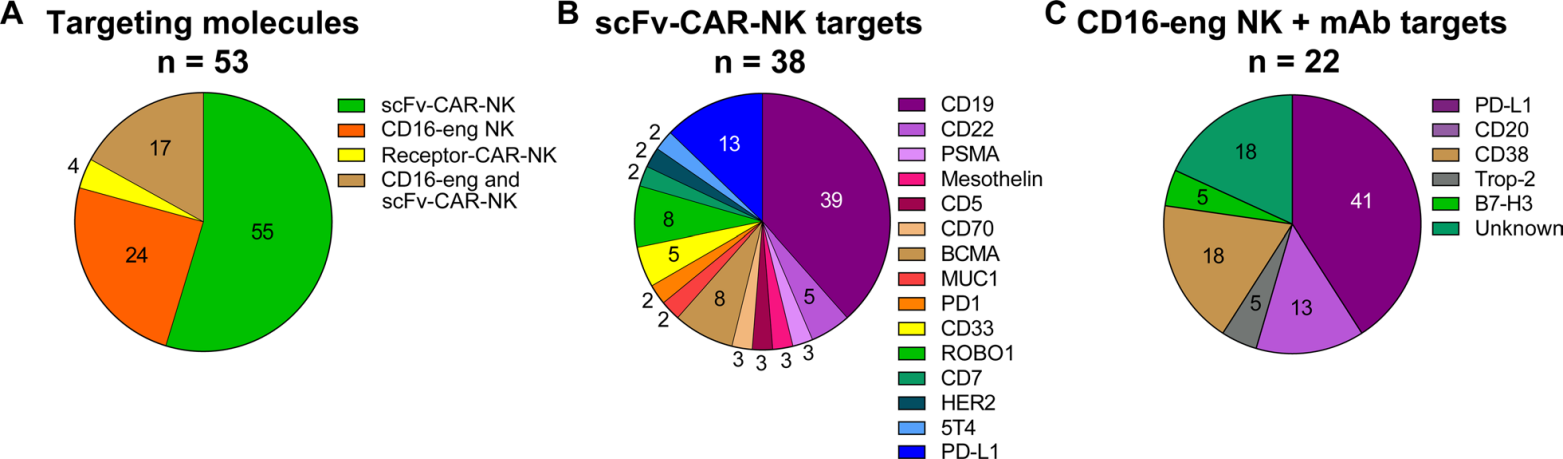
Statistics of engineered NK cell therapies. The pie charts present the types of engineered products under clinical evaluation, as reported on ClinicalTrials.gov until December 2021 (*N* = 53 trials). For every pie, numbers indicate the percentage of the whole taken by each slice. **A** Targeting molecules expressed on the surface of engineered NK cells: scFv-engineered chimeric antigen receptor (CAR)-NK, receptor-engineered CAR-NK, CD16-engineered NK and CD16-engineered and scFv-CAR-NK. **B** Breakdown of the surface tumor antigens targeted by scFv-engineered CAR-NK therapies. **C** Breakdown of the surface tumor antigens targeted by monoclonal antibodies used in combination with CD16-engineered NK cells. Undisclosed targets are labeled as “Unknown.” In B and C, one trial can have more than one target, belonging to more than one category

As of December 2021, 53 clinical studies with engineered NK cells used as monotherapy have been registered on ClinicalTrials.gov; they are summarized in Additional file 2: Table S2, where the available information about the products and the trial status is collected. Field statistics are presented in Figs. 2 and 5. NK-92 cells are the most frequently used source of engineered NK therapies in clinical trials (43% of trials), followed by PB-NKs (21%), iPSCs (17%), and UCB-NKs (13%), whereas engineered HSC-derived NK cell products are still mainly at the preclinical stage (Fig. 2A). Engineered NK cell products are almost equally represented (55% vs. 45%, respectively) as a treatment for hematological malignancies and solid tumors (Fig. 2B). Engineered NK cells are mainly used in lymphomas such as B cell lymphoma (23% of total), other lymphomas (13%), NHL (13%) and in various leukemias, such as AML (16%) (Fig. 2C). Regarding solid tumors (Fig. 2D), immunotherapy-resistant, difficult to treat pancreatic cancer, is the most frequently targeted indication (26%), followed by NSCLC and liver cancer (11%), MCC (8%), colorectal, prostate, breast and head and neck cancers (7% each).

### NK cell engineering methods

Currently, two main gene delivery methods are used for clinical-grade engineering of NK cells to express CAR molecules, namely non-viral electroporation and viral vectors. Electroporation can be used to deliver different cargos, namely transposon/transposase systems as Sleeping Beauty and piggyBac, or messenger RNA [158]. Transposons have been used in CAR-T setting [159, 160]; however, CAR-T cell lymphoma was developed by patients treated with piggyBac-modified cells [160], possibly related to insertional oncogenesis, where a high vector copy number was found in one of such patients [161]. Although transposons have not yet reached clinical development with NK cells, preclinical data have shown CAR-NK functionality and feasibility despite some challenges to overcome in terms of engineering efficiency and stable transgene expression (162, 163). mRNA delivery via electroporation is a simple and cost-effective clinic-compliant technology [164], although its non-integrative and non-replicative nature results only in a transient transgene expression on the cell surface [165]. In addition, electroporation can have direct effects on membrane permeabilization and cell viability. In contrast, viral delivery methods allow long-lasting stable transgene expression by introducing genetic material with γ-retroviral or lentiviral vectors. The use of γ-retroviral vectors is limited by the dependency on cell proliferation, as they can only access the cell’s genome while the nuclear membrane is dissolved [165]. Lentiviral vectors are more versatile, as they can integrate in both dividing and non-dividing cells. For clinical purposes, the development of third-generation lentiviral vectors significantly reduced the risk of generation of replication-competent virus. In addition, 3’ long terminal repeats (LTR) deletions of self-inactivating (SIN) lentiviral vectors further improve safety [166]. The different engineering methods and their preclinical and clinical application in NK cell therapies have been recently reviewed by Rezvani et al. [167] and Mantesso et al. [168]. Details of the cell engineering methods are often not disclosed, but the available information is presented below.

Only two trials have reported the use of mRNA electroporation to engineer NK cells, both with PB-NKs transfected with a transgene mRNA. In the study by The Third Affiliated Hospital of Guangzhou Medical University, the mRNA was coding a CAR based on the NKG2D C-type lectin-like receptor (Additional file 2: Table S2, row 1). Increased NKG2D mean fluorescence intensity (MFI, > 1.2-fold higher than control) and > 95% delivery efficiency was obtained after 1 day, and cell viability was > 90% prior to infusion [169]. In the other trial by St. Jude Children's Research Hospital, cells were electroporated with a CAR against CD19 (Additional file 2: Table S2, row 11), but no information regarding the efficiency of this engineering method has been disclosed.

Retroviral vectors have been chosen for the engineering of UCB-NK cells by Nkarta Therapeutics for products NKX101-101 and NKX019 (Additional file 2: Table S2, rows 8 and 9), by the MD Anderson Cancer Center and Takeda (Additional file 2: Table S2, rows 12–16), and by Wuhan Union Hospital (Additional file 2: Table S2, row 18). Cell transduction efficiency and vector copy number (VCN) information is unfortunately not disclosed; only the MD Anderson Cancer Center reported transduction efficiencies between 22.7 and 66.5% for study NCT03056339 (Additional file 2: Table S2, row 12) [170]. The use of lentiviral vectors is reported by several manufacturers. Fate Therapeutics has engineered the iPSC-derived NK cell product FT516 (Additional file 2: Table S2, rows 21–23) with high-affinity, non-cleavable CD16 (hnCD16). Preclinical data report > 99% CD16^+^ clonal iPSCs upon lentiviral transduction, with supporting integration site analysis that showed 3 copies of hnCD16 per genome [171]. The Xinxiang Medical University has engineered NK-92 cells with a CAR directed against PD-1 (Additional file 2: Table S2, row 29). Preclinical data for the anti-PD-1 CAR-NK-92 cells reveal roughly 80% PD-1 expression, a similar phenotype compared to untransduced NK-92 cells (CD56^+^/CD16^−^) and no significant difference in proliferation rate in the first 72 h [153]. Anti-CD33 CAR-engineered NK-92 cells from PersonGen BioTherapeutics (Additional file 2: Table S2, row 30) clinically showed over 90% CD33 expression; however, no information on VCN or viability was shared [172]. The Johann Wolfgang Goethe University Hospital (Additional file 2: Table S2, row 35) has transduced NK-92 cells with an anti-HER2 CAR. Although the use of lentiviruses was disclosed, no other information on transduction efficiency, VCN, or viability is available.

### Engineered NK cell types

As CARs were initially developed for T cell applications, their design is generally still optimized for expression in T cells, although NK cell-specific constructs are emerging. Chimeric CAR molecules are designed to carry a part of an antibody in the form of a single-chain variable fragment (scFv) in the extracellular domain for antigen recognition, and a part of the T cell receptor (TCR) complex in the intracellular signaling domain to transduce activation signals to the CAR-expressing cell [173]. The extracellular domain is connected to the hinge to provide structural conformation, while the transmembrane region provides anchorage in the plasma membrane, bridging the extra- and intracellular signaling domains [173]. The intracellular domain translates the activation signals inside the cell and is generally derived from CD3ζ ITAM domains, possibly combined with one or more co-stimulatory domains [173]. Fujiwara et al. highlighted the importance of CAR construct design, specifically the role of hinge and transmembrane domains on CAR expression and stability as well as functionality [174]. The engineered NK products that have currently reached the clinical stage can be divided into three categories, based on the nature of the targeting molecule(s) expressed on their surface: two types of CAR-NK cells, namely scFv-engineered and receptor-engineered, and CD16-engineered NK cells.

#### scFv-engineered CAR-NK cells

scFv-CARs are used in most of the engineered NK products in clinical trials, as they are evaluated in 55% of the studies as single surface targeting molecules, and in additional 17% of cases in co-expression with CD16 (Fig. 5A). The scFv is constructed from the variable heavy (V_H_) and light (V_L_) chains of a conventional mAb. As scFvs may behave differently than mAbs, a successful and validated mAb is no guarantee for a functional scFv. Selection of the right affinity for the antigen epitope is necessary to achieve optimal binding to the target TAA and to avoid on-target off-tumor toxicities for antigens expressed on both tumor and healthy cells [175]. It has been reported that the variable domains from the scFv can interact with each other through a phenomenon known as domain swapping, which can lead to CAR oligomerization [176] and might translate into inferior clinical efficacy [177]. Additionally, hydrophobic interactions with receptors cause antigen-independent activation, resulting in the so-called tonic signaling [175, 178], and can be a cause of poor anti-tumor efficacy, impaired survival, and reduced persistence in vivo [179]. The use of murine scFvs may cause immunogenicity; lymphodepletion regimens administered before infusion of NK cells to eliminate immunosuppressive cells can reduce its occurrence [178]. To overcome such human anti-mouse antibody (HAMA) responses, humanized scFvs have been developed, containing the complementarity determining regions (CDRs) from mouse to retain the antigen-binding affinity but combined with a human framework region [180].

Clinical-stage scFv-engineered CAR-NK products have been designed to target a variety of antigens on the surface of target cancer cells, for both hematological and solid tumors (Fig. 5B). The most frequently chosen target is CD19 (39%) targeting B cell lymphoma, followed by PD-L1 (13%) and ROBO1 (8%) targeting pancreatic cancer and other advanced solid tumors and by BCMA targeting MM (8%). At least 11 more antigens are explored as CAR-NK targets, including CD33 and CD22 (with a frequency of 5% each).

#### Receptor-engineered CAR-NK cells

NK cells are naturally equipped with surface receptors binding specific ligands on tumor cells, based on pattern recognition. However, engineering NK cells with a CAR carrying the ligand-binding domain from an activating NK cell receptor linked to co-stimulatory and activating intracellular domain(s) originated from other receptors, can increase targeting and signal transduction efficiency of anti-tumor responses. Unlike murine-derived scFvs, using NK cells’ natural receptors could reduce the risk of developing an immune response against the genetically modified cells, leading to reduced effectiveness [181]. In addition, these CARs have shown to be structurally stable and can recognize stress-induced or overexpressed ligands on multiple types of tumors, hereby targeting multiple indications [182]. One limitation of receptor-engineered CARs comes from their ability to also target ligands on healthy cells, leading to on-target off-tumor toxicities [181]. Until today, two CAR-NK products carrying the NKG2D receptor antigen-binding moiety on their extracellular domain have entered the clinical phase (4% of total, Fig. 5A). Both are PB-NK-derived NKG2D-CAR-NK, from The Third Affiliated Hospital of Guangzhou Medical University for the treatment of metastatic solid tumors (Additional file 2: Table S2, row 1) and Nkarta Therapeutics’ NKX101-101 for AML and MDS (Additional file 2: Table S2, row 8).

#### CD16-engineered NK cells

CD16a is an endogenous surface receptor expressed by cytotoxic NK cells which recognizes the constant fragment crystallizable (Fc) region of immunoglobulin (Ig)G antibodies bound to TAAs on target cancer cells, thereby initiating a potent cascade of signals resulting in anti-tumor responses mediated through cytokine secretion and recruitment of adaptive immune cells, known as antibody-dependent cellular cytotoxicity (ADCC) [183]. The excessive response is limited by a disintegrin and metalloprotease-17 (ADAM17)-mediated cleavage upon activation, resulting in CD16 surface expression downregulation [184]. The great anti-tumor potential of enhancing ADCC responses in vivo makes CD16 a main clinical focus for the development of NK cell therapies. CD16 is expressed at low levels on CD56^bright^ NK cells but is highly expressed on the CD56^dim^ NK cell subpopulation. To support the need for sustained surface CD16 expression, and to overcome susceptibility to ADAM17 cleavage, investigators have developed genetically engineered NK cells expressing a high-affinity, cleavage-resistant CD16 variant, hnCD16, carrying a point mutation in the extracellular domain (158 V). Of note, shedding of CD16 can potentially facilitate serial engagement of NK cells to tumor cells via dissembling of the NK cell immune synapse, thus hnCD16-expressing NK cell products might have an impaired serial killing functionality which might hinder their cytolytic efficacy [185]. Nevertheless, there are multiple ongoing clinical trials testing the safety and the efficacy of CD16-engineered NK cells, administered in combination with mAbs against the targeted tumor antigen (see “Combination NK cell therapies” section). As shown in Fig. 5A, CD16-engineered NK cells are developed either carrying CD16 as the single surface targeting molecule (24% of total), or carrying two surface targeting molecules, both hnCD16 and a scFv-CAR (17%). Overall, most clinical trials with CD16-engineered NK cells are combined with mAbs to be directed against PD-L1 (41%), followed by CD38 (18%), CD20 (13%), and Trop-2 and B7-H3 (5% each) (Fig. 5C). Notably, Fate Therapeutics’ FT516 and derivative products FT596, FT538 and FT576 are all iPSC-NK cells engineered to express hnCD16. FT516 has entered clinical trials against B cell lymphoma and AML in combination with antibodies (Additional file 2: Table S2, row 21; Additional file 4: Table S4, row 1), and in solid tumors, with antibodies targeting PD-L1 and B7-H3 (Additional file 2: Table S2, rows 22–23; Additional file 4: Table S4, rows 2–3). FT596 and FT576 are engineered to carry two targeting molecules on their surface, both hnCD16 and a scFv-CAR, anti-CD19 (Additional file 2: Table S2, row 19–20; Additional file 4: Table S4, rows 4–5) and anti-BCMA (Additional file 2: Table S2, row 27; Additional file 4: Table S4, row 9) respectively, for simultaneous antigen targeting via mAb and ADCC and of CD19 or BCMA TAAs via scFv. FT538, targeting CD38 via ADCC in AML and MM and in solid tumors (Additional file 2: Table S2, rows 24–26 and Additional file 4: Table S4, rows 6–8), and FT576, are engineered with CD38 knock-out to avoid fratricide (see “Additional modifications”). NK-92 cells do not express CD16 on their surface and therefore cannot mediate ADCC [51]. Similarly to Fate, ImmunityBio has developed a solution to express CD16 on the surface of NK-92-derived products by engineering high-affinity NK cells (haNK) and target high-affinity NK cells (t-haNK) with hnCD16 [71]. haNK single targeting molecule cells have been administered with monoclonal antibodies against solid tumors in multiple clinical trials (Additional file 2: Table S2, rows 44–50; Additional file 4: Table S4, rows 12–14, 18–19, 27, 29). Upon further co-engineering with CD19 or PD-L1 scFv-CARs, t-haNK cells carry two targeting molecules, combining ADCC with targeted antigen killing; t-haNK cells have so far been tested against multiple solid and hematological malignancies (Additional file 2: Table S2, rows 38–43; Additional file 4: Table S4, rows 32–34) [50, 186, 187].

#### CAR intracellular domain

Downstream of the antigen targeting moiety, CAR transmembrane and intracellular domains can vary between NK cell products. Unfortunately, the full CAR design and structure is not disclosed for all products. From the available information collected in Additional file 2: Table S2, most of the CAR-NK constructs are derived from T cell applications and contain the CD3ζ intracellular domain, which can also facilitate other NK cell receptor-mediated effector functions via CD16 and natural cytotoxicity receptors (NCRs) as NKp30 and NKp46 [188]. CD3ζ is combined with one or two co-stimulatory domains (CD28, 4-1BB, OX40, or CD27) in the second-generation designs; only one product is known to carry a third-generation CAR containing both CD28 and 4-1BB co-stimulatory domains coupled to the CD3ζ signaling domain (CD33-scFv CAR from PersonGen BioTherapeutics, Additional file 2: Table S2, row 30). NK biology-pertinent signaling domains are also used, such as the DAP12 signaling domain (Additional file 2: Table S2, row 1) and an NKG2D-derived transmembrane domain coupled with a 2B4 co-stimulatory domain before CD3ζ (Additional file 2: Table S2, row 19). Notably, in vitro data from Xu et al. and Huang et al. reported enhanced cytotoxic activity of NK-92 cells containing the 2B4 co-stimulatory domain compared to the CD28 or 4-1BB domains [189, 190]. Additionally, PB-NK cells expressing a CAR containing the DAP12 signaling domain demonstrated enhanced in vitro cytotoxic activity compared to CD3ζ signaling domain [169].

### Additional modifications

#### Persistence

Mature NK cells have a limited lifespan and low persistence in vivo [44, 191]; although this feature can be beneficial for patient safety, investigators have pursued methods to increase NK longevity, sustained clinical responses, and anti-tumor efficacy. In order to achieve this, the administration of cytokines (generally IL-2) to patients after NK cell infusion is frequently added as part of clinical protocols. High doses of recombinant IL-2 are associated with considerable toxicities, mostly in the heart, lungs, kidneys, and central nervous system [192–194], while ultra-low doses resulted in increased expansion and mobilization of inhibitory Tregs, affecting NK cell functionality in healthy individuals [195]. IL-15 has emerged as an interesting alternative to IL-2, since it does not induce suppressive Tregs [196] and has been shown to improve the cytolytic performances of NK cells [197]. NK cell engineering, however, offers the opportunity to sustain NK cells with cytokines without the need for intravenous administration. Preclinical data for co-expression of CAR with IL-15 in NK cells showed long-term persistence and improved anti-tumor activity compared to exogenous IL-15 administration in a CAR-only in vivo model [145]. Today, there are several NK products engineered with cytokines to improve persistence. CAR-NK products from Nkarta Therapeutics (Additional file 2: Table S2, rows 8 and 9), the MD Anderson Cancer Center and Takeda (Additional file 2: Table S2, rows 12–16; Additional file 4: Table S4, row 11) and Wuhan Union Hospital (Additional file 2: Table S2, row 18) contain a cassette for the co-expression of membrane-bound IL-15 (mbIL-15) together with the scFv- or receptor-CAR. Fate Therapeutics’ FT596, FT538, and FT576 are engineered to express a membrane-bound IL-15 receptor fusion (IL-15RF) together with hnCD16 and CD38 KO (FT538, Additional file 2: Table S2, rows 24–26; Additional file 4: Table S4, rows 6–8) or with hnCD16 and anti-CD19 CAR (FT596, Additional file 2: Table S2, rows 19–20; Additional file 4: Table S4, rows 4–5) or with hnCD16 and anti-BCMA CAR and CD38 KO (FT576, Additional file 2: Table S2, row 27; Additional file 4: Table S4, row 9). The IL-15RF was constructed by combining IL-15 and IL-15 receptor α (IL-15RA) [198], which together are shown to further improve anti-tumor potency, survival and proliferation in transduced CD8^+^ T cells compared to IL-15 or IL-15RA alone [199]. Since NK-92 cells require IL-2 for growth and cytotoxicity responses [200], stemming from a pivotal study by Konstantinidis et al. [201], ImmunityBio’s haNK and t-haNK platform is engineered to express an endoplasmic reticulum (ER)-retained IL-2 (ERIL-2) in addition to hnCD16 (haNK cells, Additional file 2: Table S2, rows 44–50; Additional file 4: Table S4, rows 12–31) or hnCD16 158 V and scFv-CAR (t-haNK cells, Additional file 2: Table S2, rows 38–43; Additional file 4: Table S4, rows 10, 32–34), in presence or absence of additional modifications.

#### Safety

Despite the limited toxicity of NK cells, the introduction of the CAR can cause potentially unwanted effects, mainly due to on-target off-tumor effects. As a safety measure, CAR-NK cells can be rapidly suppressed in vivo via the inclusion of a suicide gene in the CAR cassette [202]. Following CAR-T models [203], several NK cell product developers have included an inducible human caspase 9 transgene (iCasp9) in their CAR designs. The iCasp9 is fused to the human FK506-binding protein (FKBP), and administration of a small molecule, the chemical inducer of dimerization (CID) Rimiducid, results in dimerization and activation of the iCasp9-CID complex, which in turn activates the caspase signaling cascade leading to NK cell apoptosis [204, 205]. Anti-CD19 scFv-CAR-NK cells from MD Anderson and Takeda are equipped with iCasp9 (Additional file 2: Table S2, rows 12–13 and 16; Additional file 4: Table S4, row 11). Due to the absence of any serious toxic effects in treated patients, the safety switch has not been activated in humans [170]. The CD20 suicide gene system, based on the administration of Rituximab to eliminate CD20-transduced T cells in vivo upon insurgence of side effects [206, 207], has not yet been implemented in NK cells, but is a possibility. An alternative method to effectively eliminate infused NK cells upon the occurrence of serious side effects, without the need for genetic engineering, is to administer a therapeutic antibody directed against surface antigens endogenously present on NK cells. Although this approach has not yet been adopted in the clinic, the administration of Daratumumab to selectively kill CD38-expressing NK cells could represent a suitable option as a safety switch, although effects on the host NK cells should be evaluated.

#### Fratricide prevention

Expression of CAR target antigens on NK cell surface may cause self-killing (fratricide) of CAR-NK cells during cell manufacturing or in vivo. For example, CD38 is an established target for MM and is under investigation for AML, but its expression on NK cell surface poses a barrier to CD38 CAR-NK therapy development [208]. The hnCD16- and IL-15RF-engineered FT538 and the hnCD16-, IL-15RF-, and scFv-CAR-engineered FT576 products from Fate Therapeutics, modified to enhance ADCC in combination with anti-CD38 mAb Daratumumab and to improve persistence, are further engineered to knock-out CD38 via clustered regularly interspaced short palindromic repeats (CRISPR)-Cas9 and avoid Daratumumab-mediated fratricide (Additional file 2: Table S2, rows 24–27; Additional file 4: Table S4, rows 6–9). In vitro data from Woan et al. demonstrated a positive effect in ADCC upon knocking out CD38 in NK cells; however, they were unable to demonstrate the CD38 KO effect in a xenogeneic model [170, 198]. In the future, knock-out strategies could help expand the application of NK cell therapeutics to target antigens that are also expressed on mature NK cells or during certain developmental stages, especially in the case of HSC-derived or iPSC-derived NK cell products. Possible unforeseen effects of knock-out on NK cell development and maturation, however, should be carefully monitored during product development.

## Combination NK cell therapies

**Fig. 6:**
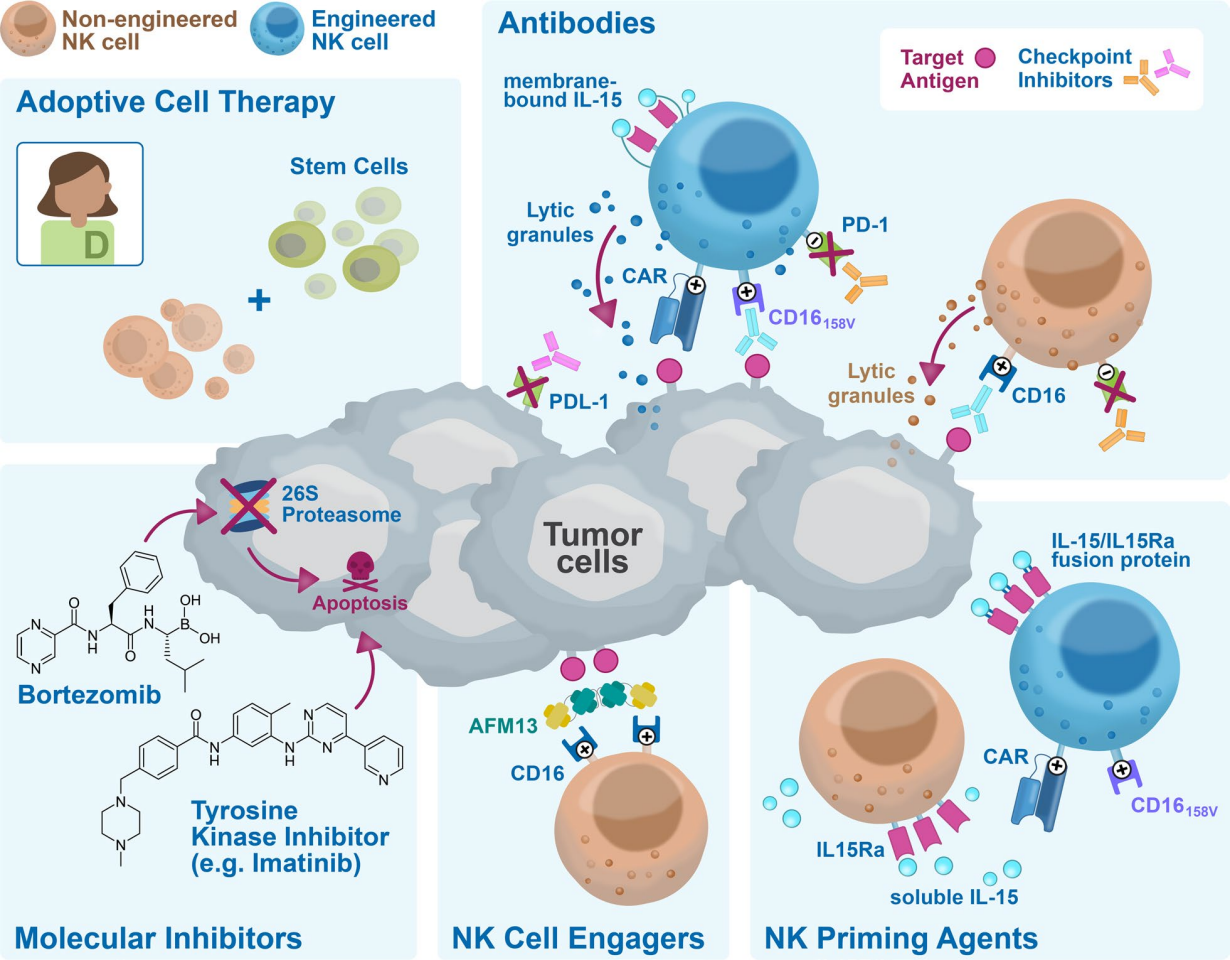
Overview of combination non-engineered and engineered NK cell therapies. From top left: in combination with adoptive cell therapy, NK cells are administered with stem cells from a healthy donor. Monoclonal antibodies are combined with NK cells to enhance tumor targeting via antibody-dependent cell cytotoxicity (ADCC) through endogenous CD16 or engineered high-affinity non-cleavable hnCD16 (158 V) on NK cell surface, or as checkpoint inhibitors to block inhibitory receptors on the surface of NK cells (such as programmed cell death protein 1, PD-1) or to block inhibitory ligands on the surface of target cancer cells (such as programmed death-ligand 1, PD-L1). NK cell priming agents, such as IL-15 or IL-15 and IL-15 receptor fusion protein IL-15/IL-15Ra, stimulate NK activation in vivo. NK cell engagers (NKCE) are synthetic molecules built from fragments of monoclonal antibodies engaging simultaneously a tumor-associated antigen (TAA) on the cancer cells and CD16 on the NK cells (e.g., AFM13). Molecular inhibitors act on target cancer cells, inducing apoptosis via inhibition of the proteasome (Bortezomib) or via inhibition of tyrosine kinases (e.g., Imatinib)

The rise of NK cell therapies has also spurred the development of novel combination approaches that aim to enhance functional aspects of the NK cells to achieve stronger and sustained anti-tumor responses [209]. Such functions include cell survival and activation, migration to the tumor site, resistance to the immunosuppressive TME, capacity to exert ADCC, and enhanced cytotoxic function through target immunomodulation. Clinical development of NK cell combination therapies employs diverse types of therapeutic agents, namely NK cell priming agents, adoptive cell therapy, antibodies, tyrosine kinase inhibitors, small-molecule inhibitors, priming agents, and NK cell engagers. Such approaches, pursued with both non-engineered and engineered NK cell products, are shown in Fig. 6 and described in detail in the following sections.

### Combination NK cell sources and clinical applications

**Fig. 7:**
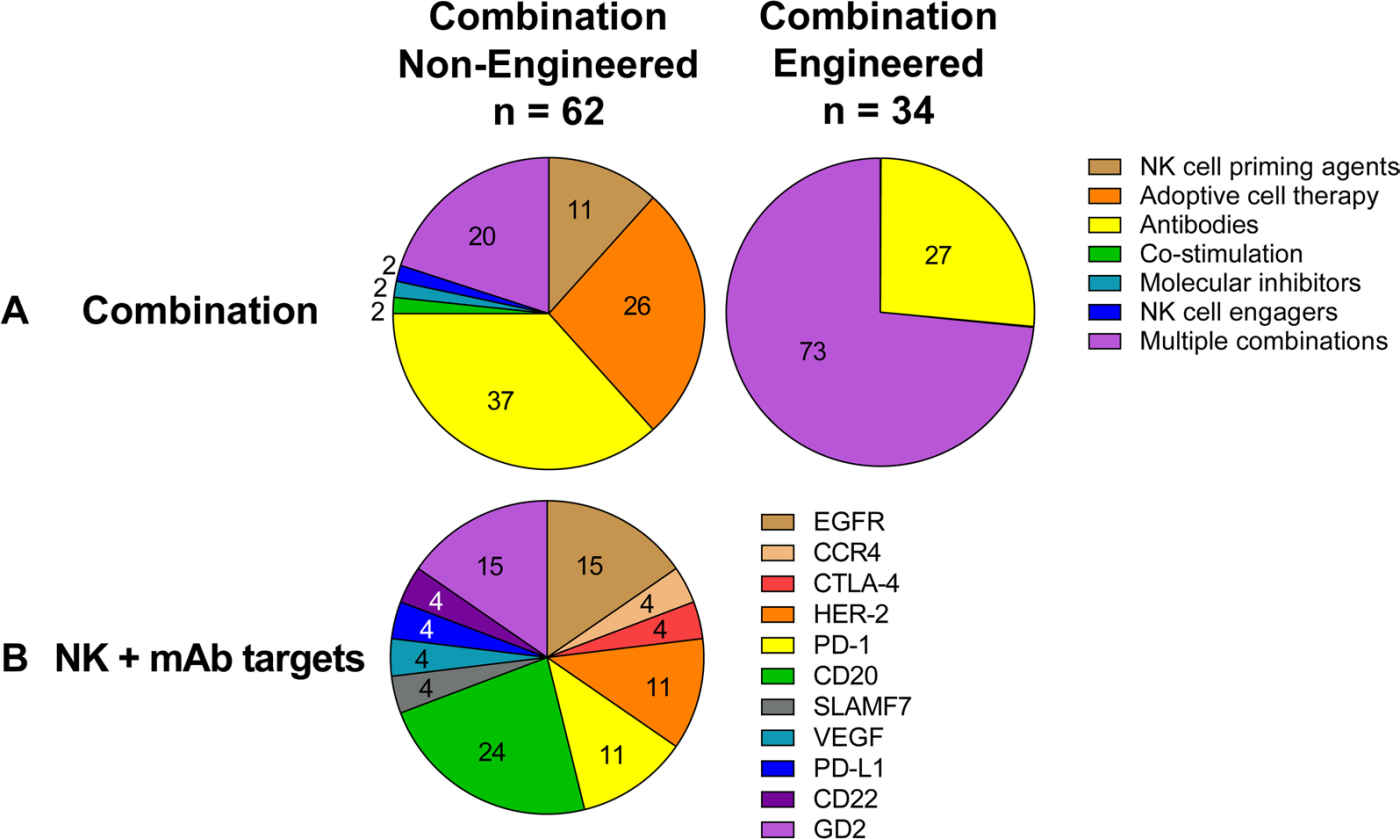
Statistics of combination non-engineered and engineered NK cell therapies. The pie charts present the types of combination therapies under clinical evaluation, as reported on ClinicalTrials.gov until December 2021, with non-engineered NK cells (*N* = 62 clinical trials) and with engineered NK cells (*N* = 34 trials). For every pie, numbers indicate the percentage of the whole taken by each slice. **A** Types of combinations with non-engineered and engineered NK cells: NK cell priming agents, adoptive cell therapy, antibodies, co-stimulation, molecular inhibitors, NK cell engagers; multiple combinations indicate the use of more than one of these categories. **B** Breakdown of the surface tumor antigens targeted by monoclonal antibodies used in combination with engineered and non-engineered NK cells. In B, on trial can have more than one target

The rapid growth of NK cell combination therapies is evident from the increasing number of products reaching the clinical stage. Until December 2021, a total of 62 and 34 clinical trials with allogeneic non-engineered or engineered NK cells have been listed on ClinicalTrials.gov, respectively; the type of combination agent, the features of the NK cell products, and the clinical trial details are provided in Additional file 3: Table S3 and Additional file 4: Table S4. Trials where the additional therapeutic agent was administered unrelated to the NK cell therapy or constitutes an ex vivo treatment of the NK cells prior to infusion have been excluded. Additionally, the landscape statistics for non-engineered and engineered NK combination therapies currently in clinical development are summarized in Figs. 2 and 7 and are described below. As shown in Fig. 2A, the most frequently used NK cell source for non-engineered NK in combination therapy is PB-NKs (72% of the trials), followed by UCB-NKs (17%), NK-92 (7%), while iPSCs only represent a small fraction (1%). In contrast, the most common source of engineered NK cells in combination therapy trials is NK-92 (71%), followed by iPSCs (26%), while UCB-NKs only make up a small proportion (3%). Figure 2B highlights the distribution of trials between hematological and solid malignancies. Interestingly, combination therapies utilizing non-engineered NK cells dominate in the field of hematological (62%) compared to solid (38%) malignancies, while the opposite is observed with engineered NK cells, leading against solid (76%) compared to hematological (24%) malignancies. Combination therapies within hematological malignancies (Fig. 2C) are equally distributed among AML and other unspecified leukemias for non-engineered NK (16%), followed by MDS (15%), NHL (12%) and other unspecified lymphomas (11%), chronic myelogenous leukemia (CML) (10%), Hodgkin lymphoma (HL) (7%), MM (5%), B cell lymphomas (4%), chronic lymphocytic leukemia (CLL) (3%) and B cell acute lymphocytic leukemia (B-ALL) (1%). Engineered NK are most used in B cell lymphomas (26%), followed by AML and MM (both at 20%), NHL (13%), and lastly in CLL, or in other unspecified leukemias or lymphomas (at 7%). Looking at solid tumors (Fig. 2D), non-engineered NK combinations focus on neuroblastoma (27%), followed by NSCLC, breast cancer, and CRC (11% each), next on pancreatic, Merkel cell, head and neck, and liver cancer (7% each), and finally on melanoma, biliary tract and renal cell cancer (4% each). Engineered NK combinations are primarily directed against pancreatic cancer (19%), followed by breast cancer (16%), glioblastoma, head and neck, and NSCLC (11% each), next on ovarian, colorectal and liver cancer (8% each), and lastly melanoma and renal cell cancer (4%).

The distribution of the types of combination therapies used together with non-engineered or engineered NK cells is reported in Fig. 7. As shown in Fig. 7A, antibodies represent the most frequent category of combination therapy used with non-engineered NK cells (37%), followed by adoptive cell therapy (26%), combinations of multiple agents (20%), NK cell priming agents (11%), costimulation (2%), molecular inhibitors (2%) and NK cell engagers (2%). In the case of engineered NK cells, most of the combination approaches employ the use of multiple combination agents (73%) and the remaining clinical trials utilize antibodies (27%). Many of the combination approaches aim to directly target TAAs with monoclonal antibodies to induce ADCC via endogenous CD16 via or high-affinity, non-cleavable CD16 on NK cell surface (as explained in the “Engineered NK cell therapies” section). Figure 7B shows the distribution of antibody targets under clinical development in combination with either non-engineered or engineered NK cells. The most common target is CD20 (24%) on B cell malignancies, followed by GD2 (15%) in neuroblastoma and EGFR (15%) on several solid malignancies such as NSCLC and gastrointestinal cancers, PD-1 (11%) on biliary tract and other malignant solid tumors, HER-2 (11%) on HER-2-positive solid tumors such as breast carcinomas, CCR4 (4%) in T cell leukemias and lymphomas, CTLA-4 (4%) in HNSCC, SLAMF7 (4%) on B cell malignancies, VEGF (4%) on malignant solid tumors, PD-L1 (4%) in advanced solid tumors and CD22 (4%) in pediatric leukemias. The most common antibody targets specifically combined with engineered hnCD16 NK cells are reported in Fig. 5C and described in the “Engineered NK cell therapies” section.

### Types of NK combination therapies

#### NK cell priming agents

The general mechanism of action (MoA) for NK cell priming agents relies on the provision of factors, such as cytokines IL-15 or IL-2 or their modified variants, to support the survival and activation of NK cells, as well as sustained cytotoxic function. The IL-2 diphtheria toxin conjugate Denileukin Diftitox (IL2DT), composed of the amino acid sequences for diphtheria toxin followed by truncated IL-2, is used to selectively deplete CD25-expressing Tregs and allow PB-NK cell expansion and in vivo function by the Masonic Cancer Center (Additional file 3: Table S3, row 1) [27]. The IL-15/IL-15Rα fusion protein ALT-803 (also known as N-803), an IL-15 superagonist, is used with non-engineered PB-NK cells (Additional file 3: Table S3, rows 2–4), as well as by ImmunityBio in combination with their NK-92-derived non-engineered product (Additional file 3: Table S3, row 5) and engineered products haNK (Additional file 4: Table S4, rows 14–31) and t-haNK (Additional file 4: Table S4, rows 32–34). The IL-2/TCR fusion protein ALT-801, which targets the P53(aa264-272)/HLA-A*0201 complex (Additional file 3: Table S3, row 6), and the fusion protein Hu14.8-IL2 which is comprised of one molecule of anti-GD2 humanized mAb fused to two IL-2 molecules (Additional file 3: Table S3, row 7), have been combined with the administration of PB-NK cells to stimulate NK cell function against p53- or GD2-expressing tumor cells.

#### Adoptive cell therapy

Adoptive cell therapy, typically in the context of hematopoietic stem cell transplantation (HSCT), is also used in combination with allogeneic NK cell administration. The donor origin of the allogeneic NK cells may be either the same or different from that of the allogeneic HSC graft. For the clinical trials reported in Additional file 3: Table S3 where NK cells are given as combination therapy with adoptive cell therapy, 7/16 use the same source for NK cells and HSCT, 7/16 do not specify the NK cell source, and 2/16 use a different NK cell source, i.e., off-the-shelf NK cells. The aim of the combination is to rescue/reconstitute the bone marrow after disease-eradicating myelosuppressive therapy, followed by NK cell infusion to decrease graft rejection, decrease disease relapse, decrease the risk for transplant-related toxicities (e.g., infections, GvHD) and enhance graft versus leukemia (GvL) effect [17, 210]. This approach is primarily investigated with non-engineered NK cell products in the context of hematological malignancies, in several Phase I/II studies with PB-NK cells (Additional file 3: Table S3, rows 8, 10–11, 13–14, 16–17, 19–23) or UCB-NK cells (Additional file 3: Table S3, rows 15, 18). A few trials explore the combination of adoptive cell therapy with PB-NK cells for solid tumors such as sarcomas, NSCLC, and brain and central nervous system tumors (Additional file 3: Table S3, rows 9, 12 and 16).

#### Antibodies

Co-administration with monoclonal antibodies (mAbs) is the most clinically explored combination option for non-engineered and engineered NK cell products. Antibodies can support NK cell function and tumor targeting in multiple ways; the two main categories are represented by therapeutic antibodies targeting specific tumor-associated antigens to stimulate ADCC via CD16, and by checkpoint inhibitors, which interfere with inhibitory signals to unleash NK killing potential.

The combination of antibodies against TAAs and NK cells exploits the therapeutic potential of ADCC either via the endogenously expressed, non-modified CD16, or via the high-affinity, non-cleavable version hnCD16 after genetic engineering of poorly expressing NK cells. Combinations of mAbs with non-engineered NK cells have been tested in Phase I and II studies with PB-NKs, UCB-NKs, iPSC NKs, and NK-92 cells (Additional file 3: Table S3, rows 24–46, 48, 50–59 and 62). Currently, the mAbs Cetuximab, Rituximab, Obinituzumab, Enoblituzumab, Daratuzumab, Trastuzumab, Elotuzumab, Epratuzumab, and Avelumab, all capable of inducing ADCC, are under clinical investigation in combination with both non-engineered and engineered NK cells (Additional file 2: Table S2, Additional file 3: Table S3 and Additional file 4: Table S4). hnCD16-NK cells are under clinical investigation in combination with multiple mAbs, making this the most common combination approach for engineered NK cell products. Fate Therapeutics has pioneered this field, with the development of the iPSC-derived NK product FT516 and its derivatives, FT596, FT538 and FT576 (Additional file 4: Table S4, rows 1–9) (see also “Engineered NK cell therapies” section).

Checkpoint inhibitors are under clinical investigation in combination with both non-engineered and engineered NK cells. Anti-PD-1 mAbs Pembrolizumab and Nivolumab are used to treat solid tumors in combination with non-engineered PB-NK cells (Additional file 3: Table S3, rows, 28, 34), with non-engineered iPSC NKs (Additional file 3: Table S3, row 37), engineered iPSC-derived NK product FT538 (Additional file 4: Table S4, row 8) and with ImmunityBio’s engineered t-haNK cells (Additional file 4: Table S4, rows 10 and 34). Another checkpoint inhibitor, anti-CTLA-4 mAb Ipilimumab, is used in combination with non-engineered allogeneic CIML NKs in head and neck squamous cell carcinoma (HNSCC) patients (Additional file 3: Table S3, row 26). Anti-PD-L1 mAb Avelumab, a checkpoint inhibitor also capable of inducing ADCC, is used in combination with engineered haNK and t-haNK cells (Additional file 4: Table S4, row 12–31 and 34) and engineered iPSC-derived NK products FT538 (Additional file 4: Table S4, row 8) and FT516 (Additional file 4: Table S4, row 2). Other anti-PD-L1 checkpoint inhibitors that are used in combination with engineered NK cells are Atezolizumab and Durvalumab, both used with ImmunityBio’s t-haNK (Additional file 4: Table S4, row 34) and Atezolizumab with the iPSC-derived NK product FT538 (Additional file 4: Table S4, row 8). Additionally, the anti-VEGF antibody Bevacizumab is used for immunomodulation of the tumor microenvironment in combination with PB-NKs (Additional file 3: Table S3, row 35), NK-92 aNK (Additional file 3: Table S3, row 52) and engineered NK-92 haNK (Additional file 4: Table S4, row 13, 14, 17–23, 26–28, 30 and 31).

#### Molecular inhibitors

Molecular inhibitors comprise a collection of therapeutic agents which display different MoAs. Tyrosine kinase inhibitors (TKI) are a group of pharmacologic agents that disrupt the signal transduction pathways of protein kinases, such as Apatinib (target: VEGFR2), Gefitinib (EGFR), Imatinib (Bcr-Abl), Dasatinib (Abl), Nilotinib (Bcr-Abl, c-Kit, and PDGF), Bosutinib (Bcr-Abl, Src, Lyn, and Hck), and Ponatinib (Abl). A recently initiated clinical trial by Kiadis Pharma evaluates the use of TKIs (Imatinib, Dasatinib, Nilotinib, Bosutinib, Ponatinib) against CML, in combination with KDS-1001 non-engineered PB-NKs (Additional file 3: Table S3, row 60). Another type of molecular inhibitor is the proteasome inhibitor Bortezomib, which has been explored in combination with non-engineered NK cells in MM patients (Additional file 3: Table S3, row 61).

#### NK cell engagers

A type of therapeutic agent which is still in the early stages of clinical evaluation is NK cell engagers (NKCE). The first product reaching the clinical stage is AFM13-NK by Affimed, i.e., non-engineered UCB-NK cells precomplexed with AFM13 tetravalent bispecific antibodies that target a TAA on the tumor cells and CD16a on the NΚ cells. This approach is being evaluated at the MD Anderson Cancer Center in a Phase I trial in patients with HL or NHL, targeting CD30 on the tumor cells (Additional file 3: Table S3, row 62). More recently, the NKp46/CD16-based NKCE IPH6101/SAR443579 from Innate Pharma has entered clinical development in collaboration with Sanofi, after showing promising anti-tumor activity in preclinical models [211, 212]. Targeting of NKp46 could further increase the specificity of engagement of NK cells versus other immune cell types in vivo.

#### Multiple combination therapies

To synergistically enhance the anti-tumor efficacy of NK cells in vivo, NK cell administration has been combined with more than one of the previously mentioned therapeutic approaches (antibodies, priming agents, adoptive cell therapy, or molecular inhibitors), also in co-treatment with immunomodulatory drugs such as Lenalidomide, which is known for having a direct anti-tumoral function, as well as an NK cell stimulatory effect [213, 214]. For non-engineered NK cells, the most common multiple combination therapy regimen consists of HSCT and treatment with a mAb, with the addition of at least one more agent, such as Lenalidomide, TKI, cytokines or priming agents, and chemotherapy (Additional file 3: Table S3, rows 48–59). Multiple combinations with engineered NK cells have been attempted with anti-CD19 CAR-NK cells, combined with Rituximab and HSCT (Additional file 4: Table S4, row 11), but the field is dominated by ImmunityBio, who combine priming agents such as N-803 and various mAbs with adenovirus- or yeast-derived tumor antigen fusion protein vaccines with haNK and t-haNK cells (Additional file 4: Table S4, rows 10, 12–34).

## Clinical status of NK cell therapies

Among the 36 clinical trials with non-engineered NK cells listed in Additional file 1: Table S1, 6 are reported on ClinicalTrials.gov as completed, 17 as recruiting, 3 as active but not recruiting, 1 as not yet recruiting, and 1 trial enrolling per invitation. The remaining trials have been terminated (3), withdrawn (2), or have unknown status (3); final or intermediate results have been published and made available for 8 of the trials. Of the 53 studies with engineered NK cells in Additional file 2: Table S2, 2 have been completed, 19 are recruiting patients, 5 are active and not recruiting, 3 are not yet recruiting, 1 is enrolling by invitation, whereas the other 23 were suspended (1), terminated (1), withdrawn (4), or have unknown status (17). Only 6 trials have reported (interim) clinical data. For non-engineered NK combination therapies, Additional file 3: Table S3 reports 62 trials, of which 25 completed trials, 12 recruiting, 1 not yet recruiting, 8 active not recruiting, and 7 terminated, 2 withdrawn, 1 suspended and 6 with unknown status. 28 studies have published results. Out of 34 trials for combination therapy with engineered NK cells in Additional file 4: Table S4, there are currently only 1 completed trial, 9 recruiting trials, and 5 active not recruiting trials. A substantial proportion of trials are terminated (1), withdrawn (14), or have unknown status (4). Results have been published for 4 trials.

From the currently available clinical outcome, allogeneic NK cell therapies demonstrate high versatility in application against diverse hematological and solid tumor indications, alone or in combination with several agents. Direct product comparisons are not straightforward, as cells are administered at various dose levels, ranging from few million to billions, as single or repeated doses and with different schemes. Pre-conditioning immunosuppressive regimens, aimed at limiting NK cell rejection from the host's immune system, are administered by means of different agents and clinical protocols, and can influence NK cell persistence, efficacy, and occurrence of side effects; however, their use is beyond the scope of this review and will not be discussed. Overall, allogeneic NK cells have proven an excellent safety profile, generally with only mild adverse effects reported. Dose-limiting toxicities, CRS, or GvHD are sporadic, often occurring when NK cells were administrated with multiple other agents. NK persistence is highly variable, ranging from a few days to over 2 months, and averaging approximately 7 days. Anti-tumor efficacy is observed in most of the studies, albeit to different degrees, with patients receiving NK cells often achieving partial or complete responses.

In the following sections, the available (interim) outcome of the trials from Additional file 1: Table S1, Additional file 2: Table S2, Additional file 3: Table S3 and Additional file 4: Table S4 is collected. Individual trial results are presented per product type and sponsor and, when possible, a comparison with preclinical data is discussed.

### Fate Therapeutics

With 5 products that have reached the clinical stage, Fate Therapeutics is one of the major contributors to the advancement of NK cell therapeutics. The first product, PB-NK-derived and non-engineered FATE-NK100, has been evaluated as a monotherapy in two Phase I studies, one for ovarian, fallopian tube, or primary peritoneal cancer (APOLLO study, NCT03213964, Additional file 1: Table S1, row 21) and one for AML (VOYAGE study, NCT03081780, Additional file 1: Table S1, row 22). As an outcome of both trials, the product was considered safe and provided clinical benefits in 3/9 ovarian cancer patients and complete remission in 3/6 AML patients, despite the limited persistence of the NK cells [215, 216]. These results are in line with the previously published preclinical data, where the in vitro cytotoxicity against the ovarian adenocarcinoma cell line SKOV-3 after expansion with GSK3B inhibitor CHIR99021 was around 30%, while cytotoxicity against K562 myeloid leukemia cells reached 80%. In NSG (NOD scid gamma) mice bearing SKOV-3 tumors, although in combination with Herceptin treatment, NK cells cultured for 7 days with CHIR99021 showed more consistent tumor control compared to NK cells expanded for 7 days in DMSO or grown overnight in vitro with IL-15 stimulation [77]. FATE-NK100 has also been evaluated for the treatment of EGFR^+^ and HER2^+^ solid tumors in combination with Cetuximab (anti-EGFR mAb) and Trastuzumab (anti-HER2 mAb) in the Phase I DIMENSION study (NCT03319459, Additional file 3: Table S3, row 36). Preliminary results report no dose-limiting toxicities observed in dose escalation studies; however, results on anti-tumor efficacy for the combination therapy trial are not yet available [217].

The FATE-NK100 program was stopped as the off-the-shelf iPSC-derived NK cell product FT500 entered clinical development in a Phase I trial in solid tumors (NCT03841110, Additional file 1: Table S1, row 36; Additional file 3: Table S3, row 37) [218]. Preliminary results presented at the Society for Immunotherapy of Cancer (SITC) congress in 2020 suggest that FT500 is safe and well tolerated. Patients were treated with up to six doses divided into two cycles of 3 once-weekly doses (1–3 × 10^8^ total cells) as monotherapy or in combination with one immune checkpoint inhibitor (Nivolumab, Pembrolizumab, or Atezolizumab). 15 patients completed the first cycle with three doses of 1 × 10^8^ cells and 11 patients completed the second cycle with three doses of 3 × 10^8^ cells. No treatment discontinuation due to adverse effects of the immunotherapy was required, but it was due to disease progression in all cases. Among 15 heavily pretreated patients, 11 showed the best overall response. The dose expansion stage with 3 once-weekly doses of FT500 at 3 × 10^8^ cells per dose is ongoing in up to 15 patients with NSCLC or classical HL [219].

The iPSC-NK platform established by Fate also led to the development of engineered products. FT516 CD16-engineered NK cells are modified to express the high-affinity, non-cleavable hnCD16 variant to enhance ADCC in combination with monoclonal antibodies. Preclinical in vivo data with patient-derived xenograft mouse models of human B cell lymphoma where FT516 was combined with anti-CD20 mAb led to significant tumor regression (171). FT516 is currently being investigated in an open-label, multi-dose Phase I clinical trial as monotherapy and in combination with anti-CD20 mAb Rituximab or Obinutuzumab for the treatment of advanced B cell lymphoma (NCT04023071, Additional file 2: Table S2, row 21; Additional file 4: Table S4, row 1) [220]. Interim clinical results for 18 patients in the dose escalation study (October 2021), were 4 patients received 9 × 10^7^ cells/dose, 7 patients 3 × 10^8^ cells/dose, and 7 patients 9 × 10^8^ cells/dose, showed no signs of toxicities or GvHD. Patients had received a median of 3.5 prior lines of therapy and a median of three prior lines containing CD20-targeted therapy. 10/18 patients treated were naïve to treatment with autologous CAR-T cell therapy and 8 patients were previously treated with autologous CAR-T cell therapy. Of the 10 naïve patients, 8 achieved an objective response (OR) from which 5/10 a complete response (CR), and 3/8 patients previously treated with CAR-T cell therapy achieved an OR and CR [220, 221]. FT516 is evaluated in two other Phase I trials, an open-label, multi-dose study in combination with Avelumab therapy (anti-PD-L1 mAb) in a range of solid tumor indications (NCT04551885, Additional file 2: Table S2 row 21; Additional file 4: Table S4, row 2) and in combination with Enoblituzumab, a mAb targeting B7-H3 in ovarian cancer (NCT04630769, Additional file 2: Table S2, row 22; Additional file 4: Table S4, row 3).

FT516 derivative products FT538, FT596, and FT576 have also reached the clinical stage. FT538, engineered to express hnCD16, IL-15, and KO for CD38 (hnCD16 + IL-15RF + CD38 KO), is tested in two Phase I studies in AML, MM, and monocytic leukemia (NCT04614636 and NCT04714372, Additional file 2: Table S2 rows 24–25; Additional file 4: Table S4 rows 6–7) and in solid tumors (NCT05069935, Additional file 2: Table S2 row 26; Additional file 4: Table S4 row 8). No results have been published yet. FT596, co-expressing hnCD16 and a CD19-scFv-CAR (hnCD16 + anti-CD19 + IL-15RF) showed potent multi-antigen targeting activity in combination with anti-CD20 mAb against both CD19^+^ and CD19^−^ tumors in preclinical in vivo studies, with enhanced tumor cell clearance by the multiplexed CAR compared to primary CD19-CAR-T cells [222]. FT596 is currently evaluated in a Phase I clinical trial (NCT04245722, Additional file 2: Table S2 row 19; Additional file 4: Table S4, row 4) in relapsed/refractory B-NHL and CLL in combination with anti-CD20 mAbs Rituximab or Obinutuzumab, and in a Phase I trial (NCT04555811, Tale 2, row 20; Additional file 4: Table S4, row 5) in combination with Rituximab for the treatment of NHL, DLBCL and high-grade B cell lymphoma. From NCT04245722, interim data presented at ASH 2021 included safety and efficacy data from patients in the three cohorts receiving 3 × 10^7^, 9 × 10^7^, 3 × 10^8^ cells. Patients had received a median of four prior lines of therapy and a median of two prior lines containing CD20-targeted therapy. A total of 24 patients (*n* = 12 in the monotherapy arm and *n* = 12 in the combination arm) were evaluable for efficacy. 18 patients (69%) achieved an OR, including 12 patients (46%) that achieved a CR following a single dose of FT596. Eight of these 24 patients were previously treated with autologous CD19-targeted CAR-T cell therapy; six achieved an OR (67%) with a single dose of FT596. Notably, in the third and fourth dose cohorts of the combination arm comprising a total of 12 patients, 9 patients (75%) achieved an OR, including 7 patients (58%) who achieved a complete response following a single dose of FT596. Durability response from 13 responding patients showed that 10 patients continued in ongoing response, including 3 patients in ongoing complete response at least 6 months from initiation of treatment, 2 patients reached 6 months in complete response and subsequently had disease progression, and 1 patient had disease progression prior to 6 months. The FT596 treatment regimens were well tolerated, including in those patients treated with a second, single-dose cycle. No dose-limiting toxicities and no other adverse effects, including GvHD, were observed. Three low-grade adverse events (two Grade 1, one Grade 2) of CRS were reported, which were of limited duration and resolved without intensive care treatment [223]. No results are available for NCT04555811. FT576, carrying a BCMA-scFv-CAR (hnCD16 + anti-BCMA + IL-15RF + CD38 KO), has entered a Phase I study in MM (NCT05182073, Additional file 2: Table S2, row 27; Additional file 4: Table S4, row 9), but results are not available.

### Celularity

Celularity Inc. has developed a pipeline for the generation of non-engineered NK cells from placenta and umbilical cord blood hematopoietic stem cells, with two clinical-grade products PNK-007 and CYNK-001. PNK-007 has been tested as monotherapy in a Phase I study in 10 AML patients, which proved the safety of the product (NCT02781467, Additional file 1: Table S1, row 30). A single infusion of PNK-007 cells in a dose escalation setting (1, 3, or 10 × 10^6^ cells/kg) was safe and well tolerated, with only one treatable CRS case for the highest dose level (10 × 10^6^ cells/kg). Only 2 of the 10 patients achieved clinical response. The overall survival (OS) was 2.4 months, with all patients dying during the follow-up period due to disease progression or AML complications [224]. Additional encouraging findings about the persistence of the NK cells in the blood and bone marrow for up to one month may suggest expansion and further maturation of PNK-007 after infusion [49]. The trial was terminated as a business decision. In another Phase I study, PNK-007 was administered in combination with autologous HSCT for the treatment of MM in 15 patients, 12 newly diagnosed and 3 with relapsed/refractory disease (NCT02955550, Additional file 1: Table S1, row 31; Additional file 3: Table S3, row 18). No dose-limiting toxicities were observed. From the newly diagnosed MM, 9 out of 12 patients developed minimal residual disease (MRD) at day 90. At 1 year, 6 out of 12 were MRD-negative, 2 were not evaluable, 2 refused evaluation, 1 did not convert to MRD^−^ and 1 was excluded from the study prior to MRD evaluation due to failure to respond to auto-HSCT. From the relapse/refractory MM, 1 out of 3 was MRD^−^ at day 90. At 1 year, 1/3 was MRD^−^, 1 out of 3 did not convert to MRD^−^ and 1 was not evaluated [225]. These results are in line with preclinical studies showing that PNK-007 exhibited up to 68% cytotoxicity against MM patient bone marrow samples in vitro. In addition, xenograft mice bearing RPMI8226 tumors showed significantly reduced tumor size when treated with PNK-007 cells compared to vehicle control. While none of the control mice survived longer than 20 days, almost 50% of the mice treated with two doses of PNK-007 were still alive after 60 days [226].

CYNK-001 cryopreserved product entered 3 Phase I studies in hematological malignancies and solid tumors (NCT04309084, NCT04310592, and NCT04489420, Additional file 1: Table S1, rows 28–29, 32). NCT04309084 is indicated as “active, not recruiting.” For the AML study NCT04310592, investigators reported that no dose-limiting toxicity has been observed and suggested a correlation of NK cell persistence 28 days after the first infusion with negative MRD [227]. Study NCT04489420 planned to recruit 36 patients with glioblastoma, astrocytoma, and other brain tumors, but was terminated due to business decision while this article was being drafted. On February 1, 2022, Celularity registered a new Phase I/IIa trial for the treatment of glioblastoma with intravenous and intratumor CYNK-001 infusions together with IL-2 support (NCT05218408, trial not reported in Additional file 1: Table S1).

### Gamida Cell

Gamida Cell has entered the clinical stage with a Phase I trial with their first PB-NK-derived product GDA-201, administered in combination with Elotuzumab (anti-SLAMF7 mAb) in MM patients or with Rituximab (anti-CD20 mAb) in NHL patients, to facilitate tumor targeting and ADCC (NCT03019666, Additional file 1: Table S1, row 12 and Additional file 3: Table S3, row 30). Study results were presented during the ASH meeting in 2020 and 2021. 20 NHL and 15 MM patients were divided over three cohorts of dose escalation between 2 and 26 × 10^7^ cells/kg of body weight. 16 patients received the maximum target dose (median dose 12.4 (range 2.0–26.0) × 10^7^ GDA-201 cells/kg). No dose-limiting toxicities such as neurotoxicity, CRS, GvHD, or aplasia were observed [228, 229]. In NHL, 13/19 patients showed CR and 1 had PR. Re-treatment of 4 NHL patients with GDA-201 in the absence of lymphodepletion deepened the response from partial response (PR) to CR in 2/4, suggesting that omission of chemotherapy treatment is feasible as well as opening the possibility to enhance anti-tumor response with multiple infusions [228]. The median duration of response was 16 months (range 5–36 months). At a median follow-up of 11 months (range 1–36 months), progression-free survival (PFS) at 1 and 2 years was estimated at 50% (27–69%) and 35% (14–58%). OS at 2 years was 78% (51–91%). Flow cytometry confirmed the persistence of donor NAM-NK in peripheral blood up to days 7–14 (day 7 range 2–92% GDA-201 cells), as well as enhanced in vivo proliferation (median Ki67 99%) [229]. In the preclinical data presented at ASH in 2017, a MM mouse model showed 100% survival when treated with nicotinamide-stimulated NK (NAM-NK) cells compared to Mock infused animals. In vitro data suggested up to 75% cytotoxicity capacity of NAM-NK cells against K562, compared to up to 45% killing of NK cells non-stimulated with NAM [230].

### Glycostem Therapeutics

Glycostem Therapeutics has developed a pipeline for the expansion and differentiation of UCB-derived HSCs into functional off-the-shelf NK cells. An early Phase I clinical trial in AML patients, performed in collaboration with Radboud University, showed that treatment was well tolerated, and 2 of 4 patients with MRD in bone marrow before infusion became MRD-negative (< 0.1%) for 6 months [44]. During the ASH 2021 and EHA 2022 meetings, Glycostem presented initial data of their Phase I/II dose escalation and expansion study with cryopreserved GTA002 [39] in AML patients in morphologic complete remission with MRD who are not proceeding to allogeneic HSCT (WiNK study, NCT04632316, Additional file 1: Table S1, row 27). Six patients have been dosed with one (3 patients) and two (3 patients) single infusion(s) of up to 1 × 10^9^ GTA002 cells. MRD analysis was available for the first 3 patients and safety information for all 6. Periods of MRD negativity could be documented in the first three patients by multiparameter flow cytometry (MFC) and next-generation sequencing (NGS), with GTA002 NK cells being detected by chimerism analysis in peripheral blood and bone marrow up to 4 weeks after infusion. No GTA002-related safety signal was reported in any of the patients [231, 232].

### ImmunityBio

ImmunityBio leads the development of NK-92 cell line-derived immunotherapies. Non-engineered, activated NK-92 (aNK) cells have been tested in the QUILT-3.009 study against stage III and IV MCC in combination with the IL-15/IL-15Rα fusion protein ALT-803 (Phase II study NCT02465957, Additional file 3: Table S3, row 5). Interim data report that the treatment has been well tolerated in the initial 3 patients with advanced MCC. Promising partial response with > 70% regression ongoing at 20 weeks was seen in one patient refractory to multiple prior therapies, including PD-1 blockade. Changes were observed clinically in another patient’s superficial tumors a few hours after aNK infusions; however, the patient developed progressive disease (PD) at 4 weeks [233]. aNK cells have also been used in pancreatic cancer patients in combination with multiple chemotherapeutics, and with ALT-803 (NCT03136406, Additional file 3: Table S3, row 52). The trial status is unknown, and no data is reported.

Immunity Bio’s NK-92-derived product haNK, genetically engineered to express hnCD16 and ER-IL2, has entered multiple clinical trials either as monotherapy (Additional file 2: Table S2, rows 44–50) or in combination with monoclonal antibodies and other factors (Additional file 4: Table S4, rows 12–31). The QUILT-3.067 clinical trial (Phase I/II, NCT03387085, Additional file 4: Table S4, row 17) is the only one with available results. haNK cells were investigated as a treatment for triple-negative breast cancer (TNBC) in combination with the IL-15/IL-15Ra fusion protein N-803 and several other drugs. Preliminary results show that 2 out of 9 patients had grade > 3 adverse effects; however, NK cell therapy was administered with a combination of other treatments and no conclusion can be drawn on the safety of haNK cells. Nevertheless, OR was observed in 7 out of 9 patients, CR was achieved in 2 out of 9, and the duration of the response ranged between 2 and 12 months [234].

haNK-derivative product t-haNK, modified with additional engineering with a scFv-CAR, is also investigated in multiple trials as monotherapy (Additional file 2: Table S2, rows 38–39) or in combination (Additional file 2: Table S2, rows 40–43; Additional file 4: Table S4, rows 10, 32–34). PD-L1-scFv-CAR t-haNK is currently in clinical investigation in the Phase II QUILT-88 study for the treatment of pancreatic cancer in combination with N-803 and several other drugs (NCT04390399, Additional file 2: Table S2, row 41; Additional file 4: Table S4, row 32). Overall survival for the 61 patients in the 3rd, 4th, and 5th lines was reported to be 5.8 months, while the 30 patients in the 3rd line had OS of 6.3 months, with serious adverse effects being rare and no treatment-related deaths reported [235]. The same product is under assessment in combination with Pembrolizumab or Nivolumab (anti-PD-1 mAbs), Durvalumab, Atezolizumab or Avelumab (anti-PD-L1 mAbs) for the treatment of several solid malignancies (including NSCLC, small-cell lung cancer (SCLC), urothelial carcinoma, HNSCC, MCC, melanoma, RCC, gastric cancer, cervical cancer, HCC, and CRC) in the Phase II QUILT-3.055 study (NCT03228667, Additional file 2: Table S2, row 42; Additional file 4: Table S4, row 34). So far, preliminary results are available only for the non-NK cell-treated cohorts.

### MD Anderson Cancer Center

The University of Texas MD Anderson Cancer Center is developing multiple non-engineered, engineered, and combination NK cell therapies which have reached the clinical stage. Together with Sanofi and Kiadis Pharma, K-NK003 non-engineered PB-NK cells are evaluated in combination with stem cell transplantation in a Phase I/II study in AML, MDS, and CML (NCT05115630, Additional file 1: Table S1, row 5; Additional file 3: Table S3, row 23). Another non-engineered product, based on UCB-NK cells, is used for the treatment of solid tumors in a Phase I study (NCT03420963, Additional file 1: Table S1, row 34), which has no published results. scFv-engineered UCB-NK cells have entered four Phase I/II studies, two with anti-CD19 CAR-NK cells against B cell lymphoma (NCT03056339 and NCT03579927, Additional file 2: Table S2, rows 12–13), one with anti-CD5 CAR-NK against multiple hematological malignancies (NCT05110742, Additional file 2: Table S2, row 14), and one with anti-CD70 CAR (NCT05092451, Additional file 2: Table S2, row 15). Interim results are only available for NCT03056339. In this study, 11 patients were treated with CAR-NK cells directed against CD19, expressing anti-CD19 CAR, IL-15 and inducible caspase 9 suicide gene (iCasp9/CAR.19/IL-15). Patients received lymphodepleting chemotherapy (Cy/Flu) followed by a single infusion of CAR-NK cells of either 1 × 10^5^, 1 × 10^6^ or 1 × 10^7^ cells/kg body weight. Data show that no GvHD or severe CRS or neurotoxicity was reported. With a median follow-up of 13.8 months, 8/11 patients had an OR, from which 7/11 had a CR. Responses were difficult to assess, since 5/8 patients that responded received post- remission therapy after day 30 of assessment [170]. Preclinical data by Rezvani et al. in NSG Raji mouse models demonstrated in vivo homing, proliferation, and anti-tumor activity of the CAR-NK cells, especially if co-expressing IL-15 [145]. Mice that received two doses of iCasp9/CAR.19/IL-15 CAR-NK cells showed detectable numbers of CAR-NK cells up to 68 days post-infusion; however, infusion of higher numbers of CAR-NK cells was associated with toxicities and death, highlighting the importance of controlling NK cell survival by the presence of a suicide gene [145].

In collaboration with Altor BioScience, allogeneic PB-NK cells were combined with the NK priming ALT-801 IL-2/TCR fusion protein against AML (NCT01478074, Additional file 3: Table S3, row 6), but the trial was withdrawn due to low feasibility. Allo-PB-NK were also combined with allo-HSCT in Phase I/II studies in hematological malignancies (NCT01823198 and NCT01904136, Additional file 3: Table S3, rows 10 and 14, respectively). From study NCT01904136, investigators concluded that donor-derived mb-IL21 ex vivo expanded NK cells contributed to a decreased post-transplant relapse rate in 25 patients receiving haploidentical HSCT. NK cells were administered at 3 doses (1 × 10^5^–1 × 10^8^ cells/kg/dose) on days − 2, +7, and +28. The 2-year relapse rate was 4% versus 38% and the disease-free survival (DFS) was 66% versus 44% in patients treated with HSCT and NK cells compared to patients that only received HSCT. The treatment was deemed safe and showed signs of early NK cell immune reconstitution after HSCT, preserved T cell reconstitution, and better relapse and DFS [236].

Combination therapies with mAbs have been developed for non-engineered cord blood NK cells in CRC with Cetuximab (anti-EGFR mAb, NCT05040568, Additional file 3: Table S3, row 24) and with PB-NK cells in pediatric leukemia with Epratuzumab (anti-CD22 mAb, NCT00941928, Additional file 3: Table S3, row 39). No results have been published. Trials with NK combinations with multiple agents have been initiated with non-engineered UCB-NK, PB-NK, and NK-92 cells (NCT02280525, NCT00383994, NCT02727803, NCT01619761, NCT03019640 and NCT01729091, Additional file 3: Table S3, rows 48, 51, 53–55, 58). In NCT03019640, the use of ex vivo expanded UCB-NK cells in combination with autologous HSCT, Lenalidomide, Rituximab (anti-CD20 mAb), and chemotherapy for the treatment of recurrent or refractory B cell NHL was evaluated. Preliminary results from 19 treated patients showed CR for 78.9%, PR for 15.8%, and PD for 5.3%. UCB-NK cells were detectable in the patients’ blood for an average of 2 weeks. At a median 18-month follow-up, the relapse-free survival (RFS) rate was 68% and OS rate was 84% [237]. No result is available from the other trials. Multiple combinations of iCasp9/CAR.19/IL-15 engineered UCB-NK cells with anti-CD20 Rituximab and autologous HSCT in B cell lymphoma trial (NCT03579927, Additional file 2: Table S2, row 13; Additional file 4: Table S4, row 11) were withdrawn for lack of funding. UCB-NK cells precomplexed with the AFM13 CD16a/CD30 bispecific NK cell engager are currently tested within a Phase I/II study in collaboration with Affimed (NCT04074746, Additional file 3: Table S3, row 62). Among 13 patients treated at the recommended phase 2 dose (10^8^ NK/kg), 8 patients (62%) had CR after two cycles of treatment, which represents an increase from 5 patients (38%) showing CR after one cycle of treatment. The median duration of response has not yet been reached. Seven patients remain in CR at a median follow-up of 6.5 months, including 2 patients after 10 months and 2 who received stem cell transplant and remain in response at 6.5 months. One patient with a CR experienced disease progression after 7.9 months. Of the 5 patients with a PR, one remains in response at 6.3 months and four patients progressed between 2.9 and 4.3 months after initial infusion [238].

### Sanofi/Kiadis Pharma

Kiadis Pharma (now Sanofi) has developed two non-engineered PB-NK products, K-NK002 and K-NK003. A Phase II study (NCT04395092, Additional file 1: Table S1, row 4; Additional file 3: Table S3, row 22) with K-NK002 haploidentical NK cells to prevent post-transplant relapse in patients with AML and MDS was withdrawn as a decision from the sponsor. Off-the-shelf K-NK003 (also known as KDS-1001) cells, manufactured using Kiadis’ FC21 and proprietary universal donor platforms [107, 239] have entered three trials against hematological malignancies, a Phase I/II study in collaboration with MD Anderson Cancer Center, in combination with stem cell transplantation (NCT05115630, Additional file 1: Table S1, row 5; Additional file 3: Table S3, row 23, see also “MD Anderson Cancer Center” section), a Phase I study with the National Cancer Institute (NCI) and the Ohio State University (NCT04220684, Additional file 1: Table S1, row 6), and a Phase I study with Duke University in combination with tyrosine kinase inhibitors (NCT04808115, Additional file 3: Table S3, row 60). No data is available from any of the studies.

### Fuda Cancer Hospital and Shenzhen Hank Bioengineering Institute

The allogeneic PB-NK-derived, Highly Activated NK cells (HANK cells), from the Fuda Cancer Hospital of Guangzhou in collaboration with Shenzhen Hank Bioengineering Institute have been clinically tested in 20 patients with recurrent liver carcinoma (NCT03008343, Additional file 1: Table S1, row 24). This Phase I/II clinical trial aimed to evaluate whether the combination of irreversible electroporation (IRE) with NK cell adoptive therapy resulted in better clinical outcomes compared to IRE only. Stage IV HCC patients received HANK cells 4–6 days after IRE, or only IRE. IRE-NK patients tolerated the treatment well and showed less circulating tumor cells 7 and 30 days after treatment and a significant (*P* < 0.01) tumor volume 3 months after treatment. Six patients in the IRE group and 2 patients in the IRE-NK group had PD. The disease control rate was 90% in the IRE-NK group and 75% in the IRE group. The IRE-NK group had significantly longer OS than the IRE group (*P* < 0.01) [83].

Additionally, the two institutes registered five Phase I/II clinical trials with donor-derived PB-NK cells in combination with antibodies, with the purpose to enhance ADCC, for checkpoint inhibition, or for immunomodulation of the TME (Additional file 3: Table S3, rows 31–35). The trial outcome is available for two studies. In the combination of Cetuximab (anti-EGFR mAb) and NK cell immunotherapy for recurrent NSCLC (NCT02845856, Additional file 3: Table S3, row 33), patients were randomized into two groups, with group A receiving allogeneic PB-NK cells activated with lethally irradiated K562-mbIL15-41BBL cells in combination with Cetuximab, while group B only receiving Cetuximab. Overall, the median PFS and OS for group A were longer, with 6 and 9.5 months respectively, compared to group B, with 4.5 and 7.5 months. Importantly, the NK cell plus Cetuximab group exhibited an enhanced immunological response compared to the Cetuximab-only group, while NK cells were significantly increased in both groups after treatment. While the study’s limited sample size did not allow adequate demonstration of clinical benefit, the positive survival results would encourage a long-term assessment of this combination therapy in a larger cohort [240]. Similarly, PB-NK cells were tested in combination with anti-PD-1 mAb Pembrolizumab for recurrent NSCLC (NCT02843204, Additional file 3: Table S3, row 34). The NK plus Pembrolizumab group (A) had longer survival than patients only receiving Pembrolizumab (group B), with a median OS of 15.5 months vs 13.3 months and median PFS of 6.5 months vs 4.3 months. Moreover, the patients in group A treated with multiple courses of NK cell infusion had better OS (18.5 months) than those who received a single course of NK cell infusion (13.5 months). In conclusion, Pembrolizumab plus NK cell therapy yielded improved survival benefits in patients with previously treated PD-L1^+^ advanced NSCLC [241].

### The Third Affiliated Hospital of Guangzhou Medical University

In a pilot Phase I clinical trial from The Third Affiliated Hospital of Guangzhou Medical University (NCT03415100, Additional file 2: Table S2, row 1), 3 patients with metastatic solid tumors received multiple rounds of intraperitoneal infusions or intratumoral injections of either autologous (*n* = 1) or haploidentical (*n* = 2) NKG2D receptor-engineered CAR-NK cells. Supporting in vitro data showed that NKG2D-CAR-NK cells showed around 60% cytotoxicity against HCT116 in an in vitro assay at an E/T ratio of 10:1 on the day of infusion. Additionally, proof of concept data for in vivo tumor killing was established in a xenograft NSG mouse model, injected with HCT116-Luc colorectal cancer cells. The tumor-bearing mice were treated with multiple rounds of PBS, Mock NK cells, or NKG2D-CAR-NK cells (1 × 10^7^ cells/injection). The tumor burden was reduced in mice receiving NKG2D-CAR-NK cells compared to the two control groups [169]. In humans, no toxicities or GvHD were reported after NK cell treatment. Positron emission tomography and computed tomography (PET-CT) scans of the CAR-NK injection site showed a reduction in the number of tumor cells, or even complete metabolic response (CMR) in one patient. Due to the transient CAR expression associated with mRNA electroporation, multiple rounds of treatment were necessary to achieve anti-tumor effects [169]. The pilot study was extended to a total of *n* = 30 patients, but results have not yet been published.

### PersonGen BioTherapeutics

Anti-CD33 scFv-CAR NK-92 cells from PersonGen BioTherapeutics have entered a Phase I/II clinical trial for relapsed and refractory AML patients (NCT02944162, Additional file 2: Table S2, row 30). The interim results from at least 3 patients demonstrated for the first time the safety and efficacy of CAR-NK-92 cells in the treatment of relapsed and refractory AML. Although CAR-engineered cells were not potent enough in patients with high tumor burden, the findings from this clinical study provided evidence that CAR-NK-92 could be safely infused (up to 5 × 10^9^ cells per patient) without any grade 3–4 adverse side effects [172]. Anti-MUC1, anti-CD19, and anti-CD7 CAR-NK products are under evaluation in three clinical studies (NCT02839954, Additional file 2: Table S2, row 28; NCT02892695, Additional file 2: Table S2, row 32; NCT02742727, Additional file 2: Table S2, row 33), but no data has been published.

### Masonic Cancer Center

The Masonic Cancer Center from the University of Minnesota has participated in the clinical development of multiple NK cell products in cooperation with private sponsors as Gamida Cell (Additional file 1: Table S1, row 12), Miltenyi Biotech (Additional file 1: Table S1, row 13) and Fate Therapeutics (Additional file 1: Table S1, rows 21–22; Additional file 2: Table S2, rows 20, 23–24; Additional file 4: Table S4, rows 3,5, and 7). Additionally, they have sponsored two clinical trials with allo-PB-NK combination with priming agents for AML (Additional file 3: Table S3, rows 1 and 3), two with antibody combination for CLL and NHL (Additional file 3: Table S3, rows 40–41), one with antibody combination in hematological malignancies (NCT03019666, Additional file 3: Table S3, row 30) and one study with the combination of umbilical cord blood transplantation and UCB-NK administration (Additional file 3: Table S3, row 15).

In the Phase II study NCT01106950 (Additional file 3: Table S3, row 1), 15 AML patients received IL-2 every other day (for a total of 6 doses) after infusion with donor-derived PB-NK cells and with IL-2 diphtheria toxin (IL2DT, Ontak). Augmented lymphocyte and Treg depletion with IL2DT resulted in remission at day 28 for 8 of 15 patients (53%), including CR (*n* = 3). Six patients in remission subsequently received allogeneic donor HCT 45 to 120 days after NK cell therapy. One transplant-ineligible patient remained in CR until relapse at 8 months, and 1 patient who successfully expanded donor NK cells and cleared leukemia died of neutropenic sepsis at day 30. The median duration of remission was 11.2 months (range, 1–32 months). Successful donor NK cell expansion was observed in 4/15 (27%) of patients. Median absolute circulating donor-derived NK cell counts at day 14 were 1000 NK cells/µl (range, 480–12 390 cells/µl; *P* = 0.12). A strong inverse correlation between day 7 absolute PB Treg count, and successful in vivo donor NK cell expansion was observed. Of the 6 patients with < 5% Tregs at day 7 (median absolute Treg of 3 cells/µl; range, 0–6 cells/µl), 5 successfully expanded donor NK cells. None of the 9 patients with higher Treg counts expanded NK cells (median Treg, 32; range, 10–69 cells/µl; *P* < 0.01) [27]. The trial was terminated due to the study drug Ontak being no longer available. The QUILT-3.033 Phase II clinical trial (NCT03050216, Additional file 3: Table S3, row 3) treated relapsed or refractory AML patients with haploidentical donor non-modified PB-NK in combination with subcutaneous administration of ALT-803 IL-15/IL-15Ra fusion protein. Out of 7 evaluable patients, 6 died within 6 months of treatment and all patients died within a year. Only 1 patient (14.3%) exhibited complete remission at 42 days after infusion. No signs of successful in vivo NK cell expansion were observed in any patient [96].

A combination of allogeneic NK cell therapy with antibodies was evaluated in two trials with Rituximab (anti-CD20 mAb). In the first clinical trial (Phase I/II, NCT00625729, Additional file 3: Table S3, row 40), out of 6 evaluable patients, none exhibited donor NK cell expansion at day 14 post-infusion. The overall response at 3 months post-infusion was seen in 4 out of 6 patients, and from those 4 responding patients 2 showed disease progression at 6 months after treatment. Overall survival rate at 6 months post treatment was 50%. The study was terminated, as no patients exhibited NK cell expansion. The other study (Phase II, NCT01181258, Additional file 3: Table S3, row 41) investigated the efficacy of the combination of allo-PB-NK cell therapy with Rituximab in refractory NHL. Treatment was well tolerated, with no GvHD, CRS or neurotoxicity. Of 15 evaluable patients, 4 had objective responses (26.6%) at 2 months; 2 had complete responses lasting 3 and 9 months. Circulating donor NK cells were detectable up to 7 days post-infusion and responding patients had lower levels of circulating T regulatory and myeloid-derived suppressor cells, compared to non-responders [242]. In another study in collaboration with Gamida Cell, the anti-SLAMF7 mAb Elotuzumab is administered to MM patients, and Rituximab to NHL patients (Phase I, NCT03019666, Additional file 3: Table S3, row 30). Results have been published and discussed under “Gamida Cell.”

A combination of umbilical cord blood transplantation along with UCB-NK cells was tested for the treatment of myeloid leukemia patients that were not in CR (NCT00354172, Additional file 3: Table S3, row 15). Although the trial was terminated due to the initiation of a competing study, initial results showed that 15 out of 16 patients completed the trial and 2 out of the 15 patients were disease-free and alive for 6 months after treatment with UCB-derived NK cell infusions.

### Asan Medical Center

The Asan Medical Center in Seoul assessed the efficacy of donor NK cell infusion given after HLA-mismatched/haploidentical allogeneic HSCT in patients with advanced malignant disorders (Phase I/II, NCT00823524, Additional file 3: Table S3, row 16). No acute toxicity was observed after NK cell infusion. A significant reduction in leukemia progression (74–46%) with post-transplantation NK cell infusion was found as an independent predictor for less leukemia progression [25]. Another study (Phase I/II, NCT01795378, Additional file 3: Table S3, row 17) assessed the efficacy of donor NK cell infusion given after HLA-mismatched HSCT in patients with refractory acute leukemia. 51 patients with AML or ALL received NK cells on days 6, 9, 13, and 20 after HSCT. Median NK cell doses were 0.5, 0.5, 1, and 2 × 10^8^/kg cells, respectively. During NK cell treatment, 33/45 evaluated patients (73%) developed treatment-related toxicities, which were reversible in 90% of the cases. The leukemia complete remission rate was 57% at 1 month after HSCT, and the 3-year cumulative incidence of leukemia progression was 75%. Additional donor NK infusions on days 6 and 9 were not associated with less leukemia progression (75% vs. 55%) [243]. A similar Phase II study, in combination with HLA-haploidentical HSCT, was terminated after recommendation from the data monitoring committee (NCT02477787, Additional file 3: Table S3, row 21). Another study, for PB-NK cell infusion in 5 patients with NSCLC, was completed but no results were found (NCT03366064, Additional file 1: Table S1, row 23).

### GC Pharma/Green Cross LabCell

GC Pharma, with the Seoul National University Hospital, has initiated a Phase II trial with their allogeneic PB-NK cell product MG4101 in 13 AML patients (NCT03349502 Additional file 1: Table S1, row 14). No results are available. Together with the Asan Medical Center, MG4101 cells entered another Phase I/II trial, in combination with the anti-CD20 antibody Rituximab against NHL (NCT03778619, Additional file 3: Table S3, row 29). Of the 9 patients treated, the OR rate was 55.6%, with 5/9 partial responses. MG4101 persisted for up to 14 days. No GvHD was observed [244]. These studies are a follow-up of the first-in-human use of MG4101 for the treatment of malignant lymphomas and solid tumors (NCT01212341, 2010–2013, not reported in Additional file 1: Table S1).

### St. Jude Children's Research Hospital

Following early studies with haploidentical NK cell transplantation in pediatric AML [19], the St. Jude Children's Research Hospital has recently entered clinical trials with several NK cell products. CD19 scFv-CAR-engineered PB-NK cells have been investigated against B-ALL (NCT00995137, Additional file 2: Table S2, row 10), but no result has been posted after recruitment was completed. Non-engineered PB-NK cells have been combined with donor hematopoietic progenitor cells in leukemia and lymphoma patients (NCT01807611, Additional file 3: Table S3, row 8); recruitment has been completed, with no results yet. The use of T cell replete haploidentical donor hematopoietic stem cell transplantation in combination with NK cell transplantation for the treatment of relapsed or refractory hematological malignancies despite previous allogeneic transplant was also evaluated (NCT01621477, Additional file 3: Table S3, row 20). The trial, which was terminated due to replacement, showed 7 out of 17 treated patients alive 1 year after trial initiation. 10 patients had malignant relapse 10 months after transplantation. No acute GvHD was observed in 9 patients, Grade I GvHD was seen in 3 patients, Grade II in 1 patient, Grade III in 3 patients, and Grade IV in 1 patient. Two patients died due to transplant-related mortality.

Combination or PB-NKs with anti-GD2 mAb (HU14.18K322A) was pursued for pediatric patients with neuroblastoma (NCT01576692, Additional file 3: Table S3, row 45). Patients were heavily pre-treated (9/13 with prior anti-GD2 therapy) and received parent-derived NK cells with anti-GD2 and various chemotherapies. One patient developed an unacceptable toxicity (grade 4 thrombocytopenia > 35 days), while 4 patients discontinued treatment for adverse events (hu14.18K322A allergic reaction, viral infection, surgical death, second malignancy). Common toxicities included grade 3/4 myelosuppression (13/13 patients) and grade 1/2 pain (13/13 patients). The response rate was 61.5% (4 complete responses, 1 very good partial response, 3 partial responses, and 5 disease stabilizations). The median time to disease progression was 274 days and 77% of patients survived over 1 year. Importantly, the contribution of NK cells to the clinical responses cannot be clearly distinguished, as they were administered together with multiple other agents [245].

NK combinations with multiple other agents were assessed in three studies (Additional file 3: Table S3, rows 49–50, 56). In one study, the effect of NK cell transplantation with HLA-non-identical stem cell therapy, OKT3 (anti-CD3 mAb) treatment, and chemotherapy was investigated in pediatric hematological malignancies (NCT00145626, Additional file 3: Table S3, row 49). The primary objective of the NK cell infusion was to decrease the risk of graft rejection and disease relapse and to reduce severe transplant-related toxicities, including infection and GvHD. Although 40 patients were planned, only 19 were recruited and 14 completed the trial. The primary endpoint of this study was to assess survival at 1 year post treatment, at which 50% of the patients were alive. Regimen-related mortality was observed in 3/14 patients at 100 days post-transplantation. No incidence of fatal GvHD was observed at 100 days PT, while 1 patient had limited chronic GvHD, and 13 patients showed no signs of chronic GvHD when assessed up to 5 years post treatment. At year 1 post treatment, 6 patients were in complete remission and 1 had progressive disease from the group of 7 patients that survived at least one year post HSCT. Among the 7 patients in the group that did not survive for at least one year from HSCT, 1 patient had active disease, 2 complete remission-1, 1 complete remission-2, and 3 relapsed [67]. In another study, a combination of allogeneic NK cells with anti-GD2 mAb (hu14.18K322A), peripheral blood stem cell transplantation, and cytokines IL-2, G-CSF, GM-CSF was administered to pediatric neuroblastoma patients (NCT01857934, Additional file 3: Table S3, row 56). 30 patients received stem cell transplantation with NK cells; 21 patients received haploidentical NKs at a median of 4 days (range, 3–5 days) post-HPC infusion. The infused NK products contained a median of 27.6 × 10^6^ cells/kg CD56^+^ cells (range, 4.1–113.979 × 10^6^ cells/kg) and 0.01 × 10^6^ cells/kg CD3^+^ cells (range, 0.003–0.104 × 10^6^ cells/kg). All 21 patients had at least 1 peripheral blood sample collected and sorted for NK chimerism testing. Twenty patients had a sample collected at the first time point (median 11 days post-HPC infusion; range 8–14), 15 of which had sufficient NK cells for testing. Of these, 14 samples had detectable donor DNA (median 3.5% donor DNA; range, 0–68%). 18 patients had a sample collected at the second time point (median 25 days post-HPC infusion; range 22–50), all of which were adequate for testing. Of these NK cell samples, 6 contained detectable donor DNA (median donor DNA 1%; range, 0–35%). Toxicities were similar for patients that did or did not receive NKs. No patients developed acute GvHD [246]. In another trial, PB-NKs with CD133^+^ autologous HSCT, hu14.18K322A anti-GD2 Ab, IL-2, G-CSF, and GM-CSF were evaluated in children with high-risk solid tumors (NCT02130869, Additional file 3: Table S3, row 50). No data has been published.

### Others

The Washington University School of Medicine combined allogeneic PB-NK treatment with IL-2 or ALT-803 IL-15/IL-15Ra fusion protein in AML and MDS patients (NCT01898793, Additional file 3: Table S3, row 2). The trial is currently not recruiting. Notably, two independent studies treating leukemia patients with NK cells and cytokine support by systemic IL-15 (Additional file 3: Table S3, rows 2 and 3, see also “Masonic Cancer Center”) resulted both in reduced clinical activity, possibly by promoting T cell activation and consequent acceleration of NK cell rejection [96].

The National Cancer Institute investigated IL-15/4-1BBL-activated allo-PB-NK cell infusions following allogeneic T cell-depleted peripheral blood stem cell transplantation in 34 pediatric patients with solid tumors and leukemias (Phase I, NCT01287104, Additional file 3: Table S3, row 11). This trial was based on the preclinical observation that NK cell activation with artificial IL-15Rα- and 4-1BBL-expressing antigen-presenting cells and soluble IL-15 leads to increased expression of activating receptors and enhanced tumor killing [101, 247, 248]. Patients received one (1 × 10^5^ NK cells/kg) or two (1 × 10^6^ NK cells/kg) doses of related donor graft, or one dose (1 × 10^5^ NK cells/kg) of unrelated donor graft. While the allogeneic NK cells showed potent anti-tumor efficacy, 5 out of 9 patients experienced acute GvHD (3 with severe grade 4). As GvHD was more common in patients treated with matched unrelated donors, despite the low T cell levels, the study concluded that the allogeneic NK cells contributed to acute GvHD by increasing underlying T cell alloreactivity [249]. Increased occurrence of GvHD might be due to differences in the IL-15/4-1BBL-activated NK cell product compared to non-activated or IL-2-activated NK cells [101]. Notably, 4/5 subjects who developed GvHD received donor NK cell with the KIR inhibitory A/A haplotype (inhibitory KIRs with either KIR2DS4 or no activating KIRs), whereas 3 of the 4 subjects without GvHD had donors of the activating B/x haplotype (having any activating KIR other than KIR2DS4) (*P* = 0.1 by χ-square, 2-tailed). Furthermore, in vitro potency testing showed killing capacity against the Ewing sarcoma line TC-71, with no difference between cells administered to patients developing or not developing GvHD. Cells showed diminished killing upon blockade of NKG2D, NKp44, and NKp46 activating receptors. At 100 days post-infusion, > 95% donor lymphoid chimerism was observed in 25% of the patients receiving at least one dose of 1 × 10^6^ cells from a related donor and in 62.5% receiving at least one dose of 1 × 10^5^ cells from an unrelated donor. OS was 15.5 months (5.8–30.2) for 1 × 10^5^ cells/related donor, 34 months (6.3–36) for 1 × 10^6^ cells/related donor, and 10.5 months (6.4–36) for 1 × 10^5^/unrelated donor, with mortality rates of 100%, 85.71%, and 66.67%, respectively [249].

The Fred Hutchinson Cancer Center, together with the NCI, investigated the safety and efficacy of combining an infusion of allo-PB-NKs early after non-myeloablative haploidentical donor bone marrow transplantation in patients with hematologic malignancies (Phase I/II study, NCT00789776, Additional file 3: Table S3, row 13). Patients were treated with either 1 dose (2.5 × 10^6^ cells/kg) or 2 doses (5 × 10^6^ cells/kg) of NK cells. No dose-limiting toxicities were observed in either patient group. At 1 year post-infusion, 20% of patients receiving 1 dose and 28.6% of patients receiving 2 doses of NK cells had relapsed disease. 20% of patients receiving 1 dose also had GvHD. 11.4% of patients receiving 2 doses experienced graft failure, not observed in any patient receiving one dose. One-year survival post-transplant was 100% for the single-dose group, and 71.4% for the double dose. Chronic GvHD at 1 year was observed in 40% of the single-dose patients and in 8.6% of the double-dose group.

The Centre Hospitalier Universitaire Régional de Besançon has completed a Phase I trial on the combination of allo-PB-NK cells with anti-EGFR Cetuximab to enhance ADCC in patients with gastrointestinal cancer (NCT02845999, Additional file 3: Table S3, row 38). Of 9 patients treated, objective clinical response was observed in 3, two of which injected with NK cells with 2 KIR ligand mismatches and one with 1 KIR mismatch. The treatment was well tolerated [250].

The Memorial Sloan Kettering Cancer Center completed a Phase I trial (NCT00877110, Additional file 3: Table S3, row 42), where the safety and efficacy of allo-PB-NK cells in combination with an anti-GD2 mAb (3F8) were assessed in patients with high-risk neuroblastoma. Objective response was observed in 29% of patients, no response in 47%, and progressive disease in 23%. There was a correlation between response and KIR/HLA genotype or FcγRIII polymorphisms. Patients receiving higher doses of NK cells (> 1 × 10^7^ CD56^+^cells/kg) showed improved PFS. Median PFS and OS were 7.4 months and 30.7 months. Circulating donor NK cells could be found in 13% of patients at day 7 and in 0% at day 14 post-infusion. Interestingly, patient NK cells showed high expression levels for NKG2A inhibitory NK cell receptor, whose ligand HLA-E is highly expressed in neuroblastoma leading to NK cell inhibition [251].

The University of Arkansas and the NIH investigated the use of ex vivo expanded NK cells in combination with the proteasome inhibitor Bortezomib in patients with MM in a Phase II clinical trial (NCT01313897, Additional file 3: Table S3, row 61). Bortezomib was administered at days -9, -6, and -2 prior to NK cell infusion. NK cells from MM patients or haploidentical donors were expanded and activated ex vivo using 4-1BBL- and membrane-bound IL-15-expressing K562 [252]. Patients received either fresh or cryopreserved NK cells; interestingly, in vivo expansion was only observed in patients receiving the fresh product, with a peak at or around day 7 post-infusion. The treatment was well tolerated, with no observed toxicities related to the NK cell product. Out of 7 evaluable patients, 1 had a partial response and another showed a decreased rate of disease progression. In the remaining 5 patients, the disease progression rate was not affected by the NK cell infusion [253].

SMT bio assessed the use of allogeneic SMT-NK cells in combination with anti-PD-1 mAb Pembrolizumab for the treatment of biliary tract cancer in a Phase I/II clinical trial (NCT03937895, Additional file 3: Table S3, row 28). A treatment cycle consisted of one dose of 3 × 10^6^ SMT-NK cells/kg accompanied by Pembrolizumab administration, followed by a second dose of SMT-NK the week after. Six patients were enrolled in the pilot study and received up to 3 cycles of treatment (6 doses of NK cells and 3 of Pembrolizumab), with no severe adverse effects. Among 5 patients who finished the injections, two showed stable disease (SD) and continued the treatment, being enrolled into Phase IIa in with 34 other patients. In Phase II, up to 9 cycles were administered. Among the 20 patients who finished 6 NK cell and 3 Pembrolizumab injections (1^st^ response evaluation criteria in solid tumors, RECIST), 65% showed SD. When 12 NK cell and 6 Pembrolizumab injections finished (2^nd^ RECIST), patients had 40% and 20% SD and PR. Of the 3 patients at 3^rd^ RECIST (18 NK cell and 9 Pembrolizumab), 1 and 2 patients showed SD and PR, respectively. Adverse effects were reported for 6 patients: one grade 3 and five grade 1–2 [254].

## Field challenges

### Challenges in commercial manufacturing of NK cell therapies

As more NK cell therapeutics reach the clinical stage, investigators face major challenges in the production of NK cell products for clinical use according to GMP. As shown in the previous sections, to achieve future commercial success NK cell therapies must be manufactured at an appropriate scale with excellent product quality control, supporting the safety of the treatment for many patients, often with high doses and multiple infusions.

Currently, most NK cell manufacturing processes originate from academic research laboratories and are used in early clinical trials relying on academic current cGMP-compliant systems. Such environments are hardly suitable for the generation of commercial products; although developers often pioneer highly innovative approaches, these remain unconnected to the mainland of pharmaceutical practice and needs due to the lack of expertise and absence of facilities necessary to comply with regulations [255]. Hospital GMP facilities are often convenient to produce autologous products directly after cell isolation from patients but can also facilitate the manufacturing of allogeneic and cryopreserved products for early clinical trial evaluation [256]. Translation of a manufacturing process into an own manufacturing facility or to a contract manufacturing organization requires meeting high standards of pharmaceutical GMP to align with regulatory requirements. Cleanroom facility design and operation, personnel training, equipment validation, and material qualification must comply with guidelines and be embedded in a quality management system (QMS). A proper quality Control (QC) structure must be in place, to ensure safe and appropriate manufacture and characterization of each product batch. Defined product testing procedures and acceptance criteria need to be in place to prevent, identify, report, and manage any deviations that can generate low-quality or unsafe products. Such a gap can be filled by establishing partnerships between industry and academia, to bridge scientific knowledge with technological expertise. In line with this, several of the clinical studies currently pursued with NK cells are a result of partnerships between the public and the private sector, or from spin-off companies from academic laboratories [255].

Manufacturing capacity is another pivotal aspect. Off-the-shelf allogeneic NK cell products allow for larger batch size and to increased vessel scale (scale-up); the parallelization of several independent units (scale-out), relevant to autologous approaches, can also apply to allogeneic donor-derived sources, although it requires a thorough assessment of technical capacities [114, 257]. Manufacturing facilities require adequate space, as commercial-scale allogeneic batch cultures are expected to be between 200 and 2000 L, with 10^11^–10^14^ annual cells/indication produced [257, 258]. The choice of the appropriate cell culture vessel must be done to suit both the number of cells at the start of large-scale cultures and the numbers achieved at the end of expansion, also considering the donor-related variability still present in standardized conditions [114]. Specific production parameters must also be monitored [114], such as temperature, sheer stress, dissolved oxygen and CO_2_, osmolality, and pH. As contamination and operator errors represent the main sources of process failure, the introduction of closed, automated cell culture systems can enhance technical precision, reproducibility, and efficiency, and can improve the development of cost-effective cell products. Likely, process- (and product-) specific solutions must be further developed and implemented, but several companies are already active in providing automation solutions to manufacturers [259, 260].

Final product cryopreservation allows true fulfillment of the off-the-shelf promise, as cells can be delivered in a timely manner for patients’ needs, but also help to reduce costs and support the design of manufacturing facilities. As there is no universal formula for the cryoprotecting matrix, developers must identify the optimal medium to protect their own product during freezing and storage while maintaining high viability and cytotoxic potential after thawing [261] (discussed in the “Final formulation and preservation” section). The distribution of cryopreserved products requires hospitals to be equipped with thawing devices and to train personnel to thaw cells before infusion.

Engineered NK products require additional preparedness. Developers should carefully consider the implications of the chosen engineering method during early product development phases, as it must comply with pharmaceutical and commercial requirements and therefore could increase the complexity of the development process. For γ-retroviral and lentiviral systems, the costs of producing large quantities of GMP-grade virus remain high and often require outsourcing to third-party contract development and manufacturing organizations (CDMOs) [262].

### Regulatory and quality aspects

As the FDA Guidance for Industry: Human Somatic Cell Therapy and Gene Therapy (FDA-2009-D-0132-0016) mentions, “biological products are often complex mixtures that cannot be completely defined”; therefore, the generation (cell culture procedures) and characterization (identity, potency, viability, sterility, purity, and safety) of cell populations require appropriate quality control of the manufacturing process and of the final product [263].

Specific guidelines for potency testing (EMA/CHMP/BWP/271475/2006 rev.1 and FDA-2008-D-0520) demand the submission of data to ensure “the identity, quality, purity and strength, as well as stability” of cellular therapies [264, 265]. Products must be appropriately characterized regarding the mechanism of action of the therapeutic agent; however, there is no universal protocol on how potency assays should be conducted in vitro, and how the choice of effector to target ratio, of tumor models or of the analytical platform should be made. As NK cell products are generated from various sources, using different manufacturing processes, the resulting products are not equal and can kill tumor cells using various, distinct signals. Therefore, potency test results from different therapeutic products are difficult to compare, and standardization becomes very challenging.

Beyond potency, product composition and lot-to-lot variability must be assessed. Currently, there is a lack of universal criteria for the composition of NK cell products [261]. Phenotypic features of NK products, such as surface expression of activating or inhibitory receptors, can be assessed differently in different products. Product characterization is often based on the measurement of the average population expression of a limited number of antigens on the cell surface, or intracellularly, that are best indicative for critical product attributes [266]. Furthermore, final formulations can contain process-related impurities or impurities of other cell types, often B and T cells, which can compromise the safety of the treatment and therefore must be carefully monitored. Due to the biological heterogeneity of NK cell donors, different batches will have different impurity content, phenotype, and potency. Analytical methods and acceptance criteria must be defined to ensure manufacturing consistency and desired product quality but also to safely monitor and accommodate batch variability.

Hematopoietic stem and progenitor cells heterogeneity before collection, during enrichment, and through manufacturing can affect product quality. Mutations can cause functional effects, variations in transcriptional activity [267], and possibly in the efficiency of differentiation to functional NK cells. Additionally, variable transduction rates of different HSC subsets [267] can affect genetically engineered products. For such products, population homogeneity, identity, and batch-to-batch consistency should be addressed even more carefully. Nevertheless, CD34^+^ HSC-derived NK cell products have proven to be safe and effective [44, 45, 48, 268, 269], showing no additional risks than mature NK cell-based therapies.

On the other hand, potential tumorigenicity is still a major clinical concern for iPSC-derived cell therapies [270]. Tumorigenic risks arise from genomic instability during iPSC establishment, malignant transformation during reprogramming, and long-term differentiation culture; additionally, residual undifferentiated cells can remain as contaminants [270]. Safeguard systems which could mitigate such risks are the use of non-integrating engineering methods and the introduction of inducible suicide genes [270]. Clone selection and undifferentiated cell removal are also necessary [271]. Nevertheless, questions on the best iPSC starting material, on robustness of differentiation approaches and on optimal management of off-target risk of gene modifications, remain to be better clarified [271]. Given the risks, genomic/epigenomic abnormalities should be analyzed in both pluripotent and differentiated states, to distinguish and track iPSCs (and derived NK cells) best suited for therapeutic applications [272].

Modifications of the manufacturing system, or of the materials and reagents used, can have consequences on product quality, which must be assessed by appropriate comparability testing [273, 274]. Demonstration of comparability requires extensive product characterization and understanding of quality attributes, which are critical for product efficacy and safety. Challenges of comparability of cellular therapies have been recently reviewed by Cockroft and Wilson [275].

Specific requirements concern the generation of medicinal products containing genetically modified cells. As reported in the guideline published by the European Medicines Agency (EMA) in 2020 (EMA/CAT/GTWP/671639/2008 Rev. 1-corr), and in a recent draft guidance document from FDA “Considerations for the development of chimeric antigen receptor (CAR) T cell products” (FDA-2021-D-0404), the type of delivery vector should be appropriate and justified based on the intended cell type, the expected genomic modification and the clinical indication, and generated according to good manufacturing practice principles [276, 277]. Vector design must aim to minimize the risk of insertional mutagenesis (e.g., by using SIN vectors). The sequence of all plasmids (for viral-based engineering) and coding and noncoding DNA molecules (for production of recombinant mRNA or proteins) must be verified. For CAR-engineered products, the ability of the antigen recognition domains to bind their target should be assessed, and the impact of humanization of murine elements, reducing the risk of immunogenicity in humans, on target binding and biological activity should be determined. Signaling domain functionality should be thoroughly demonstrated. Unrelated sequences used for identification and selection, such as tags, should be avoided unless justified. For all genetically engineered products, a risk assessment should be presented to ensure the absence of replication-competent virus (RCV) from the final product, to address the potential occurrence of off-target modifications and of vector integration profiles and sites. The risk-based approach should also be applied to the design of the manufacturing process, to assess critical attributes and parameters and to increase the assurance of producing batches of the intended quality. The risk of cross-contamination can be minimized by handling one viral vector in one clean room suite at a given time; if the same facility is shared between products manufactured with different vectors, their production could be staggered to avoid simultaneous manipulation [262]. As this can impact timelines for product availability and clinical testing, careful planning is required. Culture conditions, activation steps, engineering media, vector concentration and multiplicity of infection (MOI), and transduction efficiency may result in qualitative/quantitative differences in the final product and in the impurities present [276]. Transfection/transduction efficiency should be justified in relation to clinical efficacy data; population purity and identity must be carefully monitored for the presence of contaminant non-engineered cells of the intended type (NK cells), but also for engineered and non-engineered cells of other types (e.g., B or T cells) in the final product. Potency of CAR products should be assessed with orthogonal methods, ensuring that the activity of each functional element is assayed; for example, if a cytokine transgene is included, investigators should develop a potency assay to measure the activity of such cytokine, in addition to the assay(s) assessing CAR activity [277].

In order to limit clinical risks deriving from insertional mutagenesis, regulatory agencies have limited the acceptable number of transgene integrations. FDA recommends limiting the vector copy number (VCN) to under 5 copies per genome [278]; the most recent updates on CAR-T products recommend that VCN is determined as a function of CAR-expressing cells [277]. However, the chosen approach for VCN evaluation (bulk population or single cell analysis) can result in heterogeneous and incomparable results from different medicinal products [278, 279]. In addition, specific challenges are associated with CRISPR-Cas9-edited products, which are entering clinical development also with NK cells. Mutagenesis, genomic rearrangements, and off-target effects could affect safety and treatment outcomes; therefore, alignment between developers and regulatory bodies is recommended [280].

The entire history of each product batch, from the manufacturing process to QC testing, must be recorded and collected in an organized manner in the batch process record (BPR) [281]. The BPR must record each processing step, including identity of personnel, specific equipment and consumables with lot numbers, donor material arrival, processing, storage, and release description. The plan and implementation of the recording system must be considered as important as the actual product development. The BPR must document whether a certain batch did or did not meet acceptance criteria, if it was released for distribution or quarantined [281]. A searchable system, where each batch, date, consumable lot or equipment are uniquely identified, must be available for consultation and for inspection from authorities; therefore, maintaining an accurate documentation system is critical. Paper-based BPR is adequate but is not practical for large facilities enabling manufacturing at a commercial scale. Electronic data management tools are available and should be preferentially implemented. Notably, BPR must be protected to ensure donor privacy and to prevent loss in case of disaster (flooding, fire).

More challenges are associated with the logistics of NK cell manufacturing. The availability of raw materials and disposables and shortage of technical staff can only partially be anticipated [282]. Additional challenges may arise depending on specific product features and must be addressed in the target product profile (TPP). Academic and private investigators attempting clinical manufacturing of NK cell products must consider scale and quality aspects from the early stages of planning. Successful development of NK-based cell therapies lies beyond the product’s biological features, but deals with cost sustainability, production capacity, batch size, product storage system, and compliance with regulatory authorities’ requests, to advance to pivotal clinical stages and achieve product market approval.

## Conclusions and future directions

This review summarizes how allogeneic NK cell-based cancer immunotherapies have demonstrated to be a concrete opportunity for the treatment of hematological and solid tumors. Clinical evidence suggests that NK cell therapies show high safety and efficacy and can fulfill the promise for potent, universal, and off-the-shelf immune cell therapies, overcoming some of the drawbacks of CAR-T therapies. However, challenges remain. Limited in vivo persistence, albeit contributing to the safety profile of NK cell therapies, can restrict therapeutic efficacy. The use of off-the-shelf, allogeneic products can overcome this issue, as multiple doses can be infused into patients with the appropriate timing aiming to maximize the response. Treatment of solid tumors is restricted by limited infiltration and by the escape of the immune suppressive tumor microenvironment. Although efforts are made to treat solid tumors with non-engineered NK cells, alone or in combination therapies, more and more investigators are tackling them with engineered NK products, especially in combination. NK cell engineering with CARs improves tumor infiltration and targeting [283], in addition to their innate killing capabilities; expression of cytokines, either by means of genetic engineering or as a combination with priming agents, can improve persistence and function. A promising approach is the deletion of the CISH gene, coding for the negative regulator of IL-15 signaling CIS, which leads to improved NK cell metabolic profile, increased in vivo persistence and anti-tumor function of NK cells [284]. Additionally, increased anti-tumor responses are achieved by combinations of NK cells with tumor-targeting antibodies and checkpoint inhibitors. Novel combination approaches are also reaching the clinical stage, such as NK cell engagers, migration enhancers, immunomodulators targeting the suppressive tumor microenvironment, and agents aiming to reverse tumor immune escape. A comprehensive summary of the next-generation NK cell product design has been recently published by Laskowski and colleagues [285].

Alongside therapeutic efficacy, cell production must be adequate for the foreseen product market. Scaling-up and -out urgently requires technological advances that can be achieved only in parallel with product characterization and standardization. The GlobalData database reports over 35 partnerships for NK cell product co-development (26 established only between 2020 and 2021), 18 of which involve university and company partners. Notably, the CAR-NK product developed by Rezvani’s team at MD Anderson Cancer Center was announced to enter an exclusive licensing agreement with Takeda in November 2019 [286, 287]. Takeda gained access to MD Anderson Cancer Center’s CAR-NK platform to accelerate the development of the CD19 CAR-NK product TAK-007, and to develop additional programs. Although the first 11 patients treated (NCT03056339, Additional file 2: Table S2, row 12) could be supplied with cells manufactured in the cancer center's facility, further clinical development and feasibility for commercialization are taken over by a biopharmaceutical organization. In-house GMP manufacturing is pursued by biotech companies such as Glycostem Therapeutics, which has developed the uNiK™ closed system to produce stem cell-derived NK cells [288]. Alternatively, Miltenyi Biotech offers the CliniMACS Cell Factory®, a scalable model of cell manufacturing, providing instruments, reagents, and expertise, to help investigators overcome manufacturing challenges. Although this approach is currently applied to autologous CAR-T products, it could also be applied to NK cell manufacturing [289]. As several early clinical studies sponsored by academic institutions or hospitals are expected to be followed up by larger, more advanced trials, it is likely that more partnerships with biotech and pharmaceutical companies will be signed in the future. A combination of the solid therapeutic potential of NK cells with excellent technology for product development and adequate funding will be the key to finally open the gates of market authorization.

## Supplementary Information

**Additional file 1: Table S1**. List of clinical trials with non-engineered NK cell therapies. *N* = 36 clinical trials (Phase I, II, or I/II) evaluating the infusion of non-engineered allogeneic NK cell therapies in hematological or solid tumor patients were registered on ClinicalTrials.gov between March 2017 and December 2021. Studies are sorted by NK cell source (PB-NK, UCB-CD34, UCB-NK, iPSCs). The most relevant product characteristics and the clinical trial design and outcome (when available) are presented. Trial status is updated to August 2022. PB: peripheral blood; UCB: umbilical cord blood; iPSCs: induced pluripotent stem cells.

**Additional file 2: Table S2**. List of clinical trials with engineered NK cell therapies. *N* = 53 clinical trials (Phase I, II, or I/II) evaluating the infusion of engineered NK cell therapies in hematological or solid tumor patients were registered on ClinicalTrials.gov until 31–12–2021. Studies are sorted by NK cell source (PB-NK, UCB-NK, iPSCs, NK-92, Unknown). The type of engineered NK products (scFv-CAR-NK, Receptor-CAR-NK, CD16-engineered, or CD16- and scFv-engineered CAR-NK), the engineering method, transgene structure, and the clinical trial design and outcome (when available) are presented. Trial status is updated to August 2022. PB: peripheral blood; UCB: umbilical cord blood; iPSCs: induced pluripotent stem cells; scFv: single-chain variable fragment.

**Additional file 3: Table S3**. List of clinical trials with non-engineered combination NK cell therapies. *N* = 62 clinical trials (Phase I, II, or I/II) evaluating the infusion of non-engineered allogeneic NK cells in combination with other agents were registered on ClinicalTrials.gov until 31–12–2021. Studies are sorted by types of combination therapy (NK cell priming agents, Adoptive cell therapy, Antibodies, Co-stimulation, Multiple combinations, Molecular inhibitors and NK cell engagers). Details of the combination approach and the clinical trial design and outcome (when available) are presented. Trial status is updated to August 2022.

**Additional file 4: Table S4**. List of clinical trials with engineered combination NK cell therapies. *N* = 34 clinical trials (Phase I, II, or I/II) evaluating the infusion of engineered allogeneic NK cells in combination with other agents were registered on ClinicalTrials.gov until 31–12–2021. Studies are sorted by types of combination therapy (Antibodies, Multiple combinations). Details of the combination approach and the clinical trial design and outcome (when available) are presented. Trial status is updated to August 2022.

## Data Availability

Not applicable.

## Abbreviations

NK: Natural killer
MHC: Major histocompatibility complex
TRAIL: Tumor necrosis factor-related apoptosis-inducing ligand
RCC: Renal cell carcinoma
KIR: Killer immunoglobulin-like receptor
HLA: Human leukocyte antigen
AML: Acute myeloid leukemia
GvHD: Graft-versus-host disease
IL: Interleukin
CRC: Colorectal cancer
HCC: Hepatocellular carcinoma
PB: Peripheral blood
UCB: Umbilical cord blood
HSC: Hematopoietic stem cell
iPSC: Induced pluripotent stem cell
GMP: Good manufacturing practice
G-CSF: Granulocyte colony-stimulating factor
HSCT: Hematopoietic stem cell transplant
INN: International nonproprietary name
NHL: Non-Hodgkin lymphoma
ADCC: Antibody-dependent cellular cytotoxicity
SCGM: Stem cell growth medium
GBGM: Glycostem basal growth medium
FBS: Fetal bovine serum
HS: Human serum
HANK: Highly activated NK cells
SFEM: Serum-free expansion medium
CIML: Cytokine-induced memory-like NK cells
SCF: Stem cell factor
Flt3L: FMS-like tyrosine kinase 3 ligand
TPO: Thrombopoietin
GM-CSF: Granulocyte macrophage colony-stimulating factor
AHR: Aryl hydrocarbon receptor
SR1: StemRegenin 1
NAM: Nicotinamide
GSK-3B: Glycogen synthase kinase 3β
HSP70: Heat-shock protein 70
PBMC: Peripheral blood mononuclear cell
mAb: Monoclonal antibody
mbIL21: Membrane-bound IL-21
aAPC: Artificial antigen-presenting cell
FC21: Feeder cell 21
PM21: Plasma membrane 21
EBV-LCL: Epstein–Barr virus-transformed lymphoblastoid cell line
NIH: National Institutes of Health
CB-MNC: Cord blood mononuclear cell
HSA: Human serum albumin
CPA: Cryoprotectant agent
DMSO: Dimethyl sulfoxide
PBS: Phosphate saline buffer
MDS: Myelodysplastic syndromes
MM: Multiple myeloma
NSCLC: Non-small-cell lung cancer
HNC: Head and neck cancer
BTC: Biliary tract cancer
MCC: Merkel cell carcinoma
CAR: Chimeric antigen receptor
ITAM: Immunoreceptor tyrosine-based activation motif
FDA: Food and Drug Administration
DLBCL: Diffuse large B cell lymphoma
ALL: Acute lymphoblastic leukemia
CRS: Cytokine release syndrome
TAA: Tumor-associated antigen
TME: Tumor microenvironment
MDSCs: Myeloid-derived suppressor cells
CCCRs: Chimeric co-stimulatory converting receptors
LTR: Long terminal repeats
SIN: Self-inactivating virus
MFI: Mean fluorescence intensity
VCN: Vector copy number
hnCD16: High-affinity, non-cleavable CD16
scFv: Single-cell variable fragment
TCR: T cell receptor
HAMA: Human anti-mouse antibody
CDR: Complementarity-determining regions
NCR: Natural cytotoxicity receptor
Fc: Fragment crystallizable
Ig: Immunoglobulin
ADAM17: A disintegrin and metalloprotease
haNK: High-affinity NK
t-haNK: Target high-affinity NK
mbIL-15: Membrane-bound IL-15
IL-15RF: IL-15 receptor fusion
ER: Endoplasmic reticulum
ERIL-2: ER-retained IL-2
FKBP: FK506-binding protein
CID: Chemical inducer of dimerization
CRISPR: Clustered regularly interspaced short palindromic repeats
CML: Chronic myelogenous leukemia
HL: Hodgkin lymphoma
CLL: Chronic lymphocytic leukemia
B-ALL: B cell acute lymphocytic leukemia
MoA: Mechanism of action
GvL: Graft versus leukemia
HNSCC: Head and neck squamous cell carcinoma
TKI: Tyrosine kinase inhibitor
NKCE: NK cell engager
NSG: NOD scid gamma
SITC: Society for Immunotherapy of Cancer
OR: Objective response
CR: Complete response
OS: Overall survival
MRD: Minimal residual disease
PR: Partial response
PFS: Progression-free survival
MFC: Multiparameter flow cytometry
NGS: Next-generation sequencing
aNK: Activated NK
PD: Progressive disease
TNBC: Triple-negative breast cancer
SCLC: Small-cell lung cancer
DFS: Disease-free survival
RFS: Relapse-free survival
NCI: National Cancer Institute
IRE: Irreversible electroporation
PET-CT: Positron emission tomography and computed tomography
HCT: Hematopoietic cell transplantation
CMR: Complete metabolic response
SD: Stable disease
RECIST: Response evaluation criteria in solid tumors
QMS: Quality management system
QC: Quality control
CDMO: Contract development and manufacturing organization
EMA: European Medicines Agency
RCV: Replication-competent virus
MOI: Multiplicity of infection
BPR: Batch process record
TPP: Target product profile
4-1BBL: 4-1BB ligand
DMEM: Dulbecco's modified Eagle's medium
dMMR: Mismatch repair deficiency
EGFR: Epidermal growth factor receptor
FCS: Fetal calf serum
FL: Follicular lymphoma
GBM: Glioblastoma multiforme
GEJ: Gastroesophageal junction
Gy: Gray
HER2: Human epidermal growth factor receptor 2
MSI-H: Microsatellite instability
RPMI-1640: Roswell Park Memorial Institute medium
α-MEM: α-Minimum essential medium
